# Supplementary material for: VolcanoFinder: Genomic scans for adaptive introgression
Source: PLoS Genet. 2020 Jun 18;16(6):e1008867. doi: 10.1371/journal.pgen.1008867 (PMC7326285; doi:10.1371/journal.pgen.1008867)
Supplement: S1 Appendix — Supplementary material for the model and analysis, organized into the following sections: (1) Analytic model (2) Model two—accounting for coalescent time within the recipient population (3) The SFS after the introgression sweep (4) Comparison of models one and two (5) Performance of VolcanoFinder and SweepFinder models. (PDF) [file pgen.1008867.s001.pdf]

VolcanoFinder

genomic scans for adaptive introgression

S1 Model and Analysis

Derek Setter[1,2,3,◕,¤], Sylvain Mousset[1,◕], Xiaoheng Cheng[4], Rasmus Nielsen[5], Michael DeGiorgio[6,‡], Joachim Hermisson[1,2,7,‡],

1 Department of Mathematics, University of Vienna, Vienna, Austria
2 Vienna Graduate School of Population Genetics, Vienna, Austria
3 School of Biological Sciences, University of Edinburgh, Edinburgh, United Kingdom
4 Huck Institutes of the Life Sciences, Pennsylvania State University, University Park, PA, USA
5 Departments of Integrative Biology and Statistics, University of California, Berkeley, CA, USA
6 Department of Computer and Electrical Engineering and Computer Science, Florida Atlantic University, Boca Raton, FL, USA
7 Max F. Perutz Laboratories, University of Vienna, Vienna, Austria

◕These authors contributed equally to this work.
‡These authors also contributed equally to this work.
¤Current Address: School of Biological Sciences, University of Edinburgh, Edinburgh, United Kingdom
*Correspondence: joachim.hermisson@univie.ac.at (JH), mdegiorg@fau.edu (MD)

S1 Model and Analysis

Text S1.1

Analytic Model

Here, we compare different approximations for the effects of selection on the genealogical distribution at a linked neutral site. As in the main text, s is the (heterozygous) strength of selection acting on the beneficial B allele, $R = rd$ is the rate of recombination between the two sites, and we sample $n = 2$ individuals from a diploid population of size N.

Star-like approximation In the main text, the star-like approximation assumes that the stochastic trajectory of the B allele is well approximated by the expected change in allele frequency, *i.e.* logistic growth, from an initial frequency of $1/(2N)$. At a single time point, this average is taken over all possible changes in allele frequency, including a decrease which causes loss of the B allele. In that way, at low frequency, the expected growth is very slow. However, when the allele frequency is very small, its fate – loss or fixation – is largely stochastic. By conditioning on fixation of the B allele, we tend to observe cases where, by chance, the B allele increases in frequency faster than expected. This early stochastic increase can be accounted for by setting the initial frequency to $1/(2N2s)$ [1]. The expected time to fixation is $2\ln(2N2s)/s$, and the probability of escape becomes

$$P_e = 1 - e^{-\frac{R\ln(2N2s)}{s}}. \tag{S1.1}$$

This amounts to re-scaling $\alpha d \to \frac{R\ln(2N2s)}{s}$ in the main text. The same result for P_e was also derived by [2,3] using a diffusion-approximation approach. The effect of rescaling αd is that the predicted breadth of the sweep increases to fit simulation results more closely. However, this does not account for the fact that P_{Bb} is overestimated while P_B is underestimated by the star-like approximation (Fig. A1).

Dealing with variance in coalescence time The fault of the star-like approximation falls in assuming all coalescence occurs at the very beginning of the sweep. In reality, there is variance in the true time to coalescence for the sampled lineages, and late recombination events permit coalescence even to the b background. While this variance in coalescence time can be addressed using a diffusion approach [4], this approximation is valid only for small values of R/s. For accurate predictions over the full breadth of the volcano sweep (see Fig. A1), we use the approximation in [5], re-derived for the Wright-Fisher (WF) model:

$$P_e = 1 - \frac{s}{R(1-2s)+s}\prod_{j=2}^{M}\left(1-\frac{R}{js}\right)$$

$$\begin{aligned}
P_B &= \frac{s}{R(1-2s)+s}\prod_{j=2}^{M}\left(1-\frac{2R}{(j+1)s}\right) \\
P_b &= \frac{R(1-2s)}{R(1-2s)+s}\prod_{j=2}^{M}\left(1-\frac{2R}{(j+1)s}\right) + \sum_{i=2}^{M}\frac{2R}{i(i+1)s}\prod_{j=i+1}^{M}\left(1-\frac{2R}{(j+1)s}\right) \\
P_{Bb} &= 2\left((1-P_e)-P_B\right) \\
P_{bb} &= 1-P_B-P_b-P_{Bb}
\end{aligned} \tag{S1.2}$$

Here, the establishment of the beneficial allele is modeled as a continuous-time branching process, where the intrinsic birth and death rates are taken as 1/2 (rather than 1) to account for drift in the WF (rather than Moran) model [6]. This leads to our term of $R(1-2s)$ rather than $R(1-s)$ in [5]. The subsequent growth of the beneficial allele, conditioned on fixation, is modeled as a pure-birth branching process, or Yule process, which is marked by recombination events to account for the effects of selection on the genealogy at linked neutral sites. If the number of lineages sampled after the sweep is small, their ancestry is well-approximated by the growth of the marked Yule process from the single initial B lineage to $M = 2Ns$ (Moran) or $M = 2N2s$ (WF) lineages. Aside from this factor-of-two difference in M, however, the rates of events in the conditioned process are the same in the Moran and WF models.

In Fig. A1, we see that the star-like approximation for $(1-P_e)$, eq. (S1.1), slightly overestimates this but otherwise performs almost as accurately as eq. (S1.2). For comparison, we follow [5] in approximating

$$(1-P_e) \approx e^{-\frac{R}{s}(\ln(2N2s)+\gamma-2s)} \tag{S1.3}$$

where $\gamma \approx 0.58$ is Euler's gamma. Note that if we ignore γ and s terms, we recover the star-like approximation by [1]. This may be interpreted as a better approximation for the time to fixation of the beneficial allele. Indeed $\frac{2R}{s}(\ln(2N2s)+\gamma-2s)$ closely resembles the approximations in [7] and [6] for the expected time to fixation.

On the other hand, we see in supp. Fig. A1 that P_B is underestimated by the star-like approximation, and as a consequence, $P_{Bb} = 2((1-P_e)-P_B)$ is overestimated. In contrast, eq. (S1.2) estimates P_B very well. We may similarly approximate P_B, and by rearrangement and substitution using $1-P_e$ in eq. (S1.3), we find

$$P_B \approx (1-P_e)^2 e^{\frac{2R}{s}(1+s)}. \tag{S1.4}$$

The $(1-P_e)^2$ term corresponds to that of the star-like approximation using $\alpha d = \frac{2R}{s}(\ln(2N2s)+\gamma-2s)$ as the sweep strength parameter. Importantly, eq. (S1.4) shows us that P_B cannot be accurately approximated using the single sweep parameter α. Rather, P_B is $e^{\frac{2R}{s}(1+s)}$ times higher than expected under the star-like approximation.

Let $(1-P_e)^2$ account for coalescence which occurs at the origin of the sweep and denote $P^* = \frac{(1-P_e)^2}{P_B} = e^{-2R\frac{1+s}{s}}$ the proportion of the $\{B,B\} \to \{B\}$ events which are approximately star-like. Very near the selected site, few if any lineages escape the sweep and most coalescent events will occur very near the origin of the B allele, *i.e.*, as $R \to 0$, $P^* \to 1$ and $P_B \to (1-P_e)^2$. As R increases, one or both lineages are likely to escape the sweep. Conditioned on sampling lineages with a $\{B,B\} \to \{B\}$ genealogy, coalescence during the sweep occurs only among the subset of non-recombinant B type lineages. P^* decreases to 0 as R grows, and therefore if coalescence occurs, it does so earlier in the sweep than expected under the star-like approximation. However, note that the relative error of the star-like approximation increases with $2R(1+s)/s$, but P_B decreases more quickly, approximately with $2R\ln(2N2s)/s$. That is, at distances where the error becomes large, P_B is already very small.

Fig. A1

The effect of selection on linked neutral genealogies. The probability for a lineage not to escape $(1 - P_e)$ and the genealogical distribution for a sample of $n = 2$ as a function of the recombination rate R. The solid lines are the approximation of eq. (S1.2). The dashed lines use the star-like approximation with P_e as in S1.1. The dots represent the average from 1 000 independent simulation runs. **A**. $N = 5\,000$. **B**. $N = 1\,000$. In both panels $s = 0.1$.

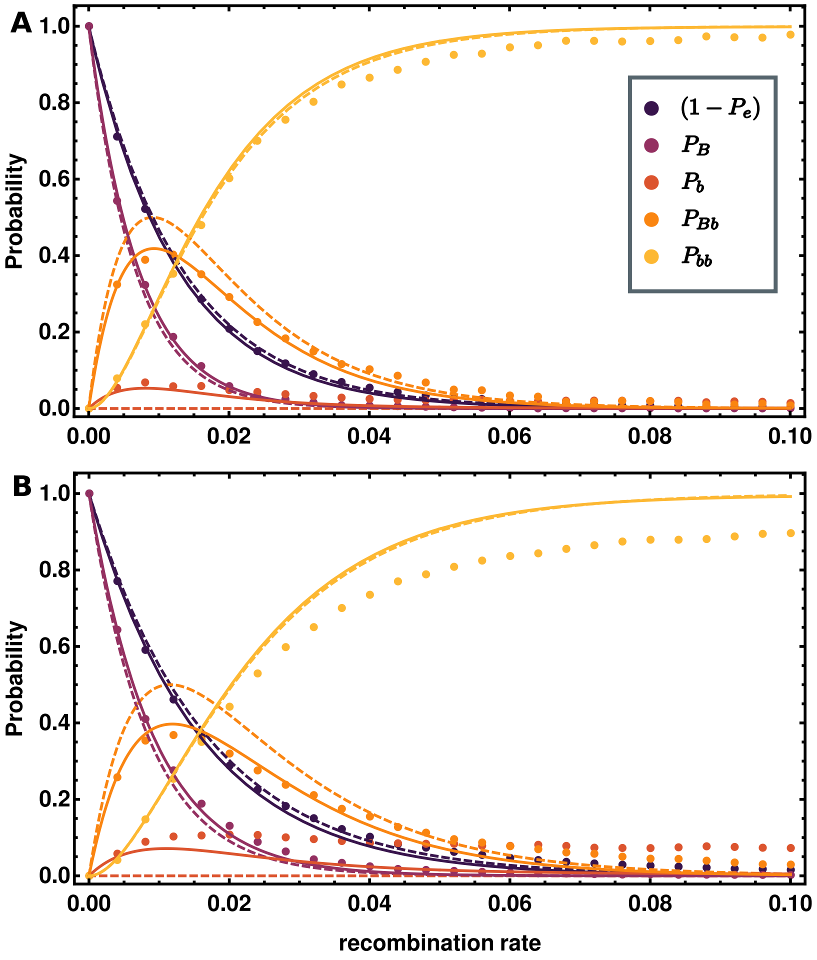

Fig. A2

Single iterations of an adaptive introgression event. Each panel shows an independent, randomly chosen simulation run. We calculated the whole-population mean genetic diversity in 401 non-overlapping non-adjacent one kb windows separated by one kb and centred on the selected locus. The initial heterozygosity and the genetic diversity at fixation of the beneficial B allele are shown in grey and black, respectively. Here, $\theta = 0.002$ ($N = 5\,000$, $\mu = 10^{-7}$), $r = 10^{-7}$, $T_d = 6$ ($D = 13\theta$), and $s = 0.06$ ($2Ns = 600$).

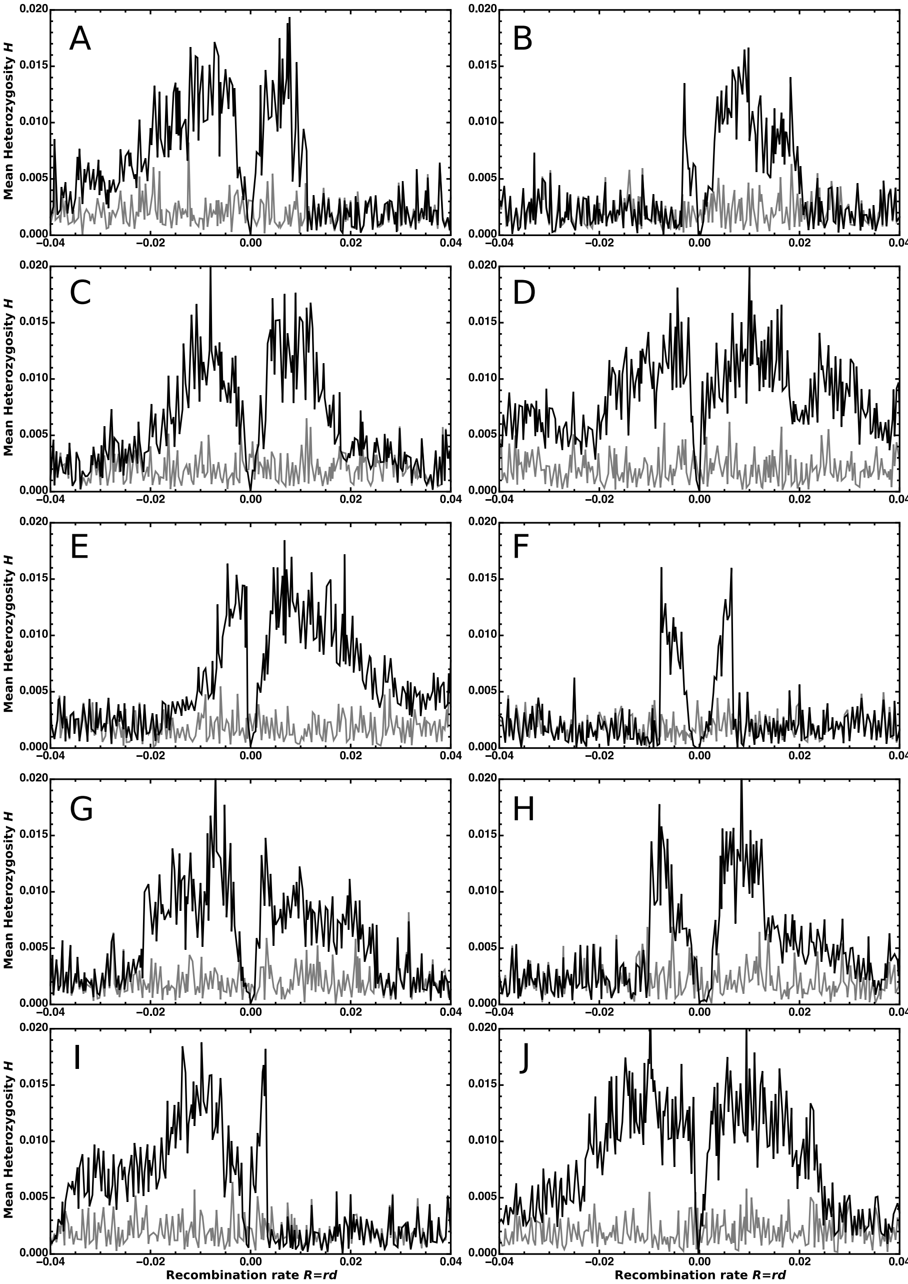

Text S1.2

Model 2: accounting for coalescence time within the recipient species.

In a second model we still assume complete lineage sorting, *i.e* all the lineages escaping the introgression sweep coalesce in a single lineage in a recipient species before this lineage coalesce with the single lineage that traced back into the donor species (see Fig. 1), but we no-longer ignore the coalescence time within the recipient species. The $D/2$ factor in the last term in eq. (9) no longer holds and thus needs to be replaced by the probability $\sigma_i(i)$ that a mutation occurred in the ancestral lineage of the i lines that escaped the introgression sweep between the common ancestor and the coalescence event with the lineage that traced back into the donor species. Inspired by eq. (12) we can express the divergences between the recipient and the donor species and between the recipient species and its MRCA with the outgroup species considering the SFS in the subsample of i lineages that escaped the introgression sweep. In the case when fixed differences are polarized we have,

$$\frac{D}{2} = \sigma_i(i) + \sum_{j=1}^{i-1} \frac{j}{i} S_j(i),$$

and

$$D_o = S_i(i) + \sum_{j=1}^{i-1} \frac{j}{i} S_j(i).$$

From these expressions we can isolate $\sigma_i(i) = D/2 - D_o + S_i(i)$ and finally get the probabilities in the altered SFS after the introgression sweep for the polymorphic states $(1 \leqslant i \leqslant n-1)$,

$$S'_i(n|\alpha,d,D) = \left(\sum_{k=i+1}^{n} P_e(k|\alpha,d) S_i(k)\right) + P_e(n-i|\alpha,d)\frac{D}{2} + P_e(i|\alpha,d)\left(\frac{D}{2} - D_o + S_i(i)\right). \tag{S1.5}$$

Eq. (S1.5) is only valid if $D/2 - D_o + S_i(i) \geqslant 0$ for all $i \in \{1,\ldots,n\}$. A necessary and sufficient condition is $\frac{D}{2} \geqslant D_o - S_n(n)$. Using eq. (12) leads to $\frac{D}{2} \geqslant \frac{n-1}{n}\hat{\theta}_L$, where $\hat{\theta}_L = \frac{1}{n-1}\sum_{i=1}^{n-1} i S_i(n)$ is an unbiased estimator of θ defined in [8, eq.(8)] and computed from the whole genomic background.

Similarly, the D_o factor in the last term of eq. (11) can easily be replaced by the probability $S_n(n)$ that a mutation occurs on the ancestral lineage of n lineages that have escaped the introgression sweep before coalescence occurs with the outgroup:

$$S'_n(n|\alpha,d,D) = \left(\left(D_o - \tfrac{D}{2}\right)\sum_{k=1}^{n-1} P_e(k|\alpha,d)\right) + D_o P_e(0|\alpha,d) + S_n(n) P_e(n|\alpha,d). \tag{S1.6}$$

If fixed differences are not polarized, then eqs. (S1.5) and (S1.6) still hold when substituting the divergence between the recipient species and its MRCA with the ougroup species D_o with the full divergence between the recipient and the outgroup species D'_o. Once again the probabilities in eqs. (S1.5) and (S1.6) are linearly dependent of the mutation parameter $\theta = 4N\mu$, and this dependency disappears in conditional probabilities obtained from eqs. (10) and (13).

Text S1.3

The SFS after an introgression sweep

Fig. A3 displays the effect of adaptive introgression on the SFS (sample size $n = 8$) of the recipient population in the simple model described above (Model 1, red columns) and the more complex model described in the supplement (Model 2, blue columns, see Text S1.2) relative to the neutral spectrum (black). Model 2 differs from Model 1 in that it does not ignore the coalescence time in the recipient species. The figure shows that near the sweep center (distance $\alpha d = 0.01$, top panel), hitchhiking reduces polymorphism and increases the proportion of fixed differences. For sites located at distances where single recombination events are likely ($\alpha d = 0.1$ and 1.0), partial hitchhiking of foreign variation increases polymorphism relative to the neutral expectation. This increase in diversity is accompanied by a decrease in the proportion of fixed differences relative to the third, outgroup species. Under the infinite sites mutation model, sites that diverge from the donor population must also diverge from the outgroup species. At these sites, introgression re-introduces the ancestral variant, sharply reducing the proportion of fixed derived sites in the sampled lineages. This is a key feature not seen in classic hard sweeps.

Fig. A3 also shows that the predicted SFS under the simple Model 1 (red) does not differ much from the SFS predicted under the slightly more accurate Model 2 (blue). However, there is still a key difference. Model 2 is restricted to $D \geqslant 2\frac{n-1}{n}\hat{\theta}_L$, whereas Model 1 may take smaller values, including $D = \theta$ for a classic sweep from *de novo* mutation. For these reasons, we suggest to, in general, use Model 1. Unless otherwise noted, the `VolcanoFinder` results presented in this article are generated under Model 1 and fixed differences with the outgroup are polarized with the help of a second, distantly-related outgroup.

Text S1.4

Comparison of Models 1 and 2

In Fig. A3, we saw that Models 1 and 2 yield similar predictions for the SFS after the selective sweep when $D \gg \theta$. That is, when the divergence is sufficiently large so that ancestral variation is no longer segregating in the populations, $T_{coal,2} \ll T_d$, the pairwise diversity D well-approximates the contribution of fixed derived mutations from the recipient population. Here, we look at the difference between the two models in approximating the expected heterozygosity after the sweep.

For a sample of $n = 2$ two lineages taken directly after the sweep, the expected heterozygosity may be approximated as in eq. (3) of the main text. In Fig. A3, we show the star-like approximation in grey, the more-accurate approximation of [5] in black, and simulation results as black dots. For a sample of $n > 2$, we use the un-normalized $S'_i(n)$ from either Model 1 (eq. 9) or 2 (eq. S1.5) to determine the effects of the sweep. By substituting the $S'_i(n)$ into eq. 6, the expected heterozygosity is given by $S'_1(2)$. We show the predictions of Model 1 (red, dashed) and Model 2 (blue, dashed) in Fig. A4. We see that Model 2 exactly matches the predictions of the star-like approximation and that Model 1 is even more biased to over-estimates the increase in genetic diversity.

Text S1.5

The performance of `VolcanoFinder` and `SweepFinder`

Here we take a closer look at the ability of both `SweepFinder` and `VolcanoFinder` to detect an adaptive introgression sweep. 200 successful introgression sweeps centered in a 500 kb locus were simulated under strong selection, $2Ns = 1\,000$ ($N = 5\,000$, $s = 0.1$), a scaled mutation parameter $\theta = 0.002$ (mutation rate $\mu = 10^{-7}$), and significant divergence of the donor population $D = 0.026 = 13\theta$. The per-site recombination rate $r = 5 \times 10^{-7}$, thus the sweep parameter $\alpha = r\ln(2N)/s = 4.6 \times 10^{-5}$. The data consists of $n = 10$ lineages sampled from the recipient species. It is polarized to an outgroup with pairwise divergence $D_o = 0.05$ and includes fixed differences. As shown in the power analysis of the main text, both sweep scan methods have high power to detect introgression sweeps with these parameters.

Single iterations of the adaptive introgression process may produce the expected volcano pattern, but when early recombination events occur, the signal is concatenated. In order to compare data to theory, the 200 iterations were combined into a single data-rich locus, preserving the unique identifier and correct positions of the mutations within the simulated genomic region The result is an "average" data set representative of the expected volcano sweep pattern which we scan for selection using `SweepFinder` and `VolcanoFinder`. Note that here, the LR values are inflated because they are calculated from the combination of 200 iterations of data. The LR values of a single iteration are much smaller.

`VolcanoFinder` scans were run over a range of potential divergence values $D = 0.010$, 0.015, 0.020, 0.026, 0.030, 0.035, 0.040, and 0.045 (*i.e.* $D/\theta = 5$, 7.5, 10, 13, 15, 17.5, 20, 22.5) including the true value used in simulations $D_{sim} = 0.026$. While the true value $D = D_{sim}$ results in a very high LR value of 507297, `VolcanoFinder` finds that an introgression sweep with $D = 0.020$ fits the average data slightly better, with LR value 598280. As shown in the previous section, model 1 consistently over-estimates the contribution of divergence before the sweep to diversity after the sweep. In combination, the star-like approximation for the effect of selection on linked neutral loci systematically over-estimates the height of the diversity peaks. Together, this indicates that a lower D value yields a better-fitting model.

Indeed, supp. Fig. A5 shows that at the distance $\alpha d = 1/2$ where the volcano peak is close to the maximum height, the model predictions fit the data better using $D = 0.02$. However, for both D values, `VolcanoFinder` finds an optimum sweep parameter $\hat{\alpha} \approx 3.4 \times 10^{-5}$, close to the true value of 4.6×10^{-5}, ($\frac{\hat{\alpha}}{\alpha} \approx 0.74$).

`SweepFinder` is also able to detect the introgression sweep but is less sensitive to the signal, producing a LR value of 2672, nearly 200 times smaller than that reported by `VolcanoFinder`. While `VolcanoFinder` uses information from the full breadth of the introgression sweep, the `SweepFinder` model cannot account for the influx of foreign variation and is sensitive only to the features of the narrow diversity valley near the introgression sweep center.

In Fig. A6, we compare the optimum model that `SweepFinder` fits to the average data set to that of `VolcanoFinder` with $D = 0.026$, discussed above and shown in Fig. A5 (right column). In the top panel, we see that `SweepFinder` detects only a very small sweep valley that approximately matches the valley of the volcano sweep. In contrast to `VolcanoFinder`, the optimum sweep strength found by this method is an order of magnitude smaller than the true value used in simulations ($\frac{\hat{\alpha}}{\alpha} \approx 6.4$).

As expected, the weak sweep parameter chosen by `SweepFinder` as the optimum allows it to approximately fit the SFS very near the sweep center (top two panels, classic sweep model $\hat{\alpha} d = 0.01$ or 0.1). At greater distances, `SweepFinder` cannot predict the effect of introgression on the SFS and matches the data poorly. Due to the weak sweep strength, the corresponding regions in the simulated data are in-reality an order of magnitude closer to the sweep center when distance is scaled by the true strength of the selective sweep. This has conflicting effects on the power of `SweepFinder` to detect adaptive introgression sweeps.

At distances $\alpha d > 10$ from the sweep center, a selective sweep has little effect on neutral genealogies, and this provides a limit to how much of the data is informative for selective sweep scans. With a much weaker selection strength parameter, `SweepFinder` assesses only a fraction of the information that is accessible to the `VolcanoFinder` method, explaining in-part the much-lower LR value.

However, by finding a weak optimum strength parameter, `SweepFinder` avoids looking at regions of the introgression sweep in which it performs poorly *relative to the background SFS used as a null hypothesis*. In the main text, we saw that at distances greater than or equal to $\alpha d = 1$, the classic sweep model predicts a near-return to the background SFS. At greater distances, sites are no longer informative due to the similarity in the null and alternative hypotheses of the likelihood ratio statistic.

Fig. A3

The site frequency spectrum after adaptive introgression.

The SFS for a sample of $n = 8$ as a function of the relative distance αd from the sweep center. Model 1 (eq. 11) predictions are in red; Model 2 (eq. S1.6), blue. Here, $N = 5\,000$, $s = 0.1$, $\theta = 0.005$, $D = 0.026$ and $D_o = 0.05$

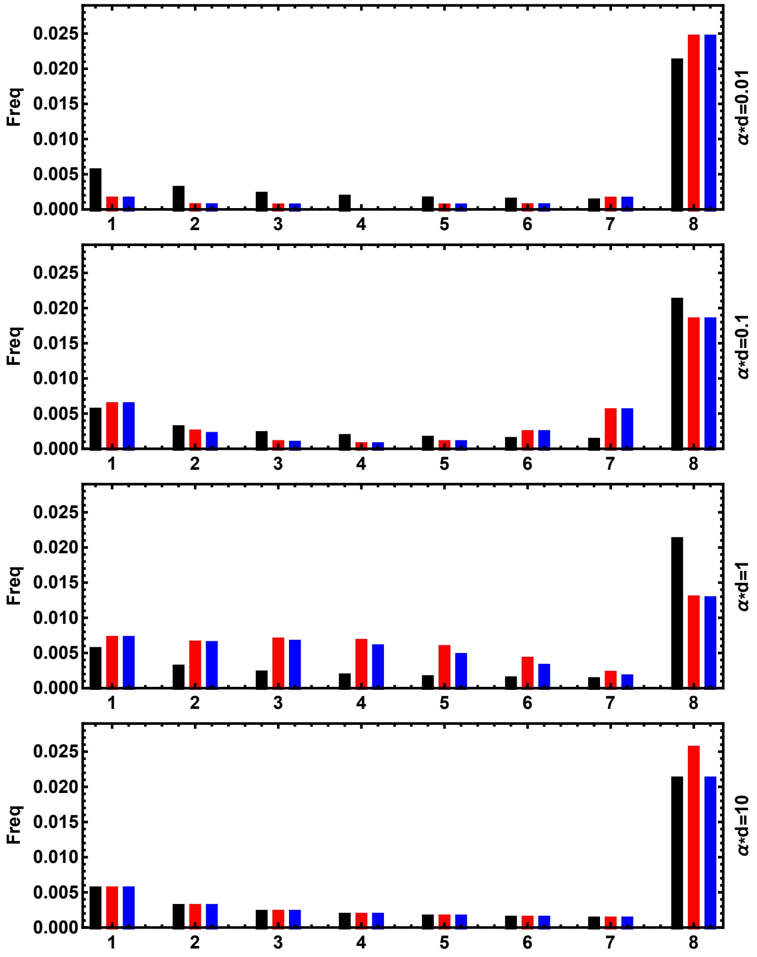

Fig. A4

Pairwise diversity after the selective sweep. Predictions from a sample of two lineages are in gray. Model 1 predictions are in red. Model 2 predictions are in blue. Our original model predictions are in black. Average of simulated data points ± 3 standard error are shown in black. In the upper data set $D = 0.026$, and in the lower data set $D = 0.014$. In both, the remaining parameters are $\theta = 0.002$, $N = 5\,000$, $s = 0.1$, and $n = 50$.

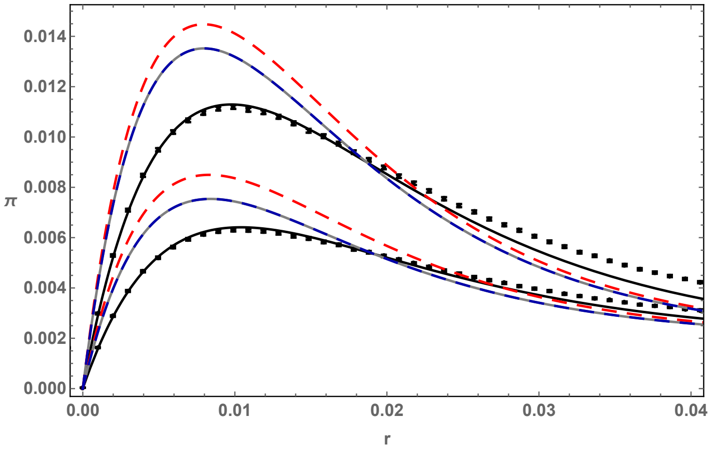

Fig. A5

`VolcanoFinder` optimum model: choice of D Results from the `VolcanoFinder` scan of the average data set described in the supp. Text S1.5. The left column shows the optimum sweep model (inferred strength parameter $\hat{\alpha} = 3.4 \times 10^{-5}$) given the true $D = D_{sim} = 0.026$ used in the simulation. The right column shows the best-fitting model found by the method with inferred parameters $\hat{D} = 0.02$ and $\hat{\alpha} = 3.4 \times 10^{-5}$. The top panels show the average heterozygosity along the sweep region with distance scaled by the true sweep strength αd (gray) as well as the expected diversity predicted under the given model (blue dashed). The remaining rows show the theoretical SFS of the the optimum model (light gray) at increasing distances $\hat{\alpha} d = 0.01, 0.1, 0.5, 1, 2, 3, 8$ from the sweep center and compares this to the observed SFS in a 100-bp window centered at that position averaged over 50 simulations (dark gray). The label on each panel lists the chosen value of scaled distance $\hat{\alpha} d$ from the optimum model as well as the corresponding *true* value of αd determined by the simulation parameters.

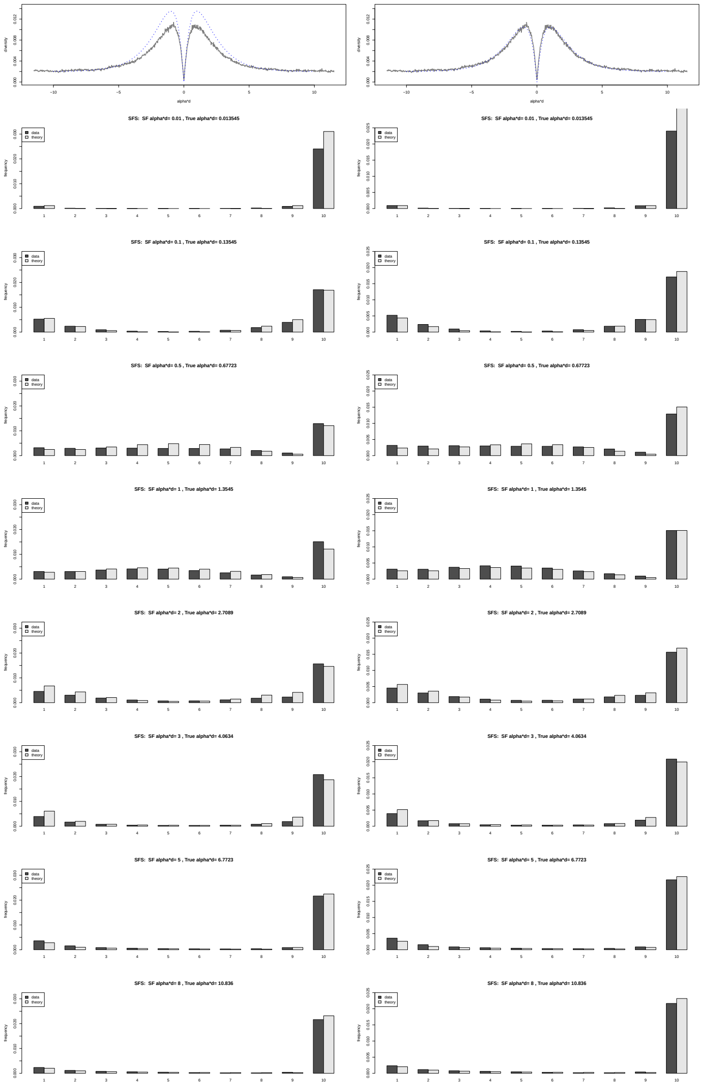

Fig. A6

`SweepFinder` detects the introgression sweep valley. The left column shows the best-fitting model for `SweepFinder`. The `VolcanoFinder` results in the right column as well as the description of the panels are the same as in Fig. A5.

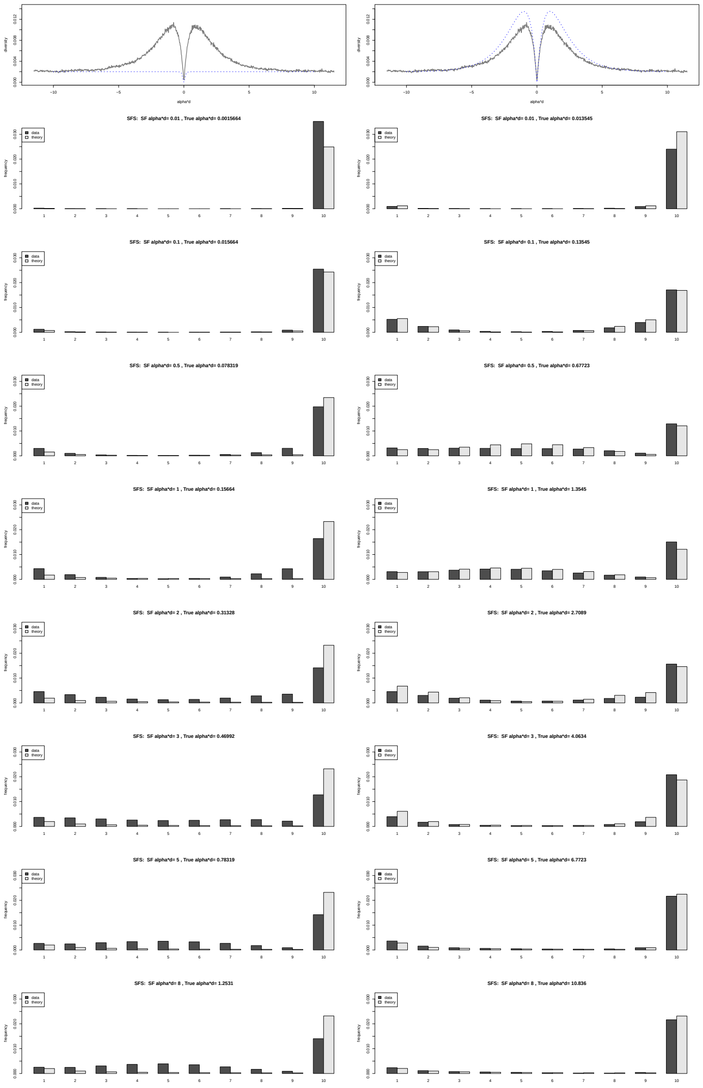

References

1. Kim Y, Stephan W. Detecting a local signature of genetic hitchhiking along a recombining chromosome. Genetics. 2002;160(2):765–777.

2. Stephan W, Wiehe TH, Lenz MW. The effect of strongly selected substitutions on neutral polymorphism: analytical results based on diffusion theory. Theoretical Population Biology. 1992;41(2):237–254.

3. Otto SP, Barton NH. The evolution of recombination: removing the limits to natural selection. Genetics. 1997;147(2):879–906.

4. Barton NH. The effect of hitchhiking on neutral genealogies. Genet Res. 1998;72:123–133.

5. Durrett R, Schweinsberg J. Approximating selective sweeps. Theoretical Population Biology. 2004;66(2):129–138.

6. Uecker H, Hermisson J. On the fixation process of a beneficial mutation in a variable environment. Genetics. 2011;188:915–930.

7. Hermisson J, Pennings PS. Soft sweeps: molecular population genetics of adaptation from standing genetic variation. Genetics. 2005;169(4):2335–2352. doi:10.1534/genetics.104.036947.

8. Zeng K, Fu YX, Shi S, Wu CI. Statistical tests for detecting positive selection by utilizing high-frequency variants. Genetics. 2006;174:1431–1439. doi:10.1534/genetics.106.061432.
