# Supplementary material for: VolcanoFinder: Genomic scans for adaptive introgression
Source: PLoS Genet. 2020 Jun 18;16(6):e1008867. doi: 10.1371/journal.pgen.1008867 (PMC7326285; doi:10.1371/journal.pgen.1008867)
Supplement: S2 Appendix — Supplementary material for the power analysis within the context of a large genomic background, organized into the following sections: (1) Adjusting the migration rate and duration during the sweep (2) Coalescent simulations (3) Genome scans (4) Statistical power in non-admixed genomic background (5) Probability of detecting an introgression sweep in an outlier study (6) Inferred parameters of the selection model. (PDF) [file pgen.1008867.s002.pdf]

VolcanoFinder

genomic scans for adaptive introgression

S2 Power Analysis - Large Genome

Derek Setter[1,2,3☯¤], Sylvain Mousset[1☯], Xiaoheng Cheng[4], Rasmus Nielsen[5], Michael DeGiorgio[6‡], Joachim Hermisson[1,2,7‡],

1 Department of Mathematics, University of Vienna, Vienna, Austria
2 Vienna Graduate School of Population Genetics, Vienna, Austria
3 School of Biological Sciences, University of Edinburgh, Edinburgh, United Kingdom
4 Huck Institutes of the Life Sciences, Pennsylvania State University, University Park, PA, USA
5 Departments of Integrative Biology and Statistics, University of California, Berkeley, CA, USA
6 Department of Computer and Electrical Engineering and Computer Science, Florida Atlantic University, Boca Raton, FL, USA
7 Max F. Perutz Laboratories, University of Vienna, Vienna, Austria

☯These authors contributed equally to this work.
‡These authors also contributed equally to this work.
¤Current Address: School of Biological Sciences, University of Edinburgh, Edinburgh, United Kingdom
*Correspondence: joachim.hermisson@univie.ac.at (JH), mdegiorg@fau.edu (MD)

S2 Power Analysis: Large Genome

Text S2.1

Adjusting migration rate and duration of the introgression sweep.

We use the model in Fig. B1, where introgression from a donor species occurs as a short and sudden migration episode from the donor to the recipient species. In order to simulate the sweep of an introgressed selected allele, we adjust the migration parameter m such that the expected frequency p_0 of the introgressed selected allele just after the migration episode leads to the fixation of the selected allele with a high probability $\pi_{\text{fix}} = 0.95$.

Migration rate. Under a diploid additive model the probability of ultimate fixation of a beneficial allele with initial frequency p_0 is given by eq. (5.47) in [1]

$$\pi_{\text{fix}} = \frac{1 - e^{-2Nsp_0}}{1 - e^{-2Ns}}. \tag{S2.1}$$

Isolating p_0 in eq. (S2.1) leads to

$$p_0 = -\frac{1}{2Ns} \ln\left(1 - \left(1 - e^{-2Ns}\right)\pi_{\text{fix}}\right). \tag{S2.2}$$

In a discrete generation model, the backward migration rate m is defined as the probability for a lineage in the recipient population to come from the donor population at the previous generation, we thus have $p_0 = m$ and adjust the migration rate to obtain a desired fixation probability for the selected allele. In a continuous coalescent model where time is scaled in units of $4N$ generations, a comparible result is obtained by seting the migration rate to $m = p_0/\Delta t_{\text{mig}}$ during a time interval Δt_{mig}.

Duration of the sweep phase. We assume weak selection such that $\bar{w} \approx 1$ and the dynamics of the frequency of the beneficial allele is well described by the logistic model [1, eq. (1.27)]. If the time t is scaled in units of $4N$ generations, we have

$$\frac{\mathrm{d}p}{\mathrm{d}t} = 4Nsp(1-p) \Rightarrow t = \frac{1}{4Ns}\left(\ln p - \ln(1-p)\right) + C, \tag{S2.3}$$

where C is an integration constant. The duration of the sweep phase Δt_{sweep} can be computed from the frequencies of the beneficial mutation at the introgression time p_0 and at the end of the sweep $p = 1 - 1/(2N)$:

$$\Delta t_{\text{sweep}} = \frac{1}{4Ns}\left(\ln\left(1 - \frac{1}{2N}\right) - \ln\frac{1}{2N} - \ln p_0 + \ln(1-p_0)\right). \tag{S2.4}$$

Including drift at the end of the sweep would substantially shorten the duration of the sweep, and using eq. (S2.4) enables that the selected went to fixation with a high probability.

Text S2.2

Coalescent simulations.

The coalescent simulations were conducted with the `R` package `coala` [2] as a frontend to the coalescent simulator `msms` [3].

Coalescent simulations involving introgression sweeps.

We used the model described in supp. Fig. B1. We simulated coalescent trees for $n = 40$ lineages sampled from the recipient species and one from the outgroup species. The simulated region comprises 2×10^5 nucleotides, the recombination rate was set to $\rho = 5 \times 10^{-7}$ events per generation per nucleotide and the mutation rate was set to $\mu = 1.25 \times 10^{-7}$ events per generation per nucleotide following previous studies [4]. The speciation time between the outgroup and the ancestor of the donor and recipient species was $T_{sp} = 10$ units of $4N$ generations (8 Mya with $N = 10^4$ and a 20 years generation time). The divergence time between the donor and recipient species was $T_d \in \{1, 2.5, 4, 5.5\}$ ($D/\theta \in \{3, 6, 9, 12\}$, equivalent to 0.8, 2, 3.2, and 4.4 Mya, respectively). Taking into account an average expected time of $2N$ generations for the coalescence of a pair of lineages coming for the donor and recipient species, these values of T_d lead to an average probability for a nucleotide to differ between the donor and the recipient species (average divergence) of $D \in \{0.015, 0.03, 0.045, 0.06\}$. The population sizes of the recipient species, the ancestor of the recipient and donor species, as well as the outgroup species were set to $N = 10^4$ individuals. To mimic a sweep from a single lineage introgressed from the donor species (hard introgression sweep), the population size of the donor species was reduced to a single individual ($N_d = 1$) at the time of the split such that all lineages that trace back into the donor species coalesce almost instantaneously. We also relaxed this assumption, restraining this bottleneck to a tiny time interval (20 generations) after the split from the recipient species and thus allowing the donor species to recover polymorphism before the introgression event, potentially allowing sweeps from different lineages in the recipient species (soft introgression sweeps). Because we do not model an initial selective sweep in the donor population, the nucleotide diversity in the donor population is only affected by the strong bottleneck and is not locally reduced around the selected site.

Introgression from the donor into the recipient species occurs at time $T_i = T_s + \Delta t_{\text{sweep}} + \Delta t_{\text{mig}}$: migration is allowed from the donor species into the recipient species for a short time interval Δt_{mig}. The migration rate m and duration of the migration interval Δt_{mig} are chosen such that the expected frequency of the selected allele in the recipient species at the end of the migration interval leads to the fixation of the selected allele with a high probability π_{fix} as described in Text S2.1. Two selection coefficients were used $2Ns \in \{100, 1000\}$ and $\pi_{\text{fix}} = 0.95$ was achieved by using an identical migration rate in both cases ($m \approx 0.003$) and letting the duration of the migration event last a single generation ($\Delta t_{\text{mig}} = 1/(4N)$ for $2Ns = 1\,000$) or 10 generations ($\Delta t_{\text{mig}} = 10/(4N)$ for $2Ns = 100$). A logistic model for the dynamics of the selected allele as in eq. (S2.3) eventually leads to fixation after some time given by eq. (S2.4). We assume that sampling was done at time $T_s \in \{0, 0.1, 0.25, 0.5\}$ (equivalent to 0, 80, 200, and 400 kya respectively) after the fixation event.

Total number of replicates. Combining four values for the divergence time $T_d \in \{1, 2.5, 4, 5.5\}$, four values for the time since the selective sweep $T_s \in \{0, 0.1, 0.25, 0.5\}$ and two values for the selection coefficient $2Ns \in \{100, 1\,000\}$ for each hard and soft introgression sweeps leads to 64 parameter sets. For each parameter set, we ran 1 000 coalescent simulations involving selection and 10 000 neutral coalescent simulations with the same admixture level (same migration rate at the same

time point). In addition, a neutral non-admixed reference was obtained from another 10 000 coalescent simulations. In total we thus performed 714 000 (650 000 neutral and 64 000 non-neutral) coalescent simulations.

Coalescent simulations involving balancing selection.

We used three different demographic models (Fig. B2) inspired by the models that were formerly used to investigate the statistical power of `BALLET` [5]. The speciation time between the outgroup and the ingroup species was $T_{sp} = 10$ units of $4N$ generations (8 Mya with $N = 10\,000$ and a 20 years generation time). The simulated sequence comprised 2×10^5 nucleotides. Balancing selection involved a selected locus with two alleles (A and a) in the middle of the sequence. We assumed overdominance with the following fitnesses: $w_{aa} = 1$, $w_{Aa} = 1 + hs$ and $w_{AA} = 1 + s$ with $h = 100$ and $s = 0.01$. Balancing selection started at different times $T_s \in \{1.25, 5, 8.75, 12.5, 16.25, 20\}$ (equivalent to 1, 4, 7, 10, 13, and 16 Mya, respectively). We assume the selected allele A reaches the equilibrium frequency $h/(2h - 1)$ as soon as selection starts. For the population growth model, population expanded from $N = 10\,000$ to $N_g = 2N$ at time $T_g = 0.06$ (48 kya). For the bottleneck model, population size was reduced from $N = 10\,000$ to $N_b = 0.055N$ at time $T_b = 0.0375$ (30 kya) before returning to its initial size at time $T_e = 0.0275$ (22 kya).

Total number of replicates. Combining three demographic models and six values for T_s leads to 18 parameter sets. We ran 1 000 coalescent simulations involving selection for each parameter set, and 10 000 neutral coalescent simulations for each demographic model. In addition to these simulations, a single simulation of a 2×10^7 nucleotides sequence was performed to generate a genomic background reference for each demographic model. In total we thus performed 48 003 (30 000 neutral, 18 000 non-neutral and 3 neutral background references) coalescent simulations.

Fig. B1

Introgression model. The model comprizes three species. The ancestor of the donor and recipient species diverged from an outgroup species at time T_{sp} in the past. The donor and recipient species diverged at time T_d in the past. Immediately after this speciation event, selection starts (diploid additive model with selection coefficient s) in the donor and recipient species, but the beneficial allele is only present in the donor species, where it is assumed to have already reached fixation. The donor species is bottlenecked to a population size of $N' = N_d$ individuals (N_d is assumed to be very small so that coalescence of lineages in the donor species is immediate). The bottleneck may last until present (enforcing a hard introgression sweep, vertical dashed line) or only occur for a very short period after which the donor population size is set to $N' = N$, allowing the donor population to recover some polymorphism before the introgression occurs (soft introgression sweep). At time T_i in the past, migration occurs for a small amount of time (one to ten generations) from the donor to the recipient species. At this time point the beneficial allele is introgressed in the recipient population. The migration rate m is set such that the fixation probability of the beneficial allele in the recipient population is 0.95 given its expected initial frequency in the recipient population at time T_i. The selected allele reaches fixation in the recipient species at time T_s.

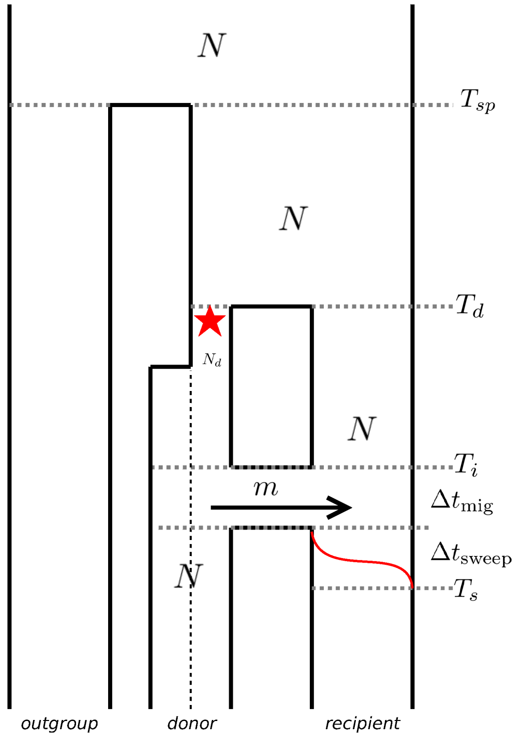

Fig. B2

Balancing selection models. The model comprizes two species, the outgroup and the ingroup species that diverged at time T_{sp} in the past, when the ancestor species (size N_a) splitted into two species of equal sizes $N = N_a$. Balancing selection starts at time T_s either in the ingroup species ($T_s < T_{sp}$) or in the ancestor species ($T_{sp} < T_s$). **A** Constant population size model. **B** Population growth model: the size of the ingroup species expanded to N_g at time T_g. **C** Bottleneck model: the size of the ingroup species was reduced to N_b between times T_b and T_e.

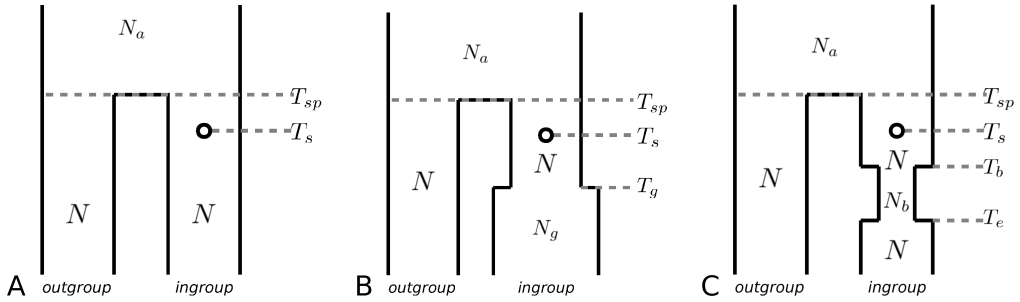

Text S2.3

Genome scans.

Genome scans for introgression sweeps. We compared the performance of three model-based composite likelihood methods: `BALLET` [5], `SweepFinder2` [4] and `VolcanoFinder`. For each method, two cases were considered for the reference genomic background. We considered either a non-admixed reference inferred from 10 000 neutral coalescent simulations without introgression or an admixed reference inferred from 10 000 neutral coalescent simulations involving the same level of admixture as the non-neutral coalescent (same migration rate at the same time point) as described in Text S2.2 and Fig. B1.
`BALLET`: the $T2$ test of `BALLET` was used with a sliding window size of 21 informative sites (half-window size of 10 sites).
`SweepFinder2`: the composite likelihoods were computed on a grid of 800 locations for the selected site (250 nucleotides spacing, four times denser than that used in the original article on `SweepFinder2`).
`VolcanoFinder`: the model 1 was used to compute the composite likelihoods on a grid of 800 locations for the selected site and 13 values for the divergence parameter $D \in \{0.005, 0.01, 0.015, \ldots, 0.065\}$, encompassing the full range of D in the simulations.

Genome scans for balancing selection. We compared the performance of `BALLET` and `VolcanoFinder` under three demographic scenarios (Fig. B2) inspired by the original `BALLET` article [5]. For each method, the genomic background reference was inferred from a single coalescent simulation of a 2×10^7 nucleotides under the same demographic scenario.
`BALLET`: the $T2$ test of `BALLET` [5] was used with a sliding window size of 21 informative sites (half-window size of 10 informative sites).
`VolcanoFinder`: the model 1 was used to compute the composite likelihoods on a grid of 500 locations for the selected site and 13 values for the divergence parameter $D \in \{0.001, \ldots, 0.013\}$, enabling to encompass the full range of starting time for the selection T_s in the simulations.

Power analysis. For all methods, the maximum LR value in a simulated sequence of 200kb was used as a test statistics.

Rejection rates. The rejection rate of a method for a given false positive rate FPR (up to 0.05) was estimated as the proportion of the non-neutral simulations (among 1 000 non-neutral replicates) leading to a test statistics exceeding the $(1 - FPR)$ quantile of the null distribution (estimated from 10 000 neutral replicates). Because introgression sweeps with a low selection coefficient ($2Ns = 100$) mainly altered the site frequency spectrum within 10 kb from the selected site (see Fig. B4 and Fig. B5), we also computed rejection rates based on the maximum LR value in the central 20 kb in this case.

Probability of detection of an introgression sweep in a genome scan. Whole genome scan usually look for outliers among genome-wide data and selection at a locus is detected if the LR value at this locus ranks among the genome-wide highest peaks. We used the following procedure to mimic such a study: the genome-wide null-distributions of the LR values were obtained from the reference 10 000 neutral replicates of 200 kb (leading to 2 Gb genomes). For each neutral replicate 800 LR values were retained (all values for `VolcanoFinder` and `SweepFinder2` and the highest LR value in 800 non-overlapping window of 250 nt for `BALLET`) leading to 8×10^6 LR values for the whole genome. The

probability to detect an introgression sweep in a genome scan considering a set of top-X candidates was computed as the proportion of the non-neutral replicates (estimated from 1 000 replicates) leading to a maximum LR value (in a single replicate) higher than the Xth genome-wide neutral highest LR peak.

Text S2.4

Statistical power: non-admixed genomic background

In the limiting case of a non-admixed genomic background, `VolcanoFinder` clearly outperforms the other methods (Fig. B3 and Fig. B6). It detects both hard and soft introgression sweeps with strong or moderate selection strength with a probability close to 1 even in very small sets of outliers as long as the divergence from the donor species is large enough ($T_d \geqslant 2.5$, *i.e.*, $D \geqslant 6\theta$). In contrast to classical sweeps, even older introgression events are detected with high power (up to $T_s = 0.5$). The relative performances of `BALLET` and `SweepFinder2` depend on the age of the sweep. For highly diverged species ($T_d \geqslant 4$, $D \geqslant 9\theta$), `SweepFinder2` loses power faster than `BALLET` as the time since the selective sweep increases, because it is sensitive to the valley of expected heterozygosity induced by the selective sweep. This also explains the large reduction in power of `SweepFinder2` for soft sweeps. The better performance of `VolcanoFinder` in detecting introgression sweeps in smaller sets of outliers relies on its higher rejection rates for low false positive rate, see Fig. B6 (the lowest false positive rate on our ROC curves is 0.1%).

For some parameter sets, the power (or detection probability) of the tests exceeds the 95% probability that adaptive introgression occurs in the simulations. This is because the tests really detect local introgression in this setting, as described above. Even in the 5% of simulations where the adaptive allele is eventually lost, there may still be a significant excess of introgressed variation at the focal locus relative to the background. If these variants segregate at intermediate frequencies, then the signal is picked up by scans for adaptive introgression or long-term balancing selection. Note that, when rejection rates exceed 95%, higher rejection rates are observed for weak selection ($2Ns = 100$) than for strong selection ($2Ns = 1\,000$), consistent with the 10 fold higher admixture level needed in the weak selection case to achieve a 95% probability for an introgression sweep to occur.

Fig. B3

Detection probability of an introgression sweep (non admixed background)
Probability of an introgression sweep event to be detected in a genome-scan analysis using `VolcanoFinder` (blue), `BALLET` (brown) and `SweepFinder2` (green). The x-axis represents the number of false-positive peaks from the neutral data which score higher than the true-positive signal.The donor species diverged from the recipient species at (top to bottom) $T_d = 1,\ 2.5,\ 4,\ 5.5$ (*i.e.* $D = 3\theta,\ 6\theta,\ 9\theta,\ 12\theta$) and the selective sweep ended (from left to right) $T_s = 0,\ 0.1,\ 0.25,\ 0.5$ units of $4N$ generations in the past. Solid lines: no polymorphism in the donor species (hard introgression sweep). Dashed lines: polymorphism exists in the donor species (possible soft introgression sweep). Dark colour: $2Ns = 1\,000$; light colour: $2Ns = 100$. Analyses involved a non-admixed neutral genomic background as a reference.

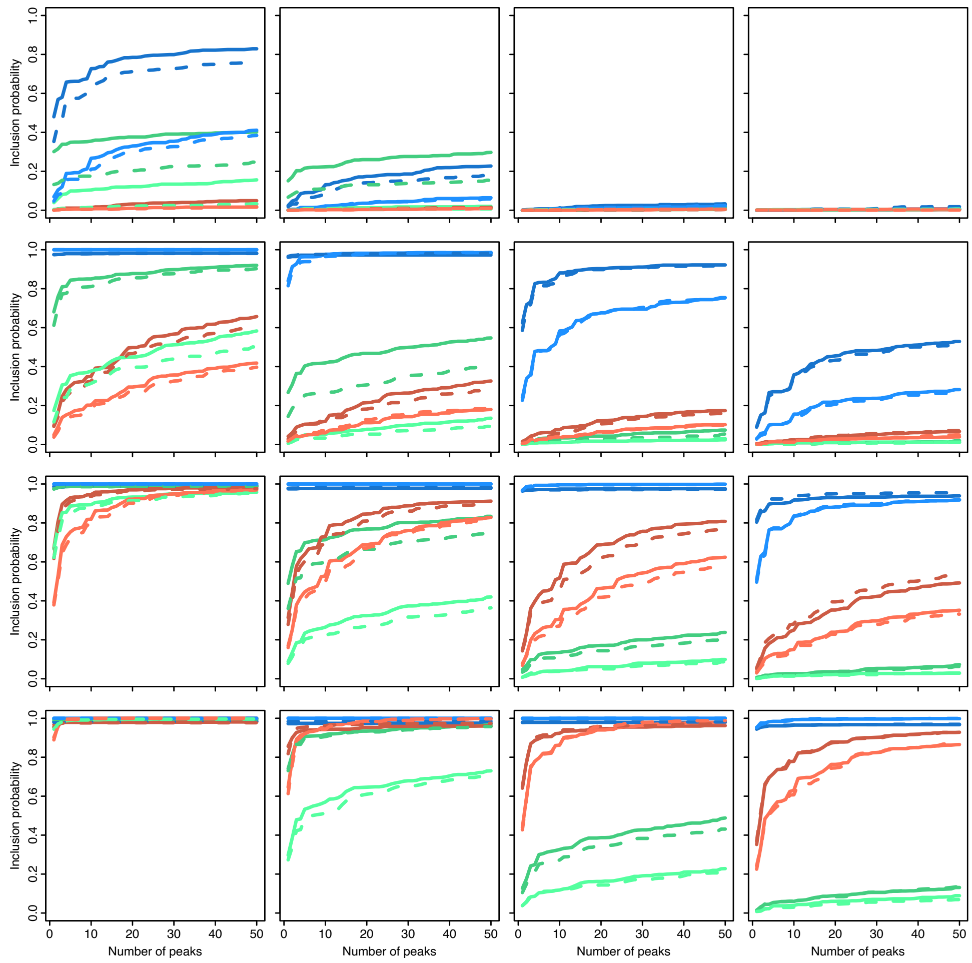

Fig. B4

Volcano patterns caused by a hard introgression sweep. Average nucleotide diversity (Tajima's $\hat{\theta}_\pi$, [6]) in non-overlapping windows of 400 nucleotides in the simulated 200 kb alignments involving a hard introgression sweep. The selection strength is $2Ns = 100$ (left) or $2Ns = 1\,000$ (right). The age of the split between the donor and recipient populations is (from top to bottom) $T_d = 1$, 2.5, 4, 5.5 units of $4N$ generations ($D/\theta = 3$, 6, 9, 12). The ending time of the selective sweep is $T_s = 0$ (red), $T_s = 0.1$ (green), $T_s = 0.25$ (blue), $T_s = 0.5$ (black) units of $4N$ generations before sampling. The coloured horizontal lines indicate the background polymorphism level in all cases. For the lowest selection strength only the central 40 kb region is shown.

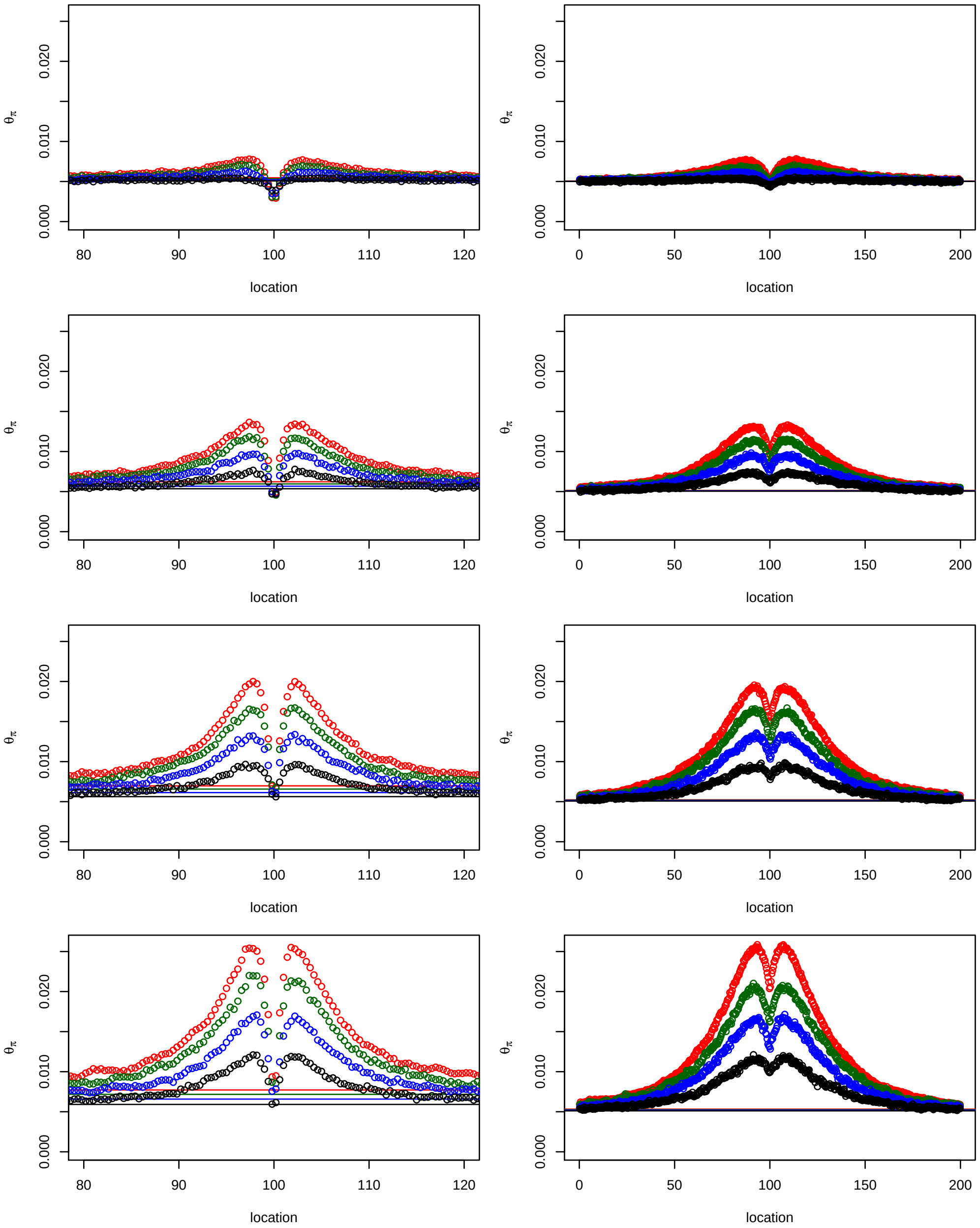

Fig. B5

Volcano patterns caused by a soft introgression sweep. Average nucleotide diversity (Tajima's $\hat{\theta}_\pi$ [6]) in non-overlapping windows of 400 nucleotides in the simulated 200 kb alignments involving a soft introgression sweep. The selection strength is $2Ns = 100$ (left) or $2Ns = 1\,000$ (right). The age of the split between the donor and recipient populations is (from top to bottom) $T_d = 1$, 2.5, 4, 5.5 units of $4N$ generations ($D/\theta = 3$, 6, 9, 12). The ending time of the selective sweep is $T_s = 0$ (red), $T_s = 0.1$ (green), $T_s = 0.25$ (blue), $T_s = 0.5$ (black) units of $4N$ generations before sampling. The coloured horizontal lines indicate the background polymorphism level in all cases. For the lowest selection strength only the central 40 kb region is shown.

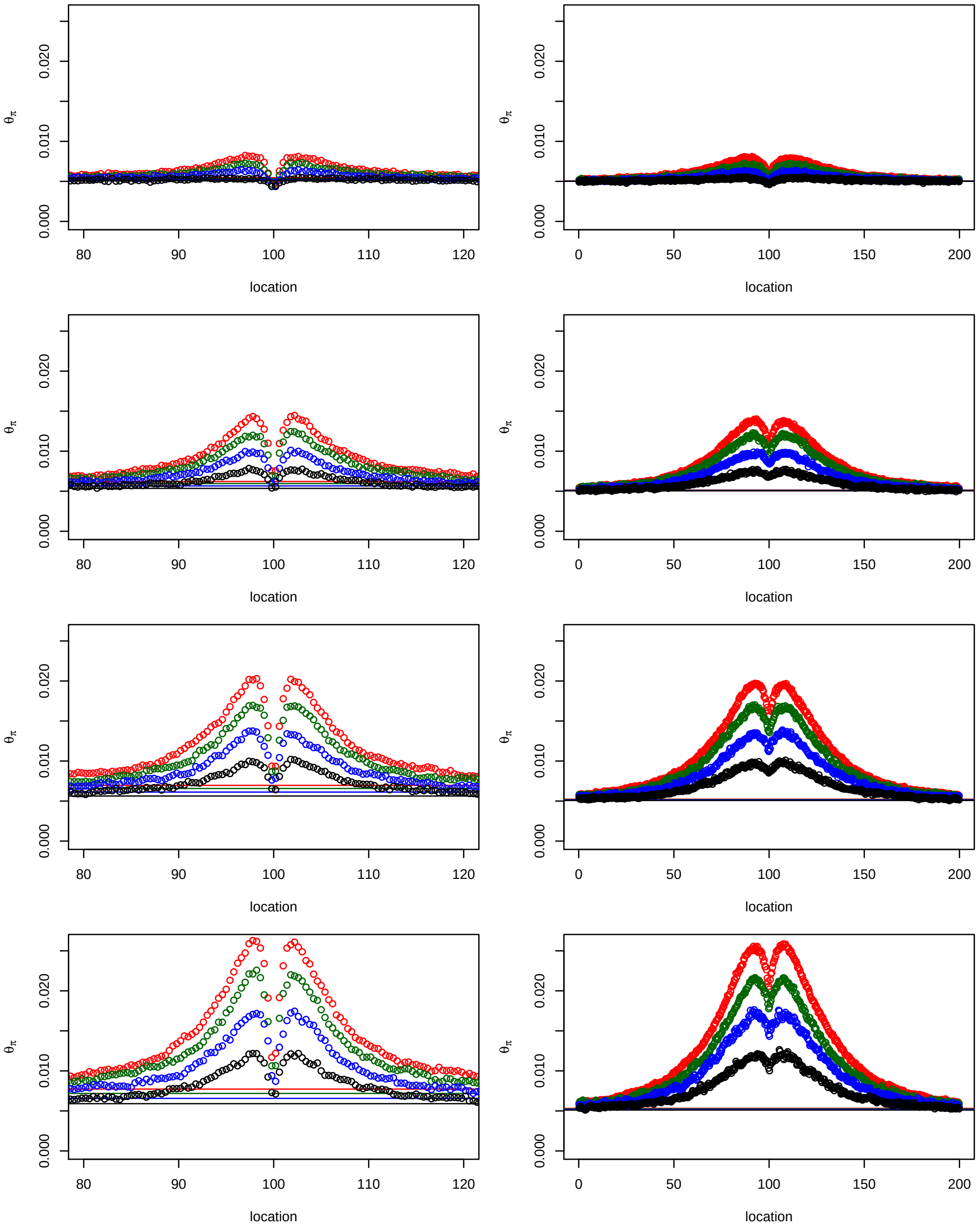

Text S2.5

Probability of detection of an introgression sweep in an outlier study

Analyses of test power typically display the true positive rate against the false positive rate in a so-called ROC curve. In the current study, ROC curves of this type are provided in the supporting information (Fig. B6 and Fig. B7). However, when a test is applied to actual data, the problem is slightly different. An introgression sweep event is identified in a genome-wide scan if the CLR values in the region involved in the introgression sweep rank among the highest genome-wide candidate peaks. We therefore define a detection probability given a number X of candidate peaks considered as the probability that the focal locus ranks among the top X CLR value peaks. The large number of neutral replicates we used for the power analysis is comparable to a full genome scan (for each parameter set, we produced 10^4 neutral replicates of 200 kb sequences leading to a 2 Gb alignment and 8×10^6 CLR values) and enabled us to estimate these detection probabilities (Fig. B3 and Fig. 5). Both ways to display test power are related: a high rejection rate for a very low false positive rate guarantees a high-ranking peak in a genome scan. However there are important differences: Rejection rates of ROC curves usually consider the maximum CLR statistics in windows around a focal site and are thus dependent on the (to some extent arbitrary) width of these windows. In contrast, the outlier-peak approach (like a scan of real data) uses the width of observed peaks to account for local linkage and therefore does not depend on a predefined window width.

Admixed Genomic BAckground As mentioned above, the rejection rates in ROC curves depend on the window size that is used to derive the maximum CLR statistics in the neutral reference. Narrower windows lead to smaller samples of CLR values for the null model, and thus to increased rejection rates. As the region showing the introgression sweep signal is ten times wider for strong selection ($2Ns = 1\,000$) than for weak selection ($2Ns = 100$), narrower windows can, in principle, be used for weaker selection. We therefore also computed the rejection rates based on the maximum CLR in regions of different width around the selected site when selection is weak (200 kb for both $2Ns = 1000$ and $2Ns = 100$ in Fig. B7 ; 200 kb for $2Ns = 1\,000$ and 20 kb for $2Ns = 100$ in Fig. B8). As expected, the rejection rates for $2Ns = 100$ increase (Fig. B8): the gain of statistical power is especially noticeable for old introgression sweeps ($T_s \geqslant 0.1$) for which the rejection rates now clearly exceed the false positive rate. However, it does not reach the high values for the case $2Ns = 1\,000$ and a smaller admixture proportion. The effects of a smaller window size are similar for all three methods studied.

This approach with different window widths was also used when contrasting significant and non-significant tests in the distribution of the estimated selection parameters (position of the selected locus, selection strength, and divergence from the donor species) as inferred by `VolcanoFinder`. These results are described in Text S2.6 and Fig. B9 to Fig. B18.

Fig. B6

Performance curves (non admixed background). Rejection rates of `VolcanoFinder` (blue), `BALLET` (brown) and `SweepFinder2` (green) for an introgression sweep event from a donor species that diverged from the recipient species at (top to bottom) $T_d = 1$, 2.5, 4, 5.5 ($D/\theta = 3$, 6, 9, 12) and a selective sweep that ended (from left to right) $T_s = 0$, 0.1, 0.25, 0.5 units of $4N$ generations in the past. Solid lines: no polymorphism in the donor species (hard introgression sweep). Dashed lines: polymorphism exists in the donor species (possible soft sweep). Dark colour: $2Ns = 1\,000$; light colour: $2Ns = 100$. The upper gray line indicates the expected highest rejection rate given the expected proportion of successful selective sweeps in the sample. Lower gray area: the rejection rate does not exceed the false positive rate. For all three methods, the test statistics is the highest LR value in the simulated 200 000 nucleotides alignment. The test statistics is the highest LR value in the simulated sequence of 200 kb for both $2Ns = 1000$ and $2Ns = 1000$. Analyses involved a neutral non-admixed genomic background as a reference.

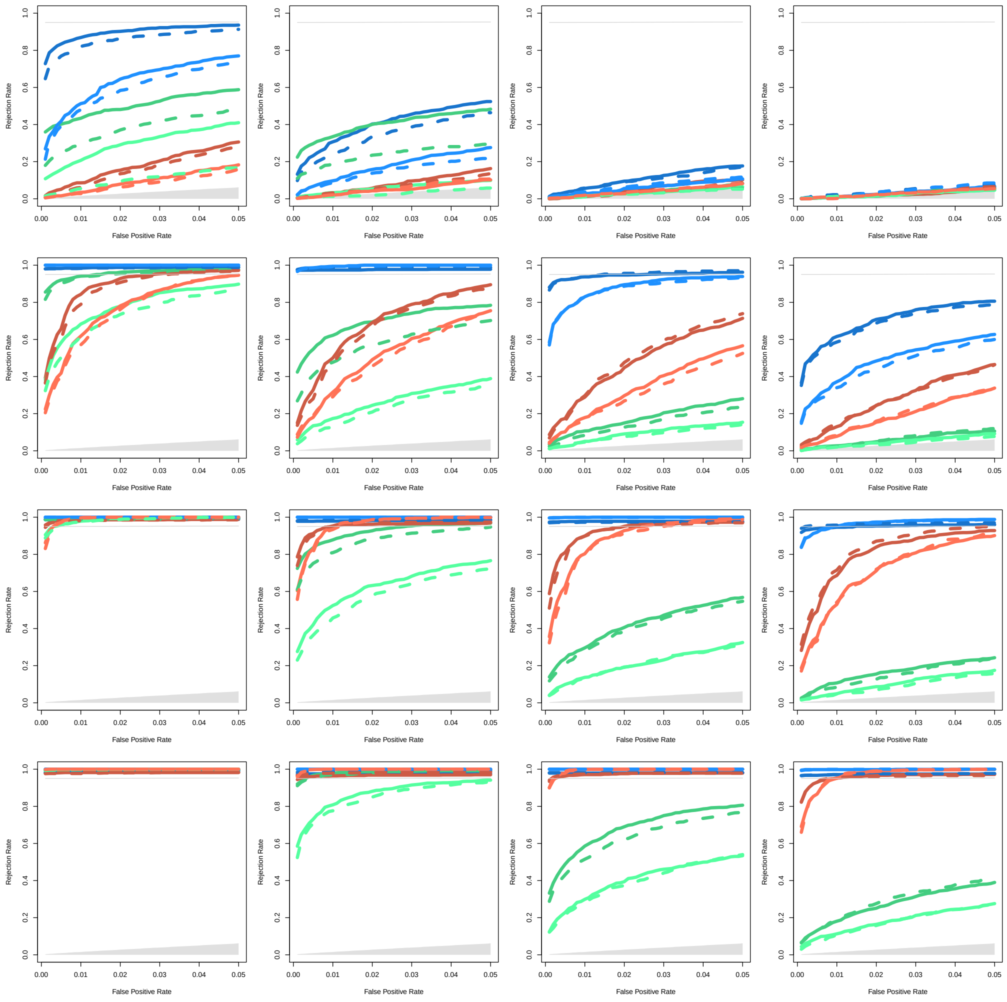

Fig. B7

Performance curves (admixed background). Rejection rates of `VolcanoFinder` (blue), `BALLET` (brown) and `SweepFinder2` (green) for an introgression sweep event from a donor species that diverged from the recipient species at (top to bottom) $T_d = 1, 2.5, 4, 5.5$ ($D/\theta = 3, 6, 9, 12$) and a selective sweep that ended (from left to right) $T_s = 0, 0.1, 0.25, 0.5$ units of $4N$ generations in the past. Solid lines: no polymorphism in the donor species (hard introgression sweep). Dashed lines: polymorphism exists in the donor species (possible soft sweep). Dark colour: $2Ns = 1\,000$; light colour: $2Ns = 100$. The upper gray line indicates the expected highest rejection rate given the expected proportion of successful selective sweeps in the sample. Lower gray area: the rejection rate does not exceed the false positive rate. For all three methods, the test statistics is the highest LR value in the simulated 200 000 nucleotides alignment. The test statistics is the highest LR value in the simulated sequence of 200 kb for both $2Ns = 1000$ and $2Ns = 1000$. Analyses involved a neutral admixed genomic background with the same level of admixture as a reference.

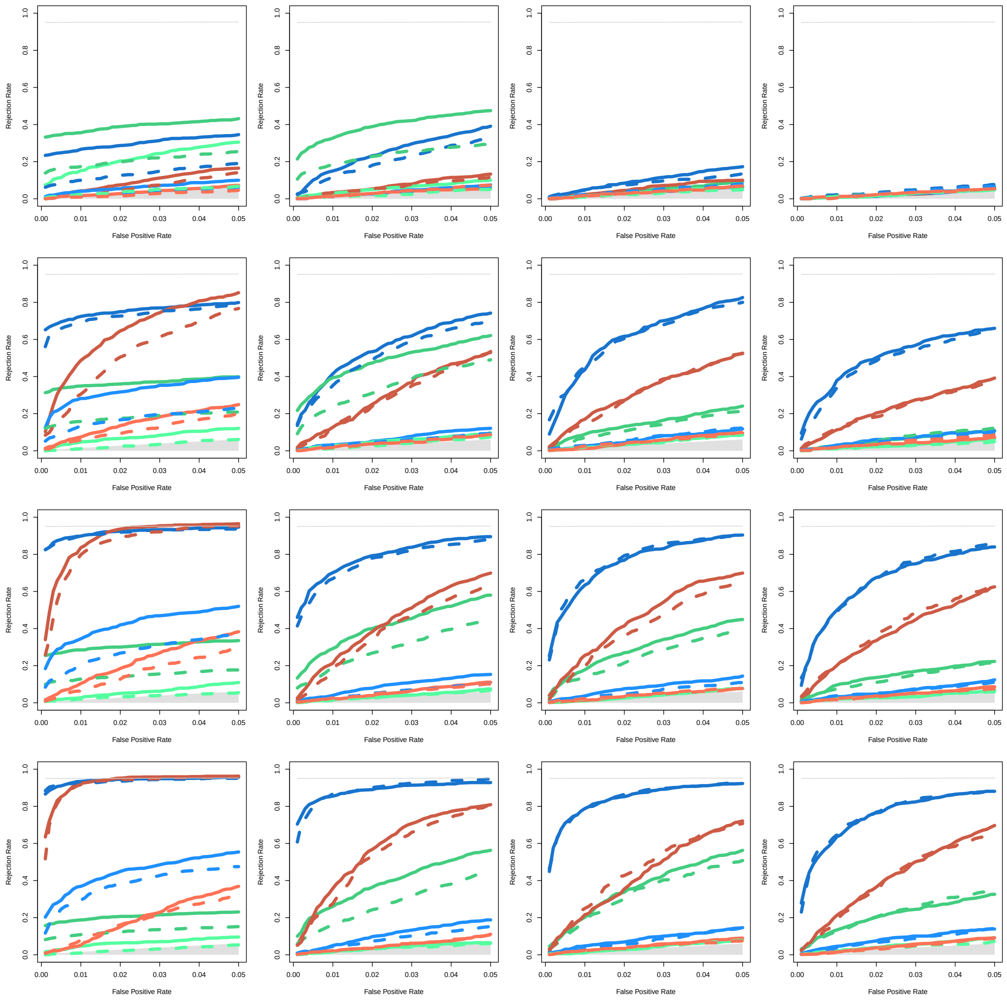

Fig. B8

Performance curves (admixed background and different window sizes). Rejection rates of `VolcanoFinder` (blue), `BALLET` (brown) and `SweepFinder2` (green) for an introgression sweep event from a donor species that diverged from the recipient species at (top to bottom) $T_d = 1$, 2.5, 4, 5.5 ($D/\theta = 3$, 6, 9, 12) and a selective sweep that ended (from left to right) $T_s = 0$, 0.1, 0.25, 0.5 units of $4N$ generations in the past. Solid lines: no polymorphism in the donor species (hard introgression sweep). Dashed lines: polymorphism exists in the donor species (possible soft sweep). Dark colour: $2Ns = 1\,000$; light colour: $2Ns = 100$. The upper gray line indicates the expected highest rejection rate given the expected proportion of succesfull selective sweeps in the sample. Lower gray area: the rejection rate does not exceed the false positive rate. For all three methods, the test statistics is the highest LR value in the simulated 200 000 nucleotides alignment. The test statistics is the highest LR value in the simulated sequence of 200 kb ($2Ns = 1\,000$) or in the central 20 kb ($2Ns = 100$). Analyses involved a neutral admixed genomic background with the same level of admixture as a reference.

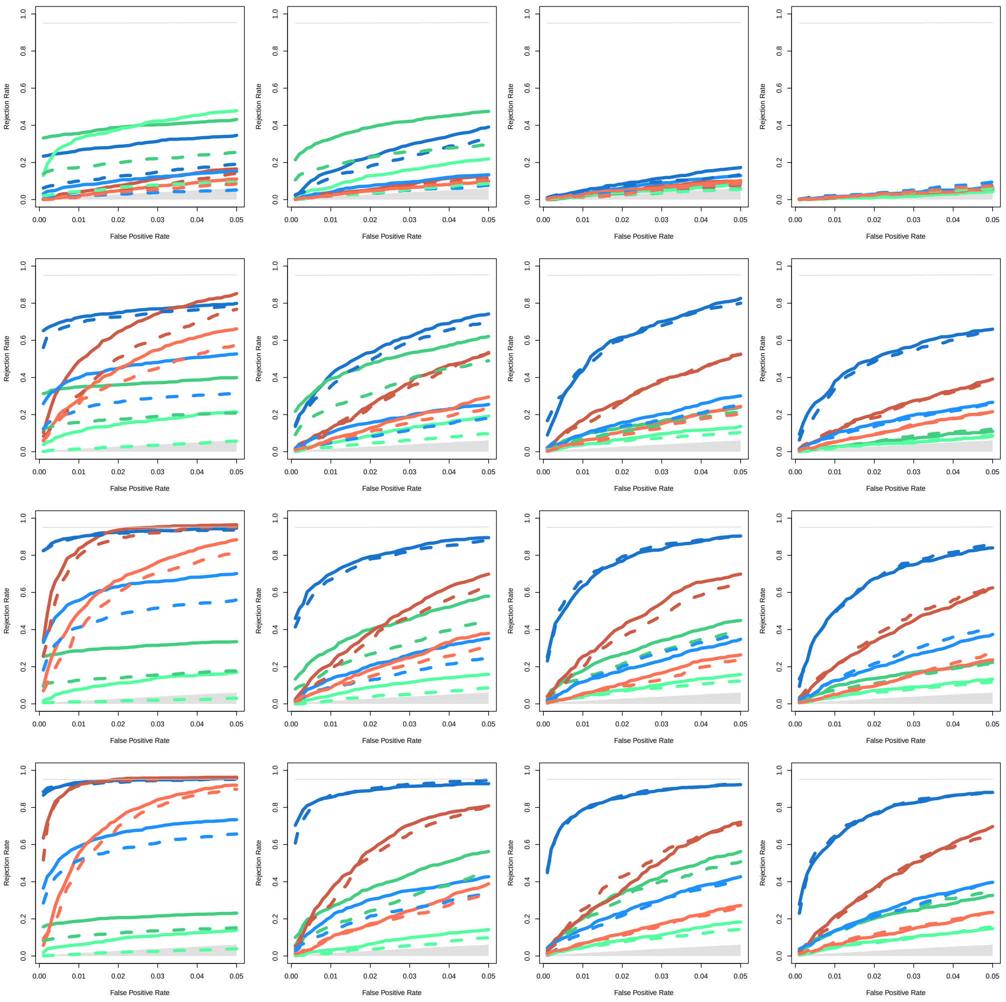

Text S2.6

Inferred parameters of the selection model.

We assessed the accuracy of `VolcanoFinder` to infer the position of the selected site, the compound selection parameter α, and the divergence with the donor species D. For the position of the selected locus, comparisons could be made with the `BALLET` and `SweepFinder` methods. We report the observed distributions of the estimated parameters for each parameter set in the case of a hard introgression sweep. In these distributions we highlight values that lead to a significant CLR test (using an admixed background and a 20 kb window size for $2Ns = 100$ and a 200 kb window size for $2Ns = 1\,000$ as in Fig. B8).

Location of the selected locus as inferred by genome scan methods.

Distributions for the location of the selected site as inferred by the highest CLR value are shown on supp. Fig. B9 to Fig. B14. `VolcanoFinder` and `SweepFinder2` use information from the valley of reduced heterozygosity in the center of the sweep region and locate the target of selection more accurately than `BALLET`, which tries to fit a balancing selection model to the data and thus tends to locate the target of selection in the flanking regions where the polymorphism to divergence ratio is higher. For older introgression sweeps ($T_s \geqslant 0.25$) the accuracy of all methods decreases.

Parameters of the introgression sweep as inferred by `VolcanoFinder`.

The distributions of the scaled divergence parameter $\hat{D}/\theta$ inferred from the location with the highest CLR value are shown on Fig. B15 to Fig. B16. As expected from the analytical analysis, `VolcanoFinder` tends to underestimate D. Unsurprisingly, the mean of the distribution of estimated $\hat{D}$ tends to decrease for older introgression sweeps that typically lead to less pronounced volcano shapes (see Fig. B4). The variance of the distribution of $\hat{D}$ also tends to increase with increasing age of the introgression sweep, probably because our model only considers very recent sweeps.

The distributions of the selection strength inferred parameter $-\log_{10}(\hat{\alpha})$ from the location with the highest CLR value are shown on Fig. B17 to Fig. B18. $-\log_{10}(\alpha)$ seems to be relatively accurately estimated for recent introgression sweeps whereas it might be underestimated in the case of old introgression sweeps that typically lead to narrower volcano shapes (see Fig. B4).

Fig. B9

Location of the maximum LR inferred by `VolcanoFinder` ($2Ns = 1\,000$). Location of the highest LR inferred by `VolcanoFinder` for a hard introgression sweep event with selection coefficient $2Ns = 1\,000$. The donor species diverged from the recipient species at (from top to bottom) $T_d = 1$, 2.5, 4, 5.5 ($D/\theta = 3$, 6, 9, 12) and the selective sweep ended (from left to right) $T_s = 0$, 0.1, 0.25, 0.5 units of $4N$ generations in the past. The coloured and gray parts indicate significant and non-significant test as shown in Fig. B8.

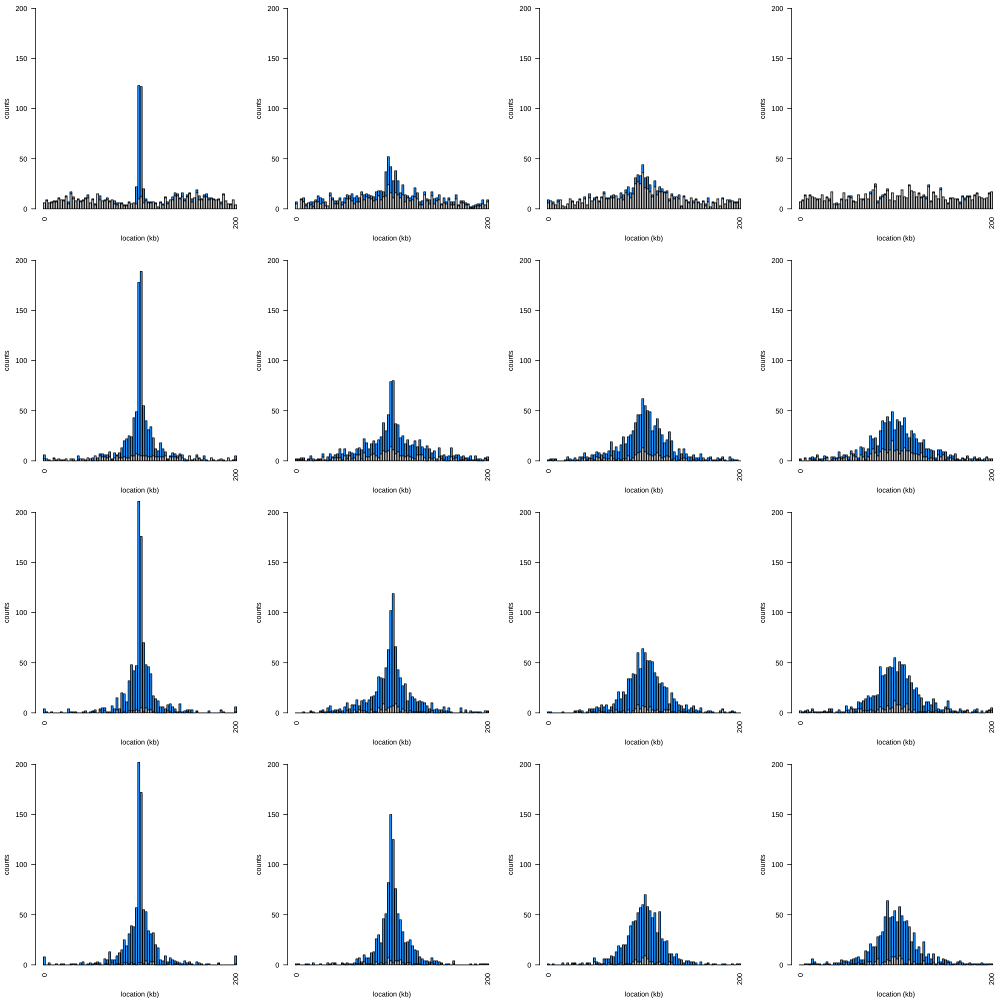

Fig. B10

Location of the maximum LR inferred by `VolcanoFinder` ($2Ns = 100$). Location of the highest LR inferred by `VolcanoFinder` for a hard introgression sweep event with selection coefficient $2Ns = 100$. The donor species diverged from the recipient species at (from top to bottom) $T_d = 1$, 2.5, 4, 5.5 ($D/\theta = 3$, 6, 9, 12) and the selective sweep ended (from left to right) $T_s = 0$, 0.1, 0.25, 0.5 units of $4N$ generations in the past. The coloured and gray parts indicate significant and non-significant test as shown in Fig. B8. Only the central part of the simulated region is shown.

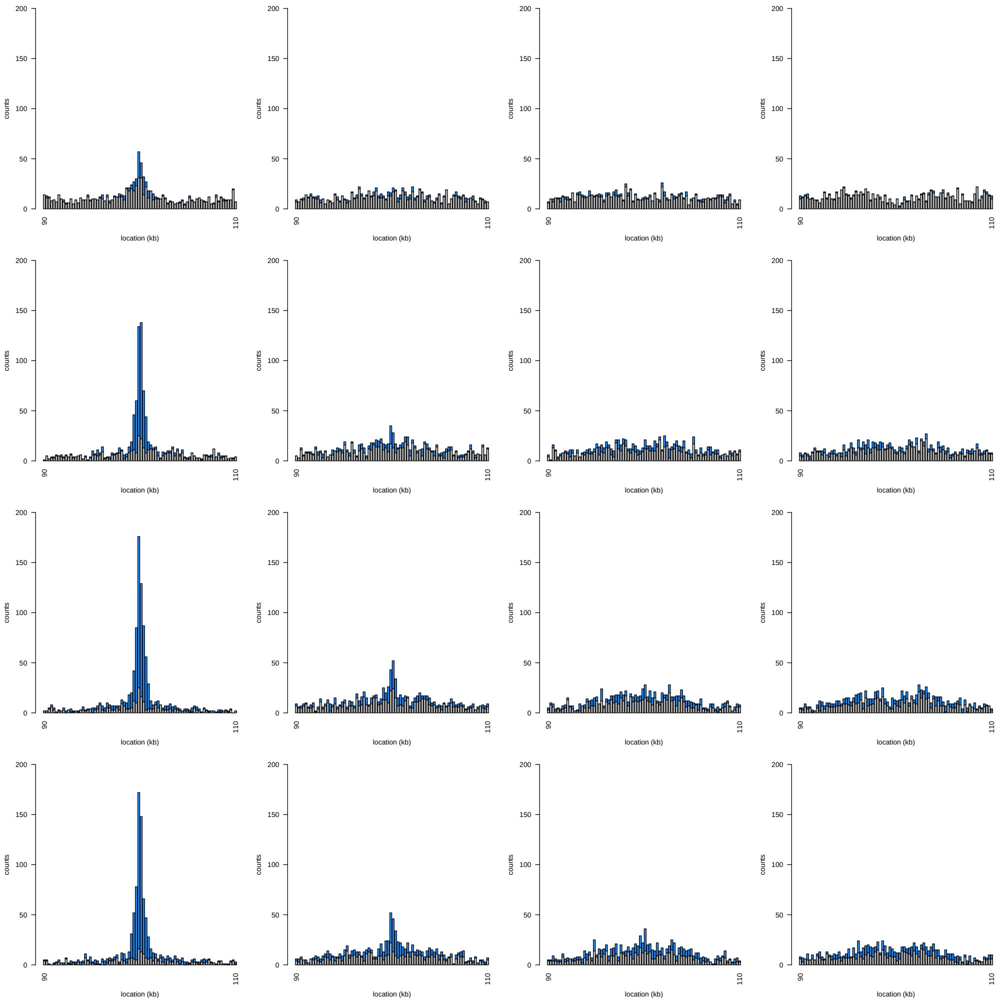

Fig. B11

Location of the maximum LR inferred by `SweepFinder2` ($2Ns = 1\,000$). Location of the highest LR inferred by `SweepFinder2` for a hard introgression sweep event with selection coefficient $2Ns = 1\,000$. The donor species diverged from the recipient species at (from top to bottom) $T_d = 1$, 2.5, 4, 5.5 ($D/\theta = 3$, 6, 9, 12) and the selective sweep ended (from left to right) $T_s = 0$, 0.1, 0.25, 0.5 units of $4N$ generations in the past. The coloured and gray parts indicate significant and non-significant test as shown in Fig. B8.

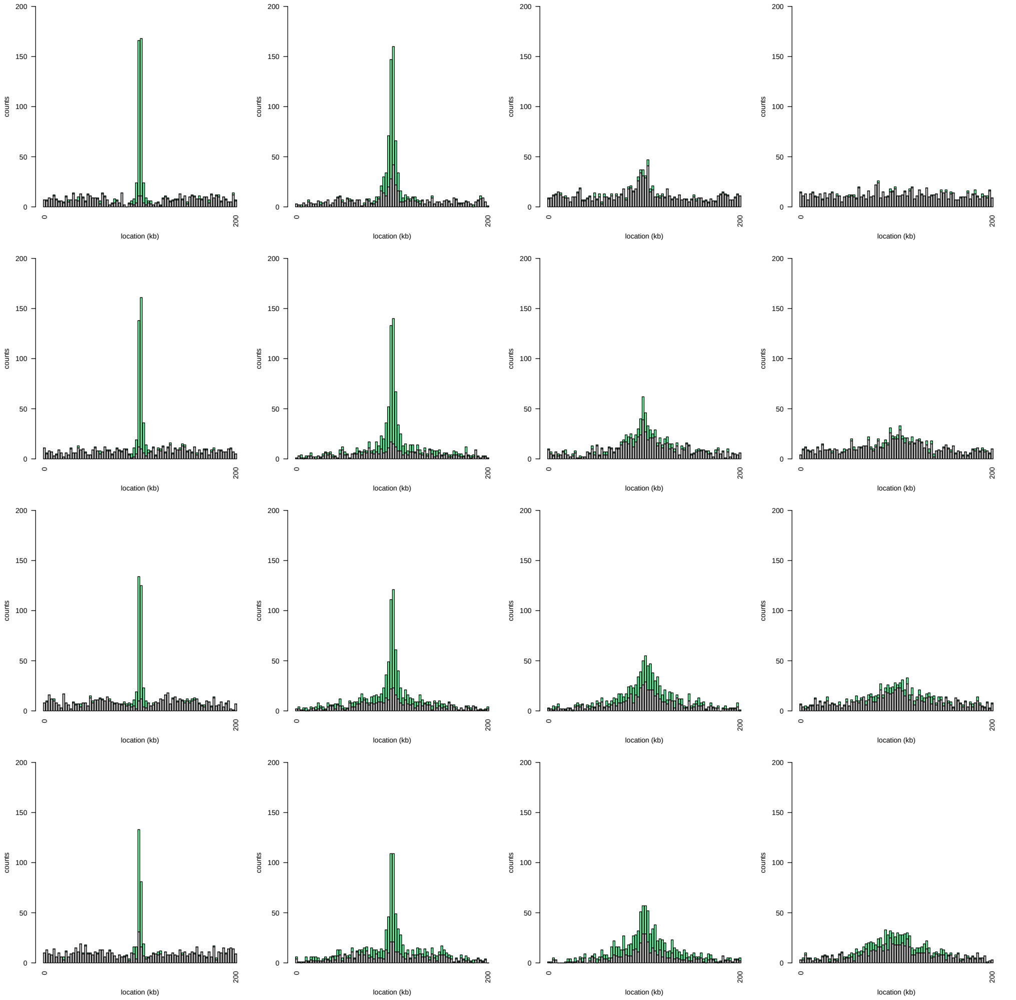

Fig. B12

Location of the maximum LR inferred by `SweepFinder2` ($2Ns = 100$). Location of the highest LR inferred by `SweepFinder2` for a hard introgression sweep event with selection coefficient $2Ns = 100$. The donor species diverged from the recipient species at (from top to bottom) $T_d = 1$, 2.5, 4, 5.5 ($D/\theta = 3$, 6, 9, 12) and the selective sweep ended (from left to right) $T_s = 0$, 0.1, 0.25, 0.5 units of $4N$ generations in the past. The coloured and gray parts indicate significant and non-significant test as shown in Fig. B8. Only the central part of the simulated region is shown.

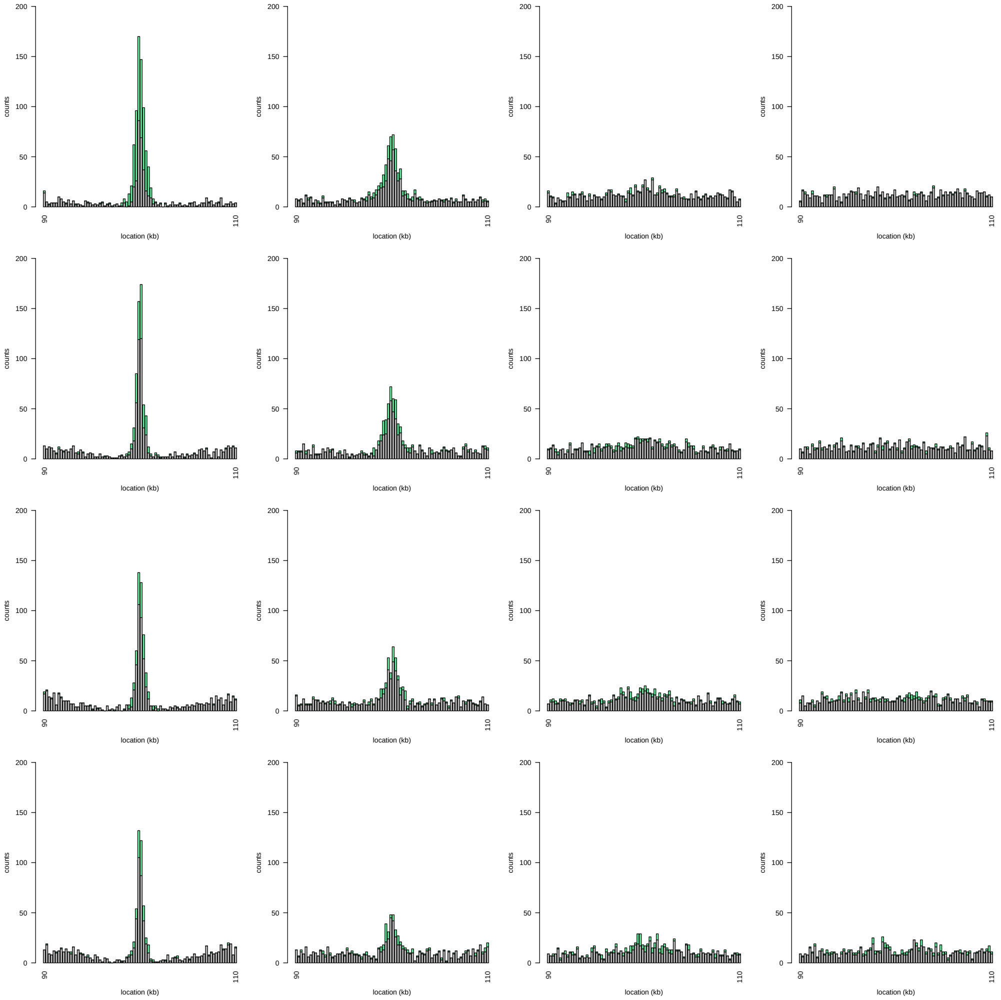

Fig. B13

Location of the maximum LR inferred by `BALLET` ($2Ns = 1\,000$). Location of the highest LR inferred by `BALLET` for a hard introgression sweep event with selection coefficient $2Ns = 1\,000$. The donor species diverged from the recipient species at (from top to bottom) $T_d = 1$, 2.5, 4, 5.5 ($D/\theta = 3$, 6, 9, 12) and the selective sweep ended (from left to right) $T_s = 0$, 0.1, 0.25, 0.5 units of $4N$ generations in the past. The coloured and gray parts indicate significant and non-significant test as shown in Fig. B8.

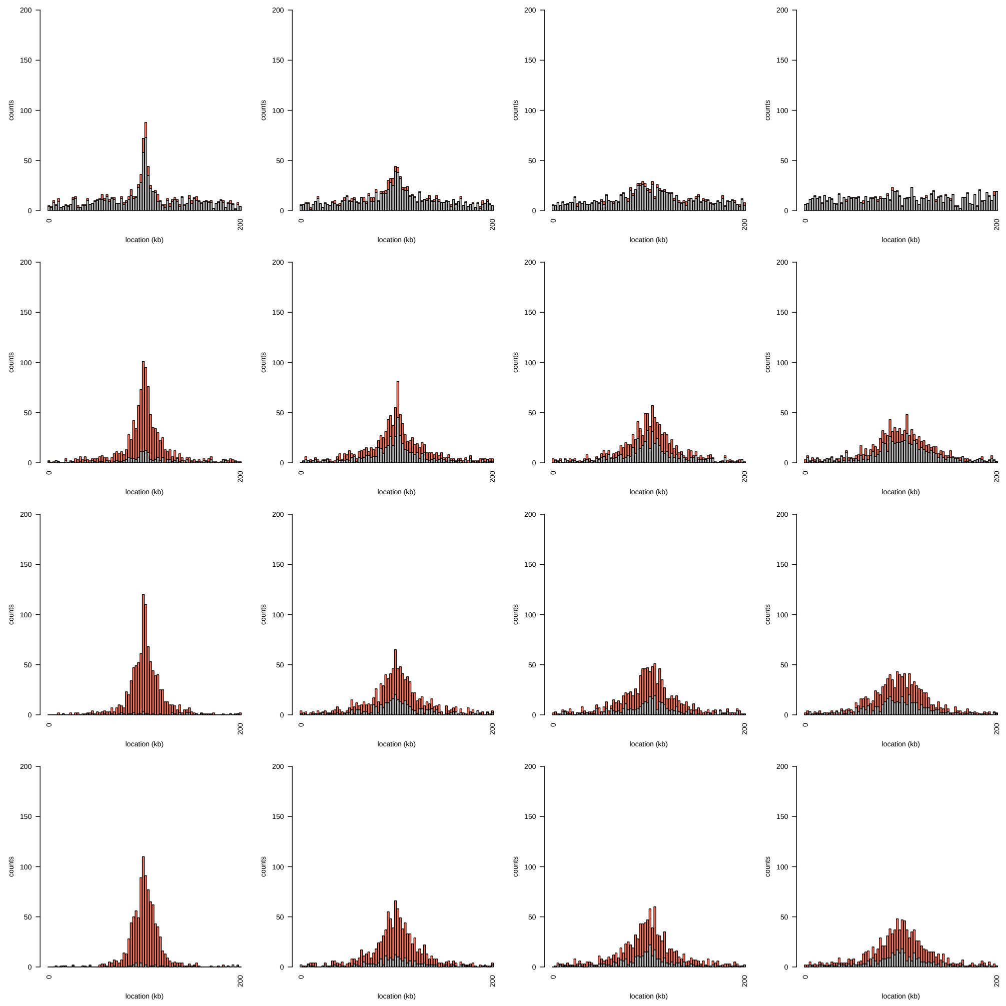

Fig. B14

Location of the maximum LR inferred by `BALLET` **($2Ns = 100$).** Location of the highest LR inferred by `BALLET` for a hard introgression sweep event with selection coefficient $2Ns = 100$. The donor species diverged from the recipient species at (from top to bottom) $T_d = 1$, 2.5, 4, 5.5 ($D/\theta = 3$, 6, 9, 12) and the selective sweep ended (from left to right) $T_s = 0$, 0.1, 0.25, 0.5 units of $4N$ generations in the past. The coloured and gray parts indicate significant and non-significant test as shown in Fig. B8. Only the central part of the simulated region is shown.

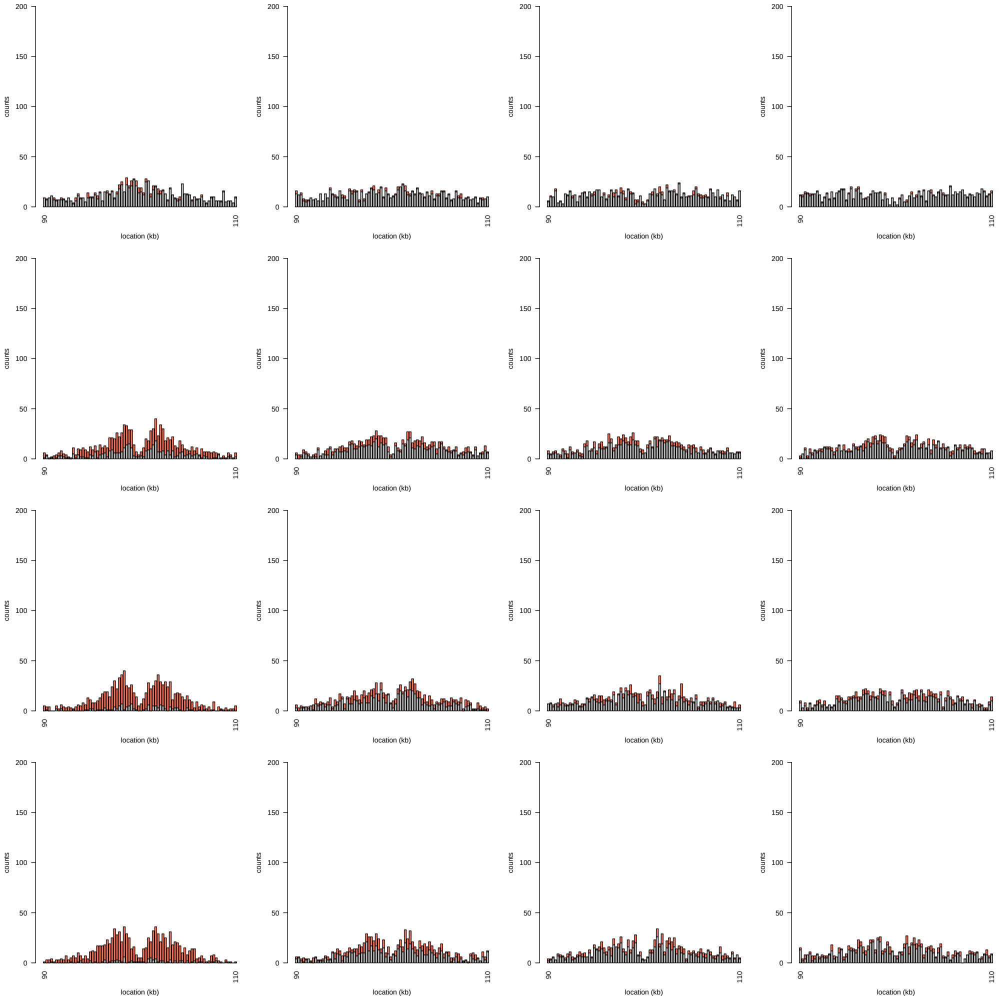

Fig. B15

Divergence from the donor species inferred by `VolcanoFinder` ($2Ns = 1\,000$, hard introgression sweeps). Estimated scaled divergence parameter $\frac{\hat{D}}{\theta}$ at the location with the highest LR inferred by `VolcanoFinder` for a hard introgression sweep event with selection coefficient $2Ns = 1\,000$. The donor species diverged from the recipient species at (from top to bottom) $T_d = 1$, 2.5, 4, 5.5 ($D/\theta = 3$, 6, 9, 12) and the selective sweep ended (from left to right) $T_s = 0$, 0.1, 0.25, 0.5 units of $4N$ generations in the past. The coloured and gray parts indicate significant and non-significant test as shown in Fig. B8. A vertical red line indicates the true value used in the simulations.

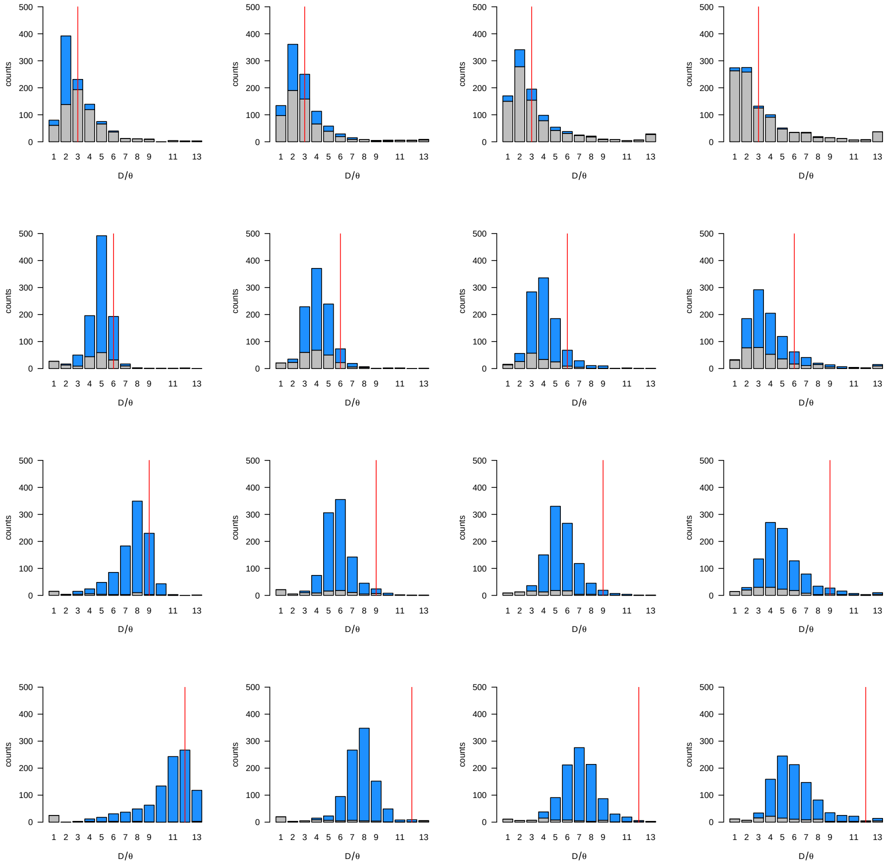

Fig. B16

Divergence from the donor species inferred by `VolcanoFinder` **($2Ns = 100$, hard introgression sweeps).** Estimated scaled divergence parameter $\frac{\hat{D}}{\theta}$ for the location with the highest LR inferred by `VolcanoFinder` for a hard introgression sweep event with selection coefficient $2Ns = 100$. The donor species diverged from the recipient species at (from top to bottom) $T_d = 1$, 2.5, 4, 5.5 ($D/\theta = 3$, 6, 9, 12) and the selective sweep ended (from left to right) $T_s = 0$, 0.1, 0.25, 0.5 units of $4N$ generations in the past. The coloured and gray parts indicate significant and non-significant test as shown in Fig. B8. A vertical red line indicates the true value used in the simulations.

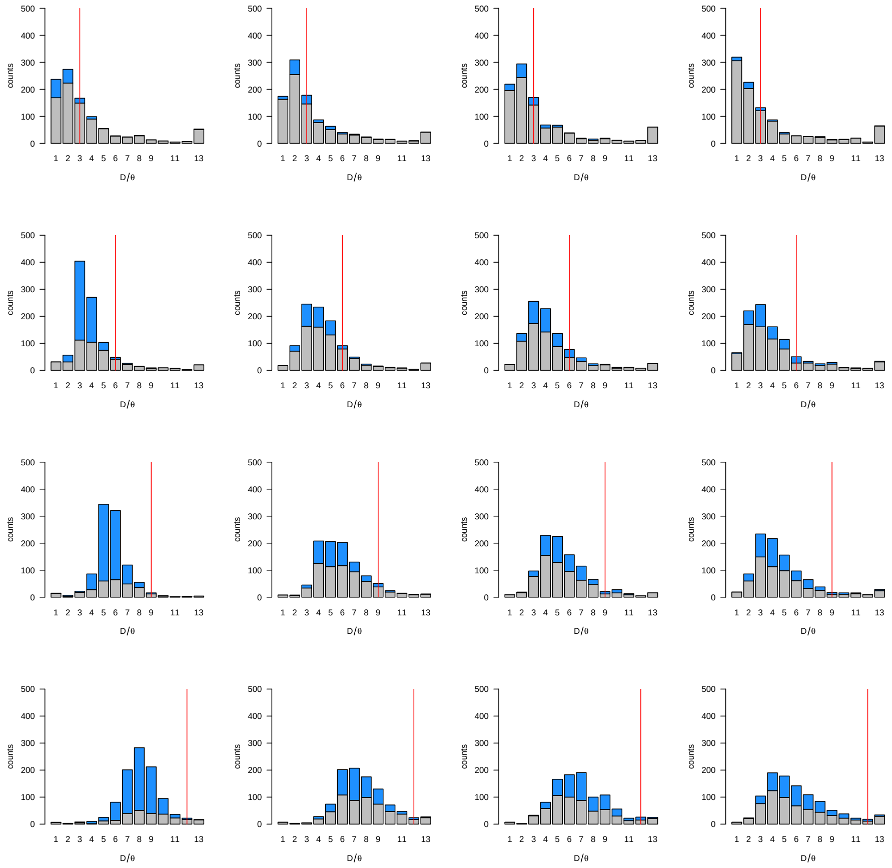

Fig. B17

Selection strength inferred by `VolcanoFinder` ($2Ns = 1\,000$, hard introgression sweeps). Estimated scaled selection parameter $-\log_{10}(\hat{\alpha})$ at the location with the highest LR inferred by `VolcanoFinder` for a hard introgression sweep event with selection coefficient $2Ns = 1\,000$. The donor species diverged from the recipient species at (from top to bottom) $T_d = 1$, 2.5, 4, 5.5 ($D/\theta = 3$, 6, 9, 12) and the selective sweep ended (from left to right) $T_s = 0$, 0.1, 0.25, 0.5 units of $4N$ generations in the past. The coloured and gray parts indicate significant and non-significant test as shown in Fig. B8. A vertical red line indicates the true value used in the simulations.

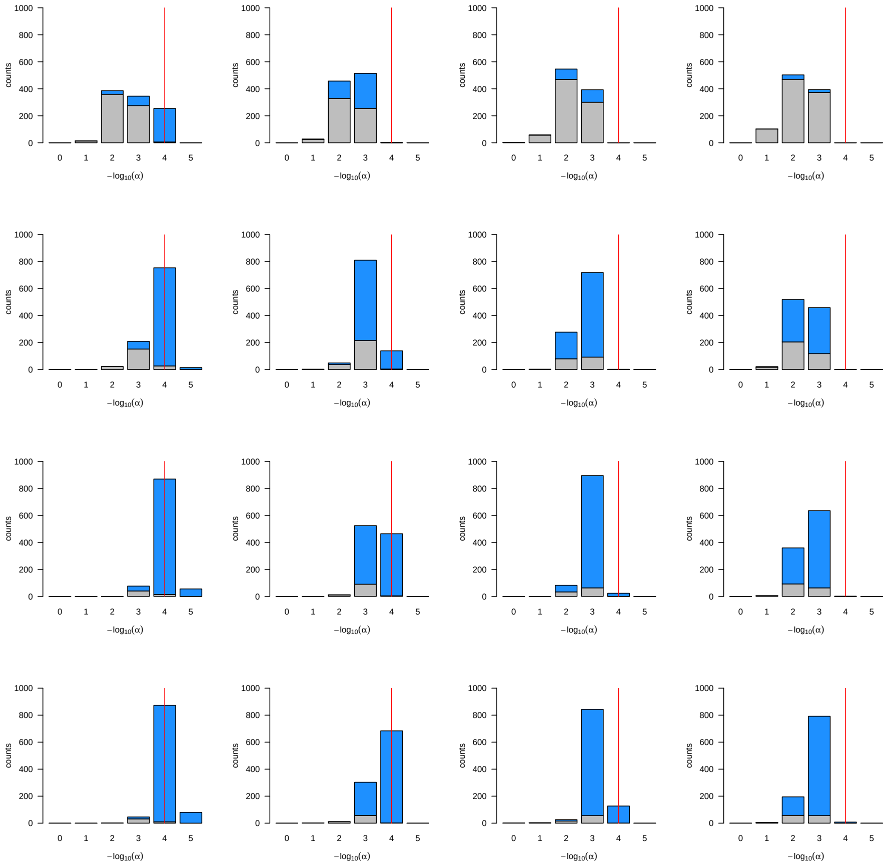

Fig. B18

Selection strength inferred by `VolcanoFinder` ($2Ns = 100$, hard introgression sweeps). Estimated scaled selection parameter $-\log_{10}(\hat{\alpha})$ for the location with the highest LR inferred by `VolcanoFinder` for a hard introgression sweep event with selection coefficient $2Ns = 100$. The donor species diverged from the recipient species at (from top to bottom) $T_d = 1$, 2.5, 4, 5.5 ($D/\theta = 3$, 6, 9, 12) and the selective sweep ended (from left to right) $T_s = 0$, 0.1, 0.25, 0.5 units of $4N$ generations in the past. The coloured and gray parts indicate significant and non-significant test as shown in Fig. B8. A vertical red line indicates the true value used in the simulations.

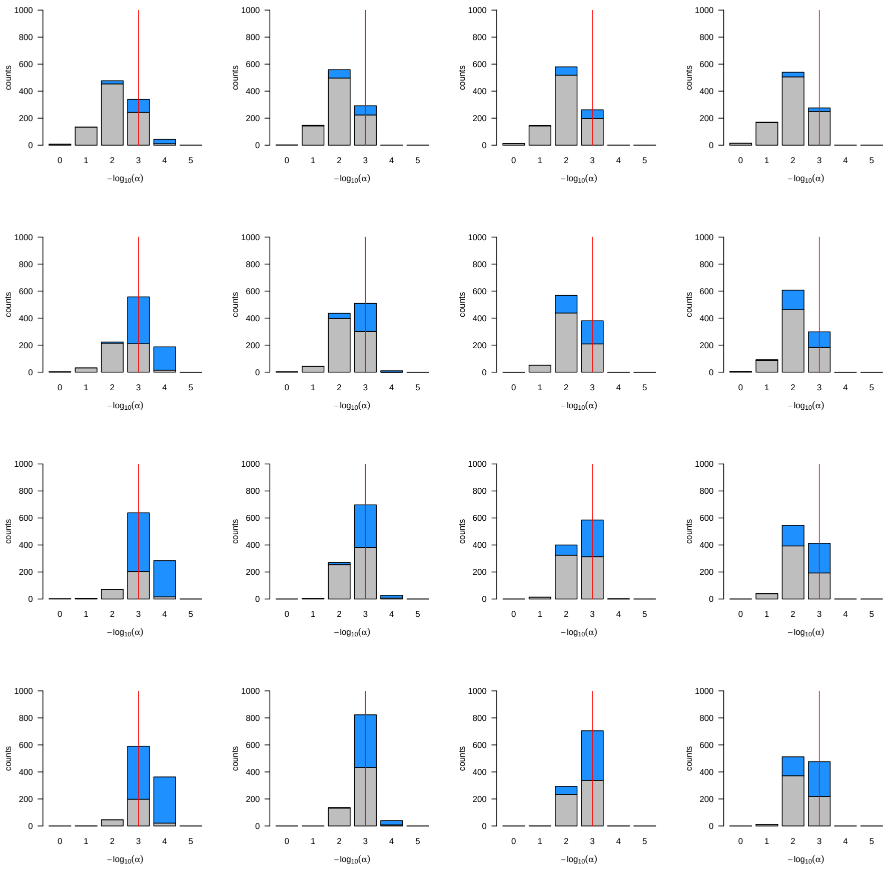

Fig. B19

Performance curves to detect balancing selection. Rejection rates of `BALLET` (solid lines) and `VolcanoFinder` (dashed lines) for different starting time of balancing selection T_s under three demographic scenarios (see Fig. B2). Black: constant population size; blue: population growth; red: bottleneck. Gray area: the rejection rate is not higher than the false positive rate. In all cases, the test statistics is the highest LR value in the simulated 200 kb alignment.

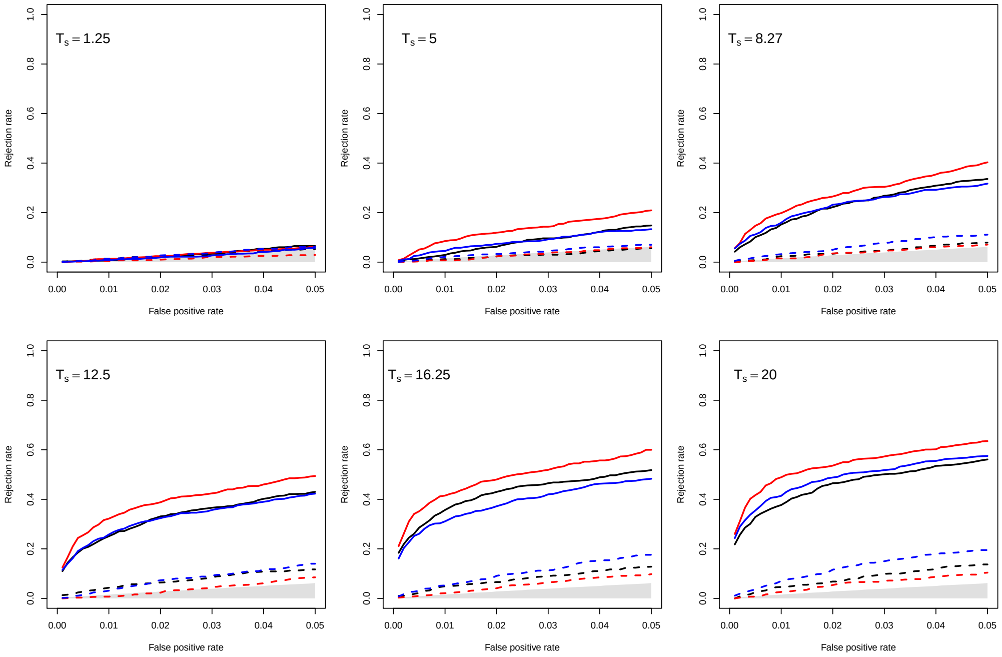

References

1. Ewens WJ. Mathematical population genetics. vol. 9 of Biomathematics. Berlin, Heidelberg, New York: Springer-Verlag; 1979.

2. Staab PR, Metzler D. `coala`: an R framework for coalescent simulation. Bioinformatics (Oxford, England). 2016;32:1903–1904. doi:10.1093/bioinformatics/btw098.

3. Ewing G, Hermisson J. `MSMS`: a coalescent simulation program including recombination, demographic structure and selection at a single locus. Bioinformatics. 2010;26(16):2064–2065. doi:10.1093/bioinformatics/btq322.

4. Huber CD, DeGiorgio M, Hellmann I, Nielsen R. Detecting recent selective sweeps while controlling for mutation rate and background selection. Mol Ecol. 2016;25(1):142–156. doi:10.1111/mec.13351.

5. DeGiorgio M, Lohmueller KE, Nielsen R. A model-based approach for identifying signatures of ancient balancing selection in genetic data. PLoS Genet. 2014;10(8):e1004561. doi:10.1371/journal.pgen.1004561.

6. Tajima F. Statistical method for testing the neutral mutation hypothesis by DNA polymorphism. Genetics. 1989;123(3):585–595.
