# Supplementary material for: VolcanoFinder: Genomic scans for adaptive introgression
Source: PLoS Genet. 2020 Jun 18;16(6):e1008867. doi: 10.1371/journal.pgen.1008867 (PMC7326285; doi:10.1371/journal.pgen.1008867)
Supplement: S3 Appendix — Supplementary material for the power analysis within the context of a large chromosomal region, organized into the following sections: (1) Peak identification and assessing power (2) Power analysis—10 Mb chromosomes (3) Robustness to classic sweeps (4) Robustness to background selection (5) Chimeric chromosomes. (PDF) [file pgen.1008867.s003.pdf]

VolcanoFinder

genomic scans for adaptive introgression

S3 Human Data

Derek Setter[1,2,3☯¤], Sylvain Mousset[1☯], Xiaoheng Cheng[4], Rasmus Nielsen[5], Michael DeGiorgio[6‡], Joachim Hermisson[1,2,7‡],

1 Department of Mathematics, University of Vienna, Vienna, Austria
2 Vienna Graduate School of Population Genetics, Vienna, Austria
3 School of Biological Sciences, University of Edinburgh, Edinburgh, United Kingdom
4 Huck Institutes of the Life Sciences, Pennsylvania State University, University Park, PA, USA
5 Departments of Integrative Biology and Statistics, University of California, Berkeley, CA, USA
6 Department of Computer and Electrical Engineering and Computer Science, Florida Atlantic University, Boca Raton, FL, USA
7 Max F. Perutz Laboratories, University of Vienna, Vienna, Austria

☯These authors contributed equally to this work.
‡These authors also contributed equally to this work.
¤Current Address: School of Biological Sciences, University of Edinburgh, Edinburgh, United Kingdom
*Correspondence: joachim.hermisson@univie.ac.at (JH), mdegiorg@fau.edu (MD)

S3 Power Analysis: Large Chromosome

Text S3.1

Peak identification and assessing power

In this section, we detail the methods we use to determine the power of VolcanoFinder to identify the adaptive introgression allele as an outlier likelihood ratio (LR) value in a genome scan. Suppose we run a VolcanoFinder scan on one such chromosome. If we plot the LR values along the chromosome, then we observe a series of fluctuating scores and can identify a set of taller peaks that stand out from the genomic background. If the method is perfect, then the highest peak will represent the true location of the adaptive introgression allele. When the method is imperfect, in order to find the sweep signal, we must observe a number of false-positive peaks with higher likelihood ratio values. We therefore rank the success of the method by the number of peaks in neutral regions of the genome (false positives) that score higher than the peak for the adaptive introgression allele (true positive). What remains are two very important points: How do we define a true positive signal, and how do we distinguish independent signals in the genome scan?

The sweep-width approach

For each test site, VolcanoFinder reports not only the LR value, but also the divergence value D and sweep strength α for the optimum sweep model. We use α both to distinguish independent peak values in the data and to determine if a peak represents positive detection of the adaptive allele. α is a compound parameter that measures the strength of the sweep relative to the local per-site rate of recombination in the genome (see section: Volcanoes of Diversity). Multiplying α by the distance d in base pairs from the beneficial mutation, we obtain a scaled measure of the effect of a sweep. We have already seen that at a distance of $\alpha d \approx 6$, genetic diversity is only slightly increased above background levels (see Fig. A5) Furthermore, at distances $\alpha d = 12$, selection has little effect on neutral diversity, and VolcanoFinder uses this as a cutoff for the inclusion of data in the statistical test. In the case of strong selection, $2Ns = 1000$, this yields a cutoff at 120 kb from the beneficial mutation, while for weak selection, $2Ns = 100$, the cutoff is 12 kb from the beneficial mutation.

We first identify the independent peaks in our data. The first peak is defined as the test site with the highest LR value. Whether through a selective process or a neutral process, adjacent sites in the genome may give a strong but correlated signal, for example, a series of test sites close to the center of the sweep. Using the optimum α value, we define the breadth of this signal as the flanking region within distance $d_{max} = 12/\alpha$ left and right of the test site. We mask all test sites in this region, and define the second peak as the test site with the highest LR value in the remaining unmasked portion of the genome, repeating this process to obtain a set of candidate regions. Here, we limit the analysis to the top 50 candidate sites.

The way we define independent signals in the genome scan naturally extends to distinguishing the true signal from false positives. A peak is a true positive detection of the adaptive introgression allele if the beneficial allele is located within the breadth of the sweep region as determined by VolcanoFinder and the limit of $\alpha d = 12$ above. To visualize the overall power, we rank each independent adaptive introgression simulation as above. We then plot the proportion of simulation runs that have rank greater than or equal to $k = 0, 1, 2, ..., 50$.

The peak-finding approach

The sweep-width approach to outlier identification works well for methods like `VolcanoFinder` and `SweepFinder2`. However, it can only be applied to outlier-based genome scans for positive selection. In order to make comparisons to other methods when assessing the power and robustness of `VolcanoFinder`, we use a more general approach. Here, we identify the strength of the true-positive outlier signal as the highest likelihood ratio value observed for a small region of the genome that flanks the beneficial mutation. The outlier scores from the remainder of the genome are treated as false-positives. To distinguish independent signals among the genomic background scores, we identify the peaks in LR values occurring along the genome. Defining a minimum distance between adjacent peaks in the genome, we merge neighboring peaks to obtain a set of false-positive candidate regions. The subsequent evaluation of `VolcanoFinder`'s performance proceeds as for the sweep-width approach.

Text S3.2

Power analysis: 10 Mb chromosomes

In this section, we investigate the power of `VolcanoFinder` to detect the adaptive introgression allele in the context of a single chromosome. Ideally, we should simulate a large chromosome in order to obtain a representative distribution of scores in the neutral genomic background. We implemented simulations of the adaptive introgression sweep using `SLiM3` [1] with tree-sequence recording to model selection, then *recapitation* in `msprime` [2] to simulate neutral coalesence for the demographic history specified in our model. However, using realistic parameters for humans, even these advanced methods were computationally prohibitive, and we were limited to 10 Mb genomic regions, corresponding to 20 centiMorgans of human chromosome for our chosen parameters.

Here, we consider a population of $N = 10,000$ diploid individuals, mutation rate $\mu = 1.25 \times 10^{-8}$ per site per generation, and recombination rate $r = 5 \times 10^{-7}$ per site per generation. The strength of selection acting on the beneficial mutation is either $2Ns = 1000$ (strong) or $2Ns = 100$ (weak). We model adaptive introgression occuring from a highly-diverged donor population, $T_d = 4.0$ or $T_d = 2.5$ (units of $4N$ generations). The donor population size is set to $N = 1$ diploid individual to ensure the adaptive introgression event stems from a single haplotype. Conditioning on fixation of the adaptive allele, we sampled a single outgroup lineage with divergence $T_{sp} = 10.0$ ($4N$ generations) to polarize the data and $n = 40$ chromosomes from the recipient population at the time of fixation of the beneficial mutation.

Two scenarios are included in this analysis. The first scenario models adaptive introgression from a rare migration/hybridization event. In this case, migration from the donor to the recipient population occurs for a single generation rate with migration rate $m = 1/N$. This closely resembles the scenario in Fig. B3, Panel 3-1. However, there is a key difference. Here, the genomic background experiences a small amount of neutral introgression, while in the main text, the background is purely non-admixed.

We then extend this setting to a second scenario where high hybridization rates lead to a larger amount of introgression in neutral regions of the genome, setting $m = 30/N$ for $2Ns = 1000$ and setting $m = 300/N$ for $2Ns = 100$ to ensure in each case a 95% chance of fixation of the adaptive mutation (see Fig. B1 and Text S2.1). That way, the model more accurately reflects the type of hybridization events that result in fixation of the adaptive allele. It is this type of event we expect to observe most often in real data. Note, however, that we still condition on fixation of the adaptive allele in this scenario. These cases correspond fully to Fig. 5, Panel 2-1 ($T_d = 2.5$) and Panel 3-1 ($T_d = 4.0$).

The effect of migration and divergence

For introgression from a rare hybridization event, `VolcanoFinder` has very high power to detect the adaptive introgression sweep, both for weak and strong selection (Fig. C1, panel A). Panel B shows the results for the high-migration scenario. In this case, we observe high power with strong selection but relatively low power for weak selection. In panel B, we also observe that increasing the divergence of the donor population from $T_d = 2.5$ (blue lines) to $T_d = 4.0$ (green lines) yields only a marginal increase in power to detect the adaptive introgression allele.

To understand this more clearly, we compared the true-positive test scores from the sweep center to the distribution of peak scores taken from the peripheral 1000 kb regions of the chromosomes (Fig. C2). Note that Fig. C1 shows the power to detect the adaptive introgression allele in a 10 Mb chromosome, whereas here, the false-positive outlier peaks are obtained from a total of 480 Mb of simulated sequence data. When selection is strong, we observed high-valued outlier scores in the sweep center across all simulated scenarios. When selection is weak, however, the signal of the selective sweep

does not stand out from the peaks in the background distribution. When hybridization is rare (low migration), we observed stronger outlier signals in the genomic background for strong selection relative to weak selection. At the periphery of the chromosome, we do not expect selection to effect the test scores. Rather, this is an effect of sampling time, as fixation of the beneficial mutation occurs much earlier when selection is strong. Fig. C2 also shows that, although increased migration rates lead to overall higher test scores in the genomic background, the heights of the outlier peaks are reduced. In contrast, higher migration rates lead to much lower test scores in the central sweep region and a lower power to detect the adaptive introgression allele. Finally, we observed that higher divergence of the donor population leads to overall higher test scores both in the sweep center and in the strength of false positive peaks from the genomic background.

The effect of test site density

In Fig. C1 panel B, we compared the power to detect the sweep using a fine grid of test sites (250 bp between test sites, solid lines) and a coarse grid of test sites (1000 bp spacing, dashed lines). For strong selection, the coarse grid is sufficient to identify the true positive signal, and only with low divergence did we observe higher power with a more dense grid of test sites. In contrast, for weak selection, a higher density of test sites did result in a substantial increase in power. In order for `VolcanoFinder` to identify the sweep, a test site needs to be present in the central region that sweeps to fixation, and this region is much larger for strong selective sweeps. Furthermore, due to the stochastic nature of recombination, the sweep region is not necessarily centered on the position of the beneficial mutation. Indeed, we found that the test score at the selected site is in-general not the highest-scoring site within the 20 kb region at the sweep center (Fig. C3).

The effect of selection on nearby test scores

Because our simulated chromosomes represent 20 centiMorgans of sequence, we can safely assume that the scores in the peripheral regions of the simulated chromosome reflect the scores from a genomic background that experiences purely-neutral introgression. However, it is unclear how far the signal of adaptive introgression extends from the sweep center. We previously discussed that at distances $\alpha d = 12$, there is only a small effect of the sweep on the genealogy of sites linked to the beneficial mutation. This amounts to 120 kb for $2Ns = 1000$ and 12 kb for $2Ns = 100$, and at these distances, it is generally agreed that the effect of the sweep is small [3]. For a classic sweep from a *de novo* mutation, this makes sense. Consider the distance where only one lineage is caught in the sweep. In this case, the sweep does not affect the genealogical history of the sample. The lineages are exchangeable and follow a neutral coalescent process pastward. In the case of adaptive introgression, however, coalescence with the single lineage caught in the sweep occurs only in the common ancestral population. For this reason, it is possible that the sweep generates elevated signals even at relatively large distances from the beneficial mutation. Here, we examine the effect of the sweep on test scores in the flanking region of the genome.

To do this, we use the Total Operating Characteristic (TOC) [4] curve to compare the scores obtained from 100 kb windows of increasing distance from the beneficial mutation to the scores taken from the peripheral 500 kb of the chromosome, pooling the scores across independent replicate simulations. Fig. C4 shows the TOC curve for the low-migration high-divergence scenario of Fig. C1 panel A. Our concern is with the strength of outlier scores, and we therefore focus on high threshold values (lower panels of Fig. C4). When selection is strong, `VolcanoFinder` has high diagnostic power in the central 100 kb region of the sweep. Diagnostic power drops considerably beyond this

central region. However, we see that for high threshold values, scores are elevated relative to the background even at distances of 500 kb, much larger than we would expect based on the 120 kb sweep breadth discussed above. As expected, when selection is weak, the diagnostic power is lower, even in the center-most region of the sweep. However, we again observed elevated test scores much farther than the expected 12 kb region of the sweep.

Fig. C5 shows the TOC curves for the high-migration high-divergence scenario (solid green lines of Fig. C1 panel B). When selection is strong, `VolcanoFinder` has high diagnostic ability at high threshold values only in the center-most region of the sweep, and in this region, the diagnostic power is reduced relative to the low-migration scenario. When selection is weak, even the scores of the central sweep region are not diagnostic of the adaptive introgression event. This corroborates that the true positive test scores are difficult to distinguish from the distribution of outlier peaks obtained from a larger genomic background, as observed in Fig. C2.

Fig. C1

Power to detect the adaptive introgression allele Here we plot the detection probability for `VolcanoFinder` as a function of the number of false positive signals we observe. Panel A shows introgression from a rare hybridization event. Panel B shows introgression with higher migration rates. In panel B, the dashed lines are for 1000 bp spacing between test sites while the solid lines correspond to 250 bp spacing between sites. In both panels, divergence of the donor is $T_d = 4.0$ in units of $4N$ generations. Dark green shows results for strong selection $2Ns = 1000$ and light green shows results for weak selection $2Ns = 100$.

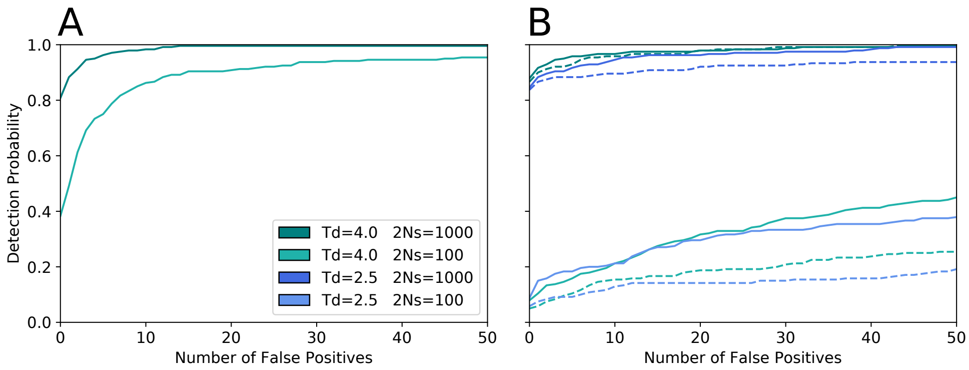

Fig. C2

Distribution of test scores This data corresponds to that of Fig. C1. Each data set contains 240 iterations. For each iteration, we obtain the highest likelihood ratio value within 40 kb of the beneficial mutation. We compare this to the distribution of test scores in the genomic background, here defined defined as the peripheral 1 Mb region taken from each chromosome (in total, 480 Mb). From the genomic background, we obtain a set of peak scores by merging neighboring test scores and setting a minimum distance of 20 kb between adjacent peaks. For each data set, the central region scores are shown on the left while the peak scores from the background are shown on the right. High divergence $T_d = 4.0$ is shown in green while low divergence $T_d = 2.5$ is shown in blue. The darker colors show strong selection; the lighter, weak selection.

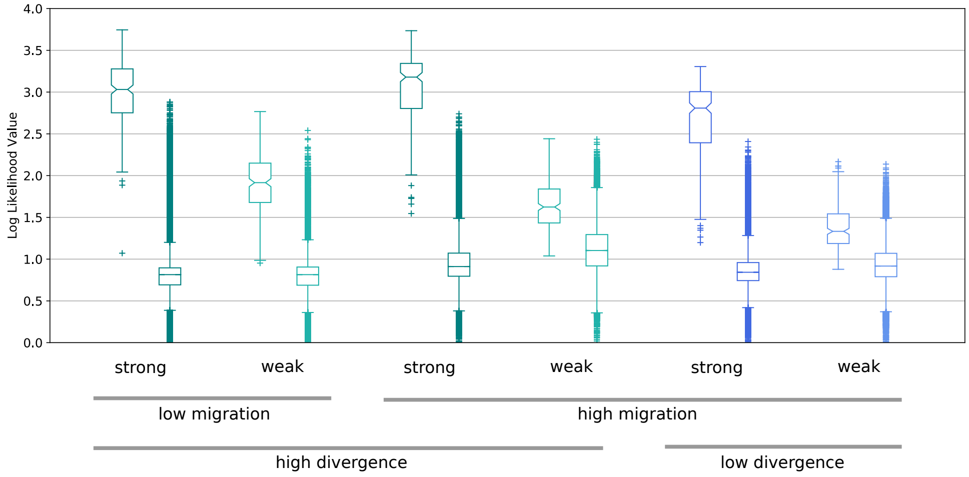

Fig. C3

Test scores in the sweep center This data corresponds to that of the high migration scenarios in Fig. C1. We plot the difference between the test score of the adaptive allele and the maximum score observed in the central 20 kb region of the sweep for each of the 240 iterations: a positive value indicates the score of the adaptive allele is the highest. The parameters are follows. Panel A: $2Ns = 1000$, $T_d = 4.0$, Panel B: $2Ns = 100$, $T_d = 4.0$, Panel C: $2Ns = 1000$, $T_d = 2.5$, and Panel D: $2Ns = 100$, $T_d = 2.5$.

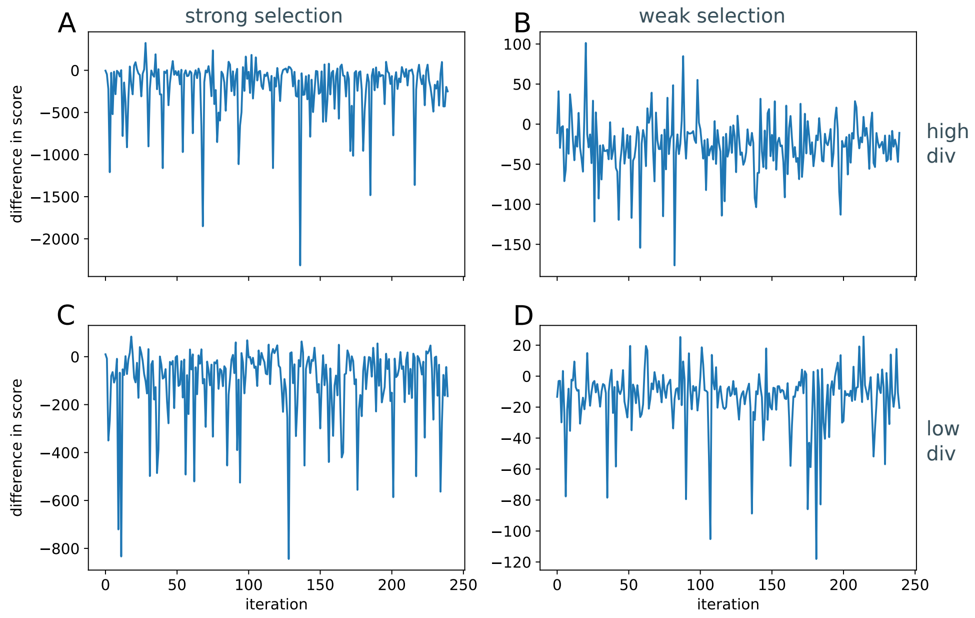

Fig. C4

Test scores in the sweep region (low migration) Here we show the Total Operating Characteristic (TOC) curves that compare scores from 100 kb regions of increasing distance from the beneficial mutation to the scores taken from the peripheral 500 kb of the chromosome, combining scores across the 240 replicate simulations. This data corresponds to Fig. C1, i.e. hybridization is rare $m = 1/(2N)$ and divergence of the donor is $T_d = 4.0$. The left column shows strong selection; the right, weak selection. The upper panels show the full range of threshold values, while the lower panels focus on high threshold values. The black dashed line corresponds to no difference between the two distributions ($x = y$), and the blue dashed lines show the maximum and minimum growth curves.

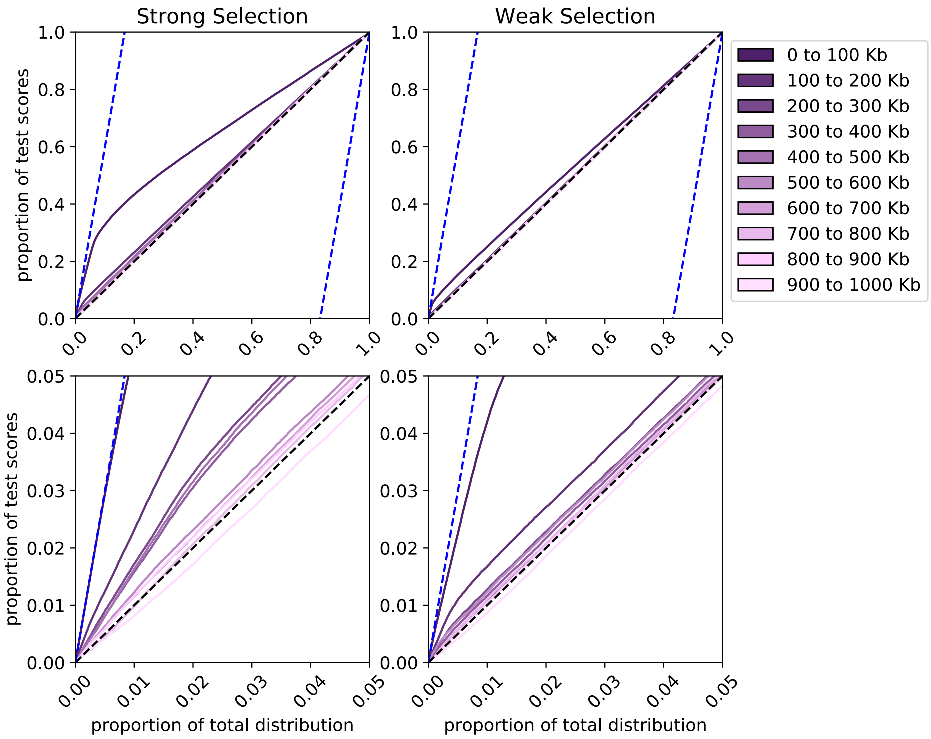

Fig. C5

Test scores in the sweep region (high migration) Here we show the Total Operating Characteristic (TOC) curves that compare scores from 100 kb regions of increasing distance from the beneficial mutation to the scores taken from the peripheral 500 kb of the chromosome, combining scores across the 240 replicate simulations. This data corresponds to Fig. C1, i.e. hybridization is rare $m = 1/(2N)$ and divergence of the donor is $T_d = 4.0$. The left column shows strong selection; the right, weak selection. The upper panels show the full range of threshold values, while the lower panels focus on high threshold values. The black dashed line corresponds to no difference between the two distributions $(x = y)$, and the blue dashed lines show the maximum and minimum growth curves.

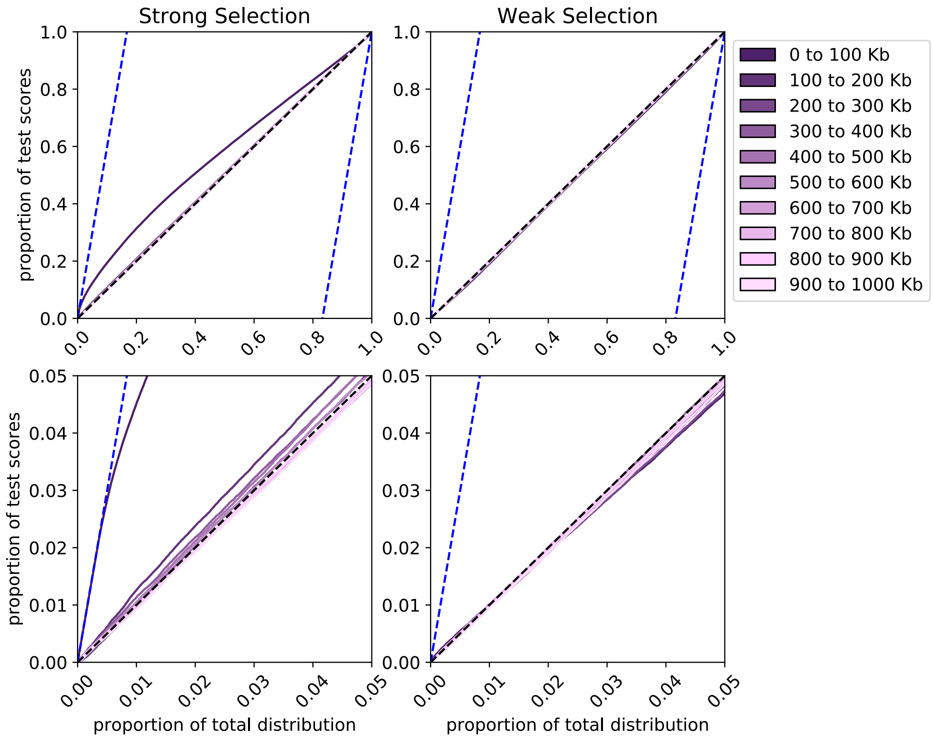

Text S3.3

Robustness to Classic Sweeps

Although the underlying model differs substantially from that of `SweepFinder2`, `VolcanoFinder` may have power to detect classic sweeps, particularly when low divergence values, e.g. $D = \theta$ are included in the grid search. Here we investigate the extent to which `VolcanoFinder` is able to detect a classic sweep from a *de novo* beneficial mutation, and we compare the performance of `VolcanoFinder` to that of `SweepFinder2`.

We simulate this scenario using `SLiM3` [1] and `msprime` [2] (as in section Text S3.2), placing the beneficial mutation in the center of a 10 Mb chromosome. However, in this case, the beneficial mutation arises in an individual chosen uniformly at random from a panmictic population. We then perform both `VolcanoFinder` and `SweepFinder2` scans on the data. Here, we employ the power analysis that uses the predicted sweep width to distinguish independent outliers and identify the true-positive signal.

For a classic sweep from *de novo* mutation, as expected, `SweepFinder2` has very high power to detect a strong sweep with $2Ns = 1000$ but has only moderate power for weak selection with $2Ns = 100$ (Fig. C6, Panel A). In Panel B, we observe moderate power for `VolcanoFinder` to identify a classic sweep with strong selection but little-to-no power when selection is weak. Interestingly, by comparing to Fig. C1, we observe that the power of `VolcanoFinder` to detect an adaptive introgression sweep is similar to the power of `SweepFinder2` to detect a classic sweep.

Fig. C6 Panel C compares the distribution of true-positive test scores to the distribution of test scores taken from the periphery of the simulated chromosomes. We observe that the range of high-valued outlier scores in the background distribution from `VolcanoFinder` is much lower than in the adaptive introgression scenario (Fig. C2), much more closely resembling the outlier scores obtained here by `SweepFinder2`. However, while the range of outlier scores is similar, the overall distribution of scores is higher for `VolcanoFinder` scans. In this figure, the background scores are taken from 480 Mb of genome. As we observed for `VolcanoFinder` in Fig. C2, `SweepFinder2` retains high power to detect the classic sweep signal in the context of a larger genome, but for weak selection, the true-positive test scores do not stand out from the background.

For adaptive introgression sweeps, we observed elevated test scores even for sites relatively distant from the sweep center. In contrast, we observe that the signal of a classic sweep is restricted to only a very small region in the sweep center (Fig. C7). For `VolcanoFinder`, a diagnostic signal is observed only when selection is strong, and as we would expect, `SweepFinder2` has strong diagnostic power when selection is strong and moderate diagnostic power at high thresholds when selection is weak. For both methods, we observe that elevated test scores are restricted to the 25 kb region flanking the beneficial mutation, a much more narrow region than we observed for `VolcanoFinder` applied to data simulated under an adaptive introgression scenario (Fig. C4 and Fig. C5).

Fig. C6

Classic Sweep: detection probability and distribution of test scores Panels A and B show the power to detect a classic sweep from a *de novo* beneficial mutation for `SweepFinder2` (blue) and `VolcanoFinder` (green), respectively. In each, the dark color corresponds to strong selection with $2Ns = 1000$ while the light color corresponds to weak selection with $2Ns = 100$. For each of these four cases, panel C compares the distribution of true-positive test scores (left) to the background signal obtained by pooling the data from the peripheral 1 Mb of the simulated chromosomes (total 480 Mb of background sequence). We simulate a 10 Mb chromosome with the adaptive allele placed in the center. Here, the adaptive allele arises as a new beneficial mutation in a panmictic population, and we sample the population at the time of fixation of the adaptive allele. Here, $N = 10,000$, $\mu = 1.25 \times 10^{-8}$, and $r = 5 \times 10^{-7}$.

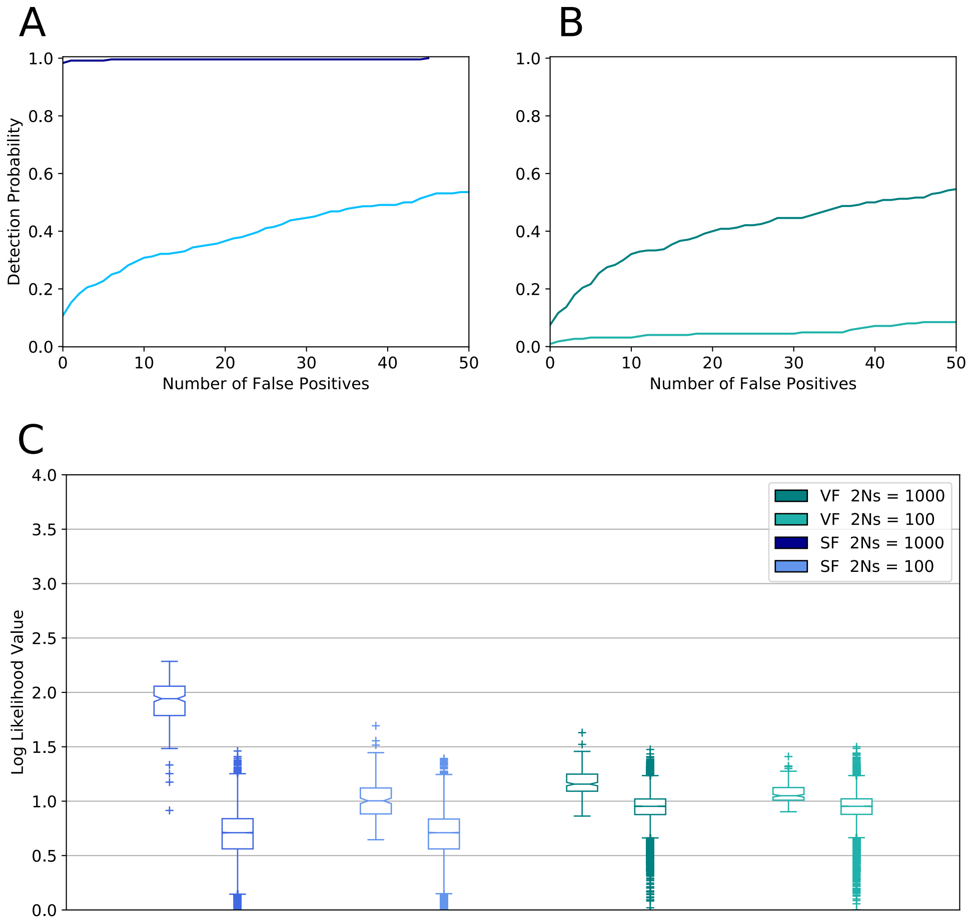

Fig. C7

Classic Sweep: Test scores in the sweep region Here we show the Total Operating Characteristic (TOC) curves that compare scores from 12 kb regions of increasing distance from the beneficial mutation to the scores taken from the peripheral 500 kb of the chromosome, combining scores across the 240 replicate simulations. This data corresponds to Fig. C6. The left column shows scores from the `VolcanoFinder` scans; the right column, from `SweepFinder2` scans. The top row shows the results for strong selection, the bottom row results for weak selection. The black dashed line corresponds to no difference between the two distributions ($x = y$), and the blue dashed lines show the maximum and minimum growth curves.

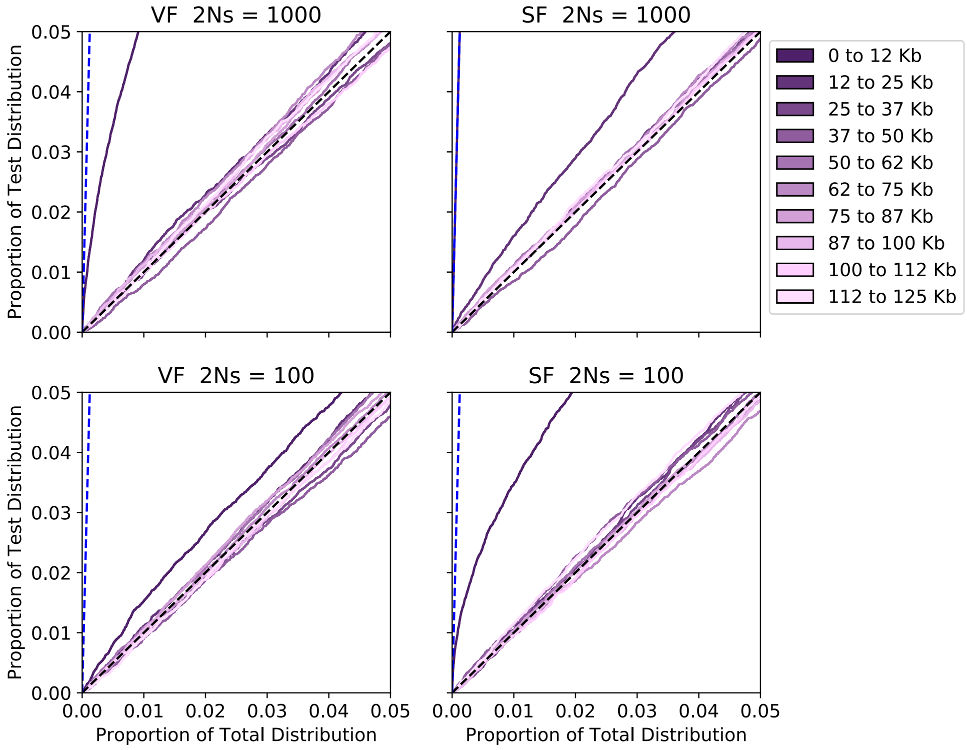

Text S3.4

Robustness to Background Selection

Background selection refers to the loss of genetic variation at neutral sites due to the purging of nearby deleterious mutations that arise throughout the genome [5]. In the coalescent framework, the effect of background selection translates to a local reduction in the population size and thus shorter branch lengths in the genealogical history of a sample relative to that of a purely neutral evolutionary model [6, 7]. In this way, over relatively short distances in the genome, background selection mimics the effect of a selective sweep [8]. While statistical tests of positive selection based on pairwise genetic diversity are robust to the effects of background selection [9], methods that are based on the site frequency spectrum are more susceptible, as background selection induces a skew toward low-frequency alleles.

Here, we are primarily interested in the robustness of `VolcanoFinder` to false signatures of adaptive introgression generated by background selection. We therefore investigate how the action of ubiquitous background selection affects the distribution of likelihood ratio values in a genome that does not experience a selective sweep. We do this by simulating both a set of neutral simulations and a comparable set of simulations in which we model background selection. We then compare the distributions of likelihood ratio values found by `VolcanoFinder` under these two scenarios.

To evaluate the effect of background selection on inferring adaptive introgression, we employed the forward-time simulator `SLiM3` [1]. We simulated two sets of sequences: one set with background selection and one without. For simulations with background selection, we use realistic gene structures based on protein-coding gene annotations from the RefSeq database. For each set of background selection or neutral scenarios, 500 replicates were generated for downstream analyses. We simulate a simplified human-chimpanzee evolutionary history, in which all species evolve with a constant population size of 10^4 diploid individuals ([10]), and have uniform per-generation mutation and recombination rates of 2.5×10^{-8} and 10^{-8} per nucleotide, respectively (parameters approximated from [11] and [12]). We adopted a generation time of 20 years, and the species divergence time of five million years (i.e., 2.5×10^5 generations) (parameters approximated from [13]). As is common practice with forward-time simulators, a scaling parameter $\lambda = 100$ was used to accelerate the simulations. At the end of each simulation replicate, 50 diploids in the target (human) species and one diploid in the outgroup (chimpanzee) species are sampled and parsed, with the first haploid of the sampled outgroup individual considered as the ancestral sequence.

To model background selection, we let a certain proportion of all the mutations in a functional element to be recessively deleterious, with a dominance coefficient of 0.1 and a per-generation selective coefficient s randomly drawn from a gamma distribution with a mean of -0.005 and a shape parameter of 0.2 [14]. The proportions of such mutations differ by the element type. We set exons to have 75% of all mutations being deleterious, upstream and downstream UTRs with 50% mutations deleterious, and introns with 10% mutations deleterious (following [15]). For 100 kb simulated sequences with background selection, we modeled a "protein-coding gene" region with 5'- and 3'-UTRs, 11 exons, and 10 introns at the center of each sequence. The lengths of the modeled 5'-UTRs, 3'-UTRs, exons, and introns are 200, 800, 100, and 1000 bp, respectively.

Fig. C8 shows the effect of background selection on the distribution of test scores in the genomic background. Panels A and B show the TOC curve comparing the background selection scores to those obtained under neutrality. Across the full range of lower threshold values (Panel A), we observe no difference between the two distributions. At very high threshold values (Panel B) we observe a diagnostic signal of background selection, however, in Panel C, we observe that this is only due to a slight increase in

likelihood ratio values for the most-extreme outlier peaks relative to test scores obtained for the neutral simulations. The strength of these outlier signals pales in comparison to the true-positive scores obtained under a selective sweep model (see Fig. C2), and we therefore conclude that `VolcanoFinder` is robust to the effects of background selection.

Fig. C8

The effect of background selection on test scores Panels A and B show the Total Operating Characteristic (TOC) curve, which compares the scores obtained under ubiquitous background to those obtained under purely neutral evolution. In Panel A, we show the full range of threshold values, while in Panel B we focus on high threshold values. Panel C shows the distribution of test scores under neutrality (grey) and background selection (black).

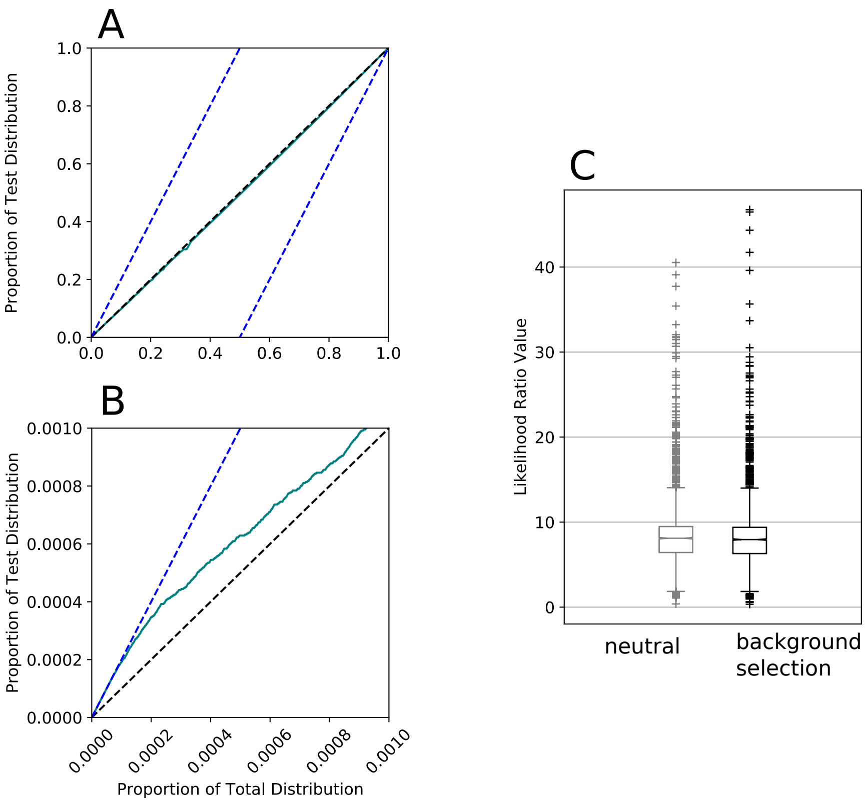

Text S3.5

Chimeric chromosomes

In our power analysis we present two approaches to identify independent outlier signals in genomic scan data (see section Text S3.1). One is based on the sweep-width of the optimum adaptive introgression model as obtained by `VolcanoFinder`; the other peak-finding approach uses as the true-positive signal the highest likelihood-ratio value obtained in the small genomic region flanking the beneficial mutation and compares this to the false-positive peak signals that lay outside this central sweep region.

In the context of the simulated 10 Mb genomic regions, we have primarily used the sweep-width approach to identify peak signals in the data (see Fig. C1). For this same data set, Fig. C9 (solid lines) shows the power to detect the adaptive introgression allele when outlier signals were identified using the peak-finding approach. We observed that the two approaches to signal identification yield consistent results with respect to the probability of detecting the adaptive introgression sweep.

We have also used two different simulation approaches to generate data for the power analyses. Ideally, we would like to simulate a very large chromosome in which the adaptive introgression event occurs. However, even using the current methods combining `msprime` [2] and `SLiM3` [1], we were limited to 10 Mb genomic regions. Furthermore, due to the computational cost of these simulations, we were limited to testing only a handful of adaptive introgression scenarios. For that reason, we used an alternative simulation procedure for the more-extensive power analysis of the main text.

In the main text, we considered the power to detect the adaptive allele in the context of a large genomic background. Here, independent 200 kb regions with a selective sweep were simulated using a purely coalescent-based approach. In addition, many replicate simulations under neutrality were pooled to create a large genomic background. This approach is much less computationally taxing, and it allowed us to perform a much more expansive power analysis. However, a major concern is whether the genetic variation pooled across replicate neutral simulations accurately represents that which we would observe in a contiguous large chromosome.

To test this, we compare the power of detecting the adaptive introgression sweep in the contiguous 10 Mb chromosomes to that in equally-sized *chimeric* chromosomes constructed from the same data, here using the peak-finding approach to identify false-positive signals. We built these so-called chimeric chromosomes as follows. From each iteration, we extract the 40 kb of sequence centered on the beneficial mutation, and from this, we take the highest likelihood ratio value as the true-positive test score. We then reconstitute a genomic background by randomly sampling 200 kb segments from the background scores across all iterations (excluding the central 40 kb windows containing the beneficial mutation). In Fig. C9, we observed little-to-no difference in power to detect the adaptive introgression sweep when using chimeric chromosomes (dashed lines) rather than the original contiguous 10 Mb chromosomal regions (solid lines). This supports the use of many independent replicates as a proxy for a large genomic background in the power analysis of the main text.

Fig. C9

Comparing peak-identification and simulation methods Here we plot the power to detect the adaptive introgression sweep using the peak-finding approach to identify independent outlier signals in the genomic background. The data in this figure matches that of Fig. C1, in which outlier signals were identified using the sweep-width approach. The left panel shows low migration while the right panel shows high migration. In both panels, $T_d = 4.0$, dark green corresponds to strong selection with $2Ns = 1000$, and light green to weak selection with $2Ns = 100$. Here, the solid lines show the probability of detecting the adaptive allele in the context of a contiguous 10 Mb genomic region. The dashed lines show the power to detect the sweep in 10 Mb *chimeric chromosomes* (see section Text S3.5 for description). Here, the true-positive test score is defined as the highest likelihood-ratio value obtained within 20 kb of the beneficial mutation. From the remaining genetic variation in the simulated region, we identified false-positive signals by identifying peak likelihood-ratio values and merging adjacent peaks separated by less than 20 kb.

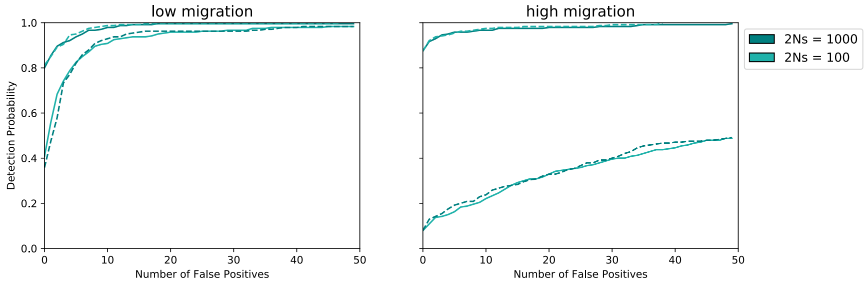

References

1. Haller BC, Messer PW. SLiM 3: Forward Genetic Simulations Beyond the Wright–Fisher Model. Molecular Biology and Evolution. 2019;36(3):632–637. doi:10.1093/molbev/msy228.

2. Kelleher J, Etheridge AM, McVean G. Efficient coalescent simulation and genealogical analysis for large sample sizes. PLoS computational biology. 2016;12(5):e1004842.

3. McVean G. The Structure of Linkage Disequilibrium Around a Selective Sweep. Genetics. 2007;175(3):1395–1406. doi:10.1534/genetics.106.062828.

4. Jr RGP, Si K. The total operating characteristic to measure diagnostic ability for multiple thresholds. International Journal of Geographical Information Science. 2014;28(3):570–583. doi:10.1080/13658816.2013.862623.

5. Charlesworth B, Morgan MT, Charlesworth D. The effect of deleterious mutations on neutral molecular variation. Genetics. 1993;134(4):1289–1303.

6. Charlesworth D, Charlesworth B, Morgan MT. The pattern of neutral molecular variation under the background selection model. Genetics. 1995;141(4):1619–1632.

7. Nicolaisen LE, Desai MM. Distortions in genealogies due to purifying selection and recombination. Genetics. 2013;195(1):221–230.

8. Seger J, Smith WA, Perry JJ, Hunn J, Kaliszewska ZA, La Sala L, et al. Gene genealogies strongly distorted by weakly interfering mutations in constant environments. Genetics. 2010;184(2):529–545.

9. Schrider DR. Background selection does not mimic the patterns of genetic diversity produced by selective sweeps. bioRxiv. 2019;doi:10.1101/2019.12.13.876136.

10. Takahata N, Satta Y, Klein J. Divergence time and population size in the lineage leading to modern humans. Theoretical population biology. 1995;48(2):198–221.

11. Nachman MW, Crowell SL. Estimate of the mutation rate per nucleotide in humans. Genetics. 2000;156(1):297–304.

12. Payseur BA, Nachman MW. Microsatellite variation and recombination rate in the human genome. Genetics. 2000;156(3):1285–1298.

13. Kumar S, Filipski A, Swarna V, Walker A, Hedges SB. Placing confidence limits on the molecular age of the human–chimpanzee divergence. Proceedings of the National Academy of Sciences. 2005;102(52):18842–18847. doi:10.1073/pnas.0509585102.

14. Kim BY, Huber CD, Lohmueller KE. Inference of the distribution of selection coefficients for new nonsynonymous mutations using large samples. Genetics. 2017;206(1):345–361.

15. Cheng X, Xu C, DeGiorgio M. Fast and robust detection of ancestral selective sweeps. Molecular ecology. 2017;26(24):6871–6891.
