# Supplementary material for: VolcanoFinder: Genomic scans for adaptive introgression
Source: PLoS Genet. 2020 Jun 18;16(6):e1008867. doi: 10.1371/journal.pgen.1008867 (PMC7326285; doi:10.1371/journal.pgen.1008867)
Supplement: S4 Appendix — Supplementary material for the human data analysis. This includes (1) The Manhattan plots for YRI and CEU (2) The table of candidate loci for YRI and CEU (3) The additional genomic regions that are highlighted in the main text. (PDF) [file pgen.1008867.s004.pdf]

VolcanoFinder

genomic scans for adaptive introgression

S4 Power Analysis - 10 Mb Chromosome

Derek Setter[123☯¤], Sylvain Mousset[1☯], Xiaoheng Cheng[4], Rasmus Nielsen[5], Michael DeGiorgio[6‡], Joachim Hermisson[127‡],

1 Department of Mathematics, University of Vienna, Vienna, Austria
2 Vienna Graduate School of Population Genetics, Vienna, Austria
3 School of Biological Sciences, University of Edinburgh, Edinburgh, United Kingdom
4 Huck Institutes of the Life Sciences, Pennsylvania State University, University Park, PA, USA
5 Departments of Integrative Biology and Statistics, University of California, Berkeley, CA, USA
6 Department of Computer and Electrical Engineering and Computer Science, Florida Atlantic University, Boca Raton, FL, USA
7 Max F. Perutz Laboratories, University of Vienna, Vienna, Austria

☯These authors contributed equally to this work.
‡These authors also contributed equally to this work.
¤Current Address: School of Biological Sciences, University of Edinburgh, Edinburgh, United Kingdom
*Correspondence: joachim.hermisson@univie.ac.at (JH), mdegiorg@fau.edu (MD)

S4 Human Data

Table D1

Candidate peaks ranked by the maximum log likelihood ratios in the `VolcanoFinder` scan of the European (CEU) sample.

Chr.	Peak Position	L.R.	$-\log_{10}\hat{\alpha}$	$\hat{D}$	Nearest Gene(s)	RefSeq ID
22	36 556 023	102.4	3.42	0.0038	*APOL3*, *APOL4*	NM_145640.2, NM_030643.4
8	42 558 168	80.4	3.67	0.0023	*CHRNB3*, *CHRNA6*	NM_001347717.1, NM_004198.3
2	223 953 190	55.5	3.21	0.0061	*KCNE4*	NM_080671.3
14	81 512 174	51.5	3.73	0.0023	*TSHR*	NM_001018036.2
21	19 243 059	50.5	3.68	0.0015	*CHODL*, *CHODL-AS1*	NM_001204177.1, NR_024354.1
2	24 626 190	36.9	3.88	0.0015	*ITSN2*	NM_001348181.1
2	223 924 190	36.7	3.58	0.0030	*KCNE4*	NM_080671.3
3	182 989 072	35.4	3.48	0.0023	*MCF2L2*, *B3GNT5*	NM_015078.3, NM_032047.4
2	122 044 190	35.2	3.50	0.0015	*TFCP2L1*	NM_014553.2
2	172 059 190	33.7	3.49	0.0023	*TLK1*	NM_012290.4
7	73 935 618	32.8	3.31	0.0030	*GTF2IRD1*	NM_005685.3
2	12 035 190	29.6	3.35	0.0023	–	–
16	83 231 010	29.5	3.19	0.0015	*CDH13*	NM_001220491.1
3	127 624 072	29.2	3.49	0.0023	*KBTBD12*	NM_207335.2
11	44 436 084	29.0	3.30	0.0015	–	–
12	71 969 102	28.5	3.12	0.0038	*LGR5*, *ZFC3H1*	NM_001277227.1, NM_144982.4
19	17 289 015	27.9	3.20	0.0023	*MYO9B*, *USE1*, *OCEL1*	NM_004145.3, NM_018467.3, NM_024578.2
20	7 596 076	27.8	3.32	0.0023	–	–
10	28 705 072	27.4	3.47	0.0015	–	–
1	232 398 053	26.5	3.50	0.0015	–	–
3	129 080 072	25.6	3.22	0.0023	*RPL32P3*, *H1FX*, *H1FX-AS1*, *SNORA7B*, *EF-CAB12*	NR_003111.2, NM_006026.3, NR_026991.1, NR_002992.2, NM_207307.2
5	154 678 042	24.6	3.50	0.0015	–	–
13	105 399 042	24.4	3.28	0.0030	–	–
9	115 694 060	24.4	3.29	0.0015	*SLC46A2*	NM_033051.3
6	29 035 112	23.9	3.12	0.0023	*LOC100129636*, *OR2W1*, *OR2B3*, *OR2J3*	NR_125387.1, NM_030903.3, NM_001005226.2, NM_001005216.3
9	79 675 060	23.9	3.27	0.0015	*FOXB2*	NM_001013735.1
6	34 052 112	23.2	3.29	0.0023	*GRM4*	NM_000841.3

Table D2

Candidate peaks ranked by the maximum log likelihood ratios in the `VolcanoFinder` scan of the Yoruban (YRI) sample.

Chr.	Peak Position	LR	$-\log_{10}\hat{\alpha}$	$\hat{D}$	Nearest Gene(s)	Respective RefSeq ID
19	41 473 015	45.2	3.49	0.0020	*CYP2B7P*, *CYP2B6*	NR_001278.1, NM_000767.4
1	152 102 007	37.3	3.48	0.0030	*LOC100131107*, *TCHHL1*, *TCHH*, *RPTN*	NM_001310142.1, NM_001008536.1, NM_007113.3, NM_001122965.1
12	59 033 128	32.1	3.56	0.0020	*LOC101927653*, *LOC100506869*	NR_120452.1, NR_126341.1
3	33 007 016	32.0	3.48	0.0030	*CCR4*, *GLB1*	NM_005508.4, NM_001079811.2
2	170 442 117	28.7	3.25	0.0020	*FASTKD1*, *PPIG*	NM_001322046.1, NM_004792.2
2	235 174 117	25.1	3.09	0.0020	–	–
4	101 771 036	22.9	3.53	0.0020	–	–
4	78 105 036	22.2	3.31	0.0030	*CCNG2*	NM_004354.2

Fig. D1

Whole-genome Manhattan plot of the maximum likelihood ratio test statistic for the European (CEU) population computed from Model 1 of `VolcanoFinder` on data on within-CEU polymorphism and substitutions with respect to chimpanzee, and annotated with the top 18 gene candidates.

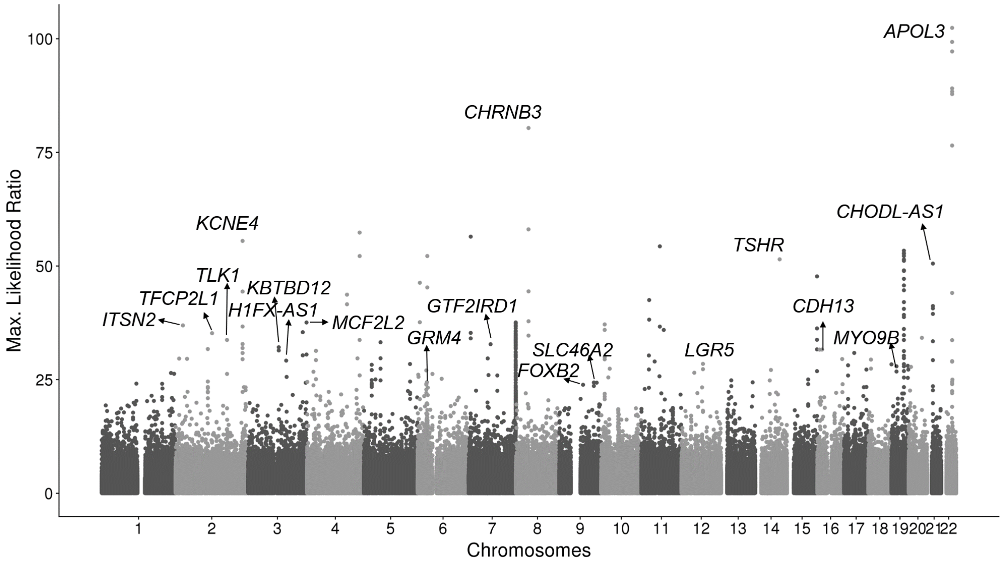

Fig. D2

Whole-genome Manhattan plot of the maximum likelihood ratio test statistic for the Yoruban (YRI) population computed from Model 1 of `VolcanoFinder` on data on within-YRI polymorphism and substitutions with respect to chimpanzee, and annotated with the top 7 gene candidates.

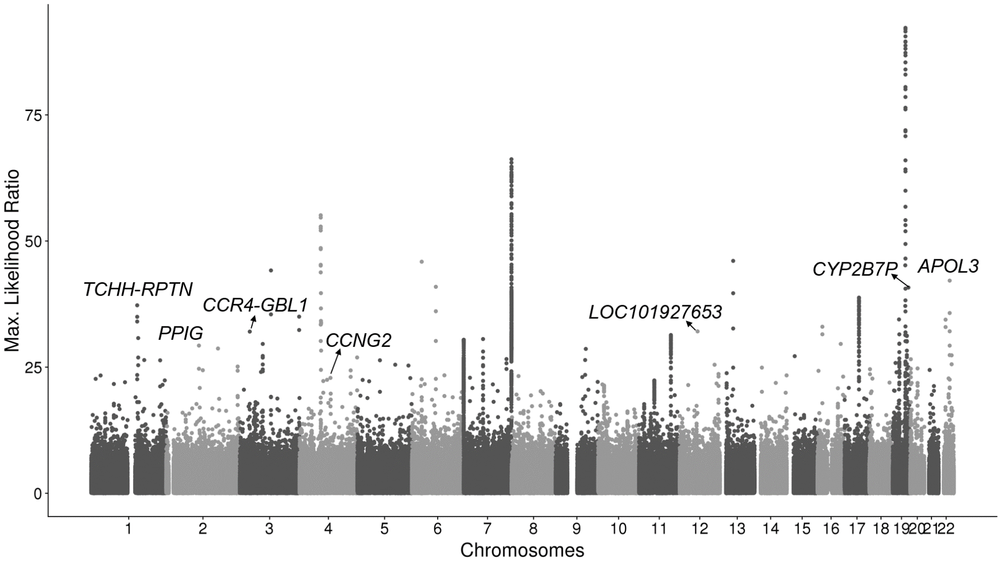

Fig. D3

Introgression sweep signals, parameter estimates, and sequencing properties across the 100 kb region on chromosome 22 covering *APOL* gene cluster in YRI.

A. Likelihood ratio test statistic computed from Model 1 of `VolcanoFinder` on data on within-YRI polymorphism and substitutions with respect to chimpanzee. Horizontal dark gray, medium gray, and light gray bars correspond to regions that were filtered based on Hardy-Weinberg equilibrium (HWE) test. Gene tracts and labels for key genes are depicted below the plot, with the wider bars representing exons. **B.** Values for α and divergence D corresponding to the maximum likelihood estimate of the data. Black line corresponds to $-\ln(\alpha)$ and vertical gray bars correspond to estimated D. **C.** Likelihood ratio test statistic computed from T_2 of `BALLET` on data on within-YRI polymorphism and substitutions with respect to chimpanzee using windows of 100 (black) or 22 (gray) informative sites on either side of the test site. **D.** Mean pairwise sequence difference ($\hat{\theta}_\pi$) computed in five kb windows centered on each polymorphic site. **E.** Mappability uniqueness scores for 35 nucleotide sequences across the region. **F.** Mean sequencing depth across the 108 YRI individuals as a function of genomic position, with the gray ribbon indicating standard deviation. The background heatmap displays the number of individuals devoid of sequencing reads as a function of genomic position, with darker shades of red indicating a greater number of individuals with no sequencing reads.

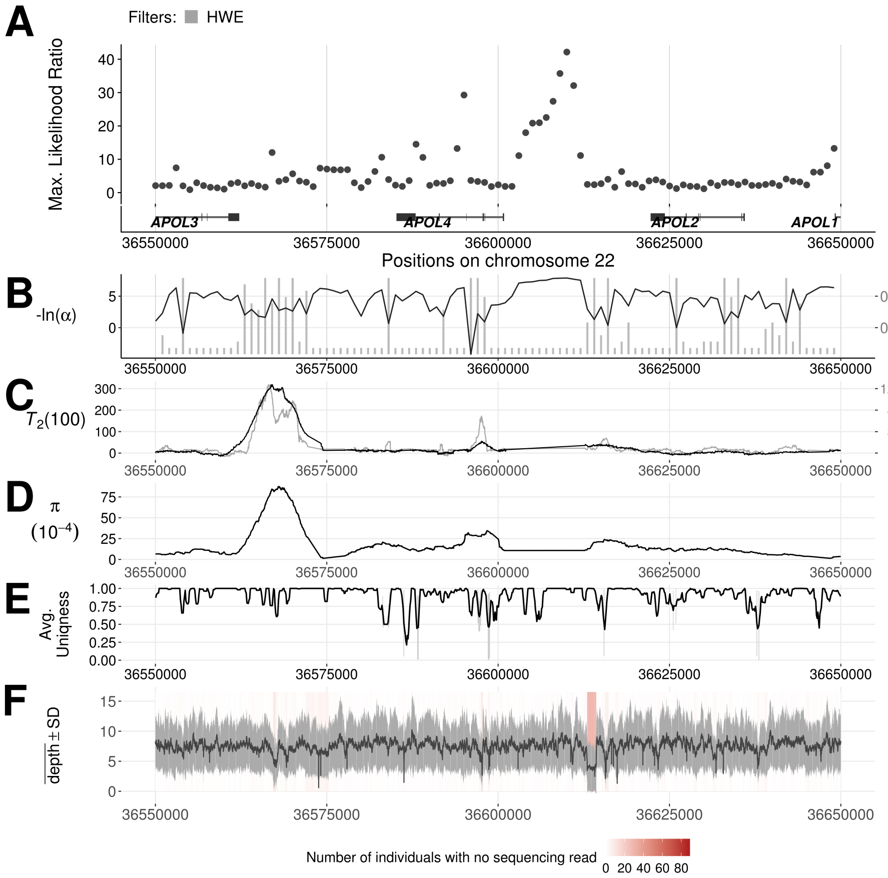

Fig. D4

Introgression sweep signals, parameter estimates, and sequencing properties across the 100 kb region on chromosome 22 covering *APOL4* gene in CEU, matching the same region in YRI.

A. Likelihood ratio test statistic computed from Model 1 of `VolcanoFinder` on data on within-CEU polymorphism and substitutions with respect to chimpanzee. Horizontal dark gray, medium gray, and light gray bars correspond to regions that were filtered based on Hardy-Weinberg equilibrium (HWE) test. Gene tracts and labels for key genes are depicted below the plot, with the wider bars representing exons. **B.** Values for α and divergence D corresponding to the maximum likelihood estimate of the data. Black line corresponds to $-\ln(\alpha)$ and vertical gray bars correspond to estimated D. **C.** Likelihood ratio test statistic computed from T_2 of `BALLET` on data on within-CEU polymorphism and substitutions with respect to chimpanzee using windows of 100 (black) or 22 (gray) informative sites on either side of the test site. **D.** Mean pairwise sequence difference ($\hat{\theta}_\pi$) computed in five kb windows centered on each polymorphic site. **E.** Mappability uniqueness scores for 35 nucleotide sequences across the region. **F.** Mean sequencing depth across the 108 YRI individuals as a function of genomic position, with the gray ribbon indicating standard deviation. The background heatmap displays the number of individuals devoid of sequencing reads as a function of genomic position, with darker shades of red indicating a greater number of individuals with no sequencing reads.

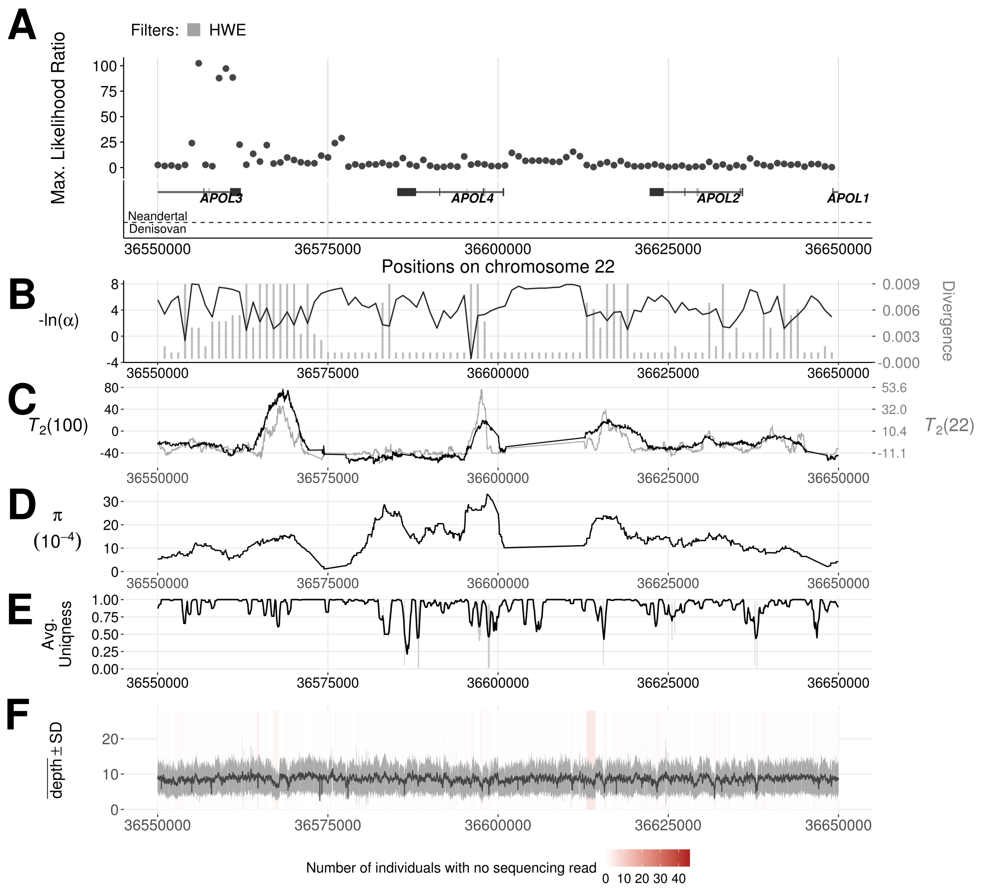

Fig. D5

Introgression sweep signals, parameter estimates, and sequencing properties across the one Mb region on chromosome 7 covering the *PTPRN2* gene region in YRI.

A. Likelihood ratio test statistic computed from Model 1 of `VolcanoFinder` on data on within-YRI polymorphism and substitutions with respect to chimpanzee. Horizontal dark gray and light gray bars correspond to regions that were filtered based on either mean CRG score or mean CRG score and proximity to a telomere, respectively. Gene tracts and labels for key genes are depicted below the plot, with the wider bars representing exons. **B.** Values for α and divergence D corresponding to the maximum likelihood estimate of the data. Black line corresponds to $-\ln(\alpha)$ and vertical gray bars correspond to estimated D. **C.** Likelihood ratio test statistic computed from T_2 of `BALLET` on data on within-YRI polymorphism and substitutions with respect to chimpanzee using windows of 100 (black) or 22 (gray) informative sites on either side of the test site. **D.** Mean pairwise sequence difference ($\hat{\theta}_\pi$) computed in five kb windows centered on each polymorphic site. **E.** Mappability uniqueness scores for 35 nucleotide sequences across the region. **F.** Mean sequencing depth across the 108 YRI individuals as a function of genomic position, with the gray ribbon indicating standard deviation. The background heatmap displays the number of individuals devoid of sequencing reads as a function of genomic position, with darker shades of red indicating a greater number of individuals with no sequencing reads.

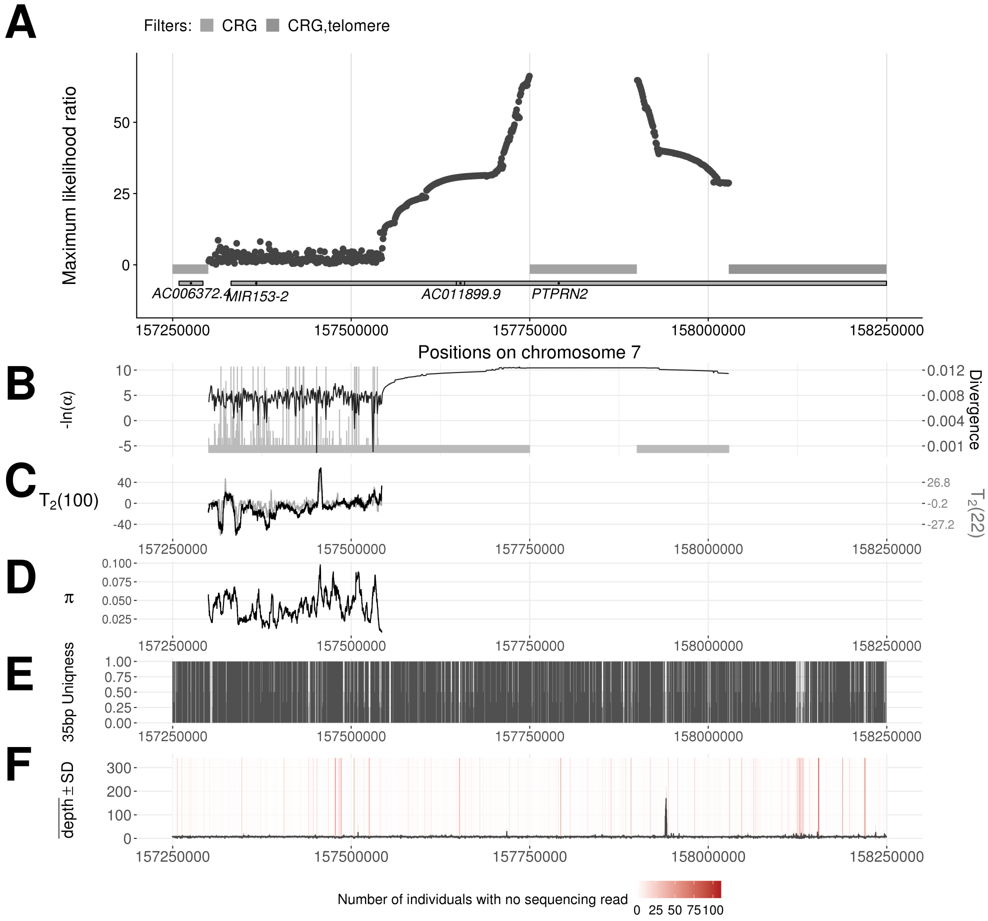

Fig. D6

Introgression sweep signals, parameter estimates, and sequencing properties across the one Mb region on chromosome 19 covering region surrounding *PCAT19* and *CEACAM4* genes in YRI.

A. Likelihood ratio test statistic computed from Model 1 of `VolcanoFinder` on data on within-YRI polymorphism and substitutions with respect to chimpanzee. Horizontal dark gray and light gray bars correspond to regions that were filtered based on Hardy-Weinberg equilibrium (HWE) test. Gene tracts and labels for key genes are depicted below the plot, with the wider bars representing exons. **B.** Values for α and divergence D corresponding to the maximum likelihood estimate of the data. Black line corresponds to $-\ln(\alpha)$ and vertical gray bars correspond to estimated D. **C.** Likelihood ratio test statistic computed from T_2 of `BALLET` on data on within-YRI polymorphism and substitutions with respect to chimpanzee using windows of 100 (black) or 22 (gray) informative sites on either side of the test site. **D.** Mean pairwise sequence difference ($\hat{\theta}_\pi$) computed in five kb windows centered on each polymorphic site. **E.** Mappability uniqueness scores for 35 nucleotide sequences across the region. **F.** Mean sequencing depth across the 108 YRI individuals as a function of genomic position, with the gray ribbon indicating standard deviation. The background heatmap displays the number of individuals devoid of sequencing reads as a function of genomic position, with darker shades of red indicating a greater number of individuals with no sequencing reads.

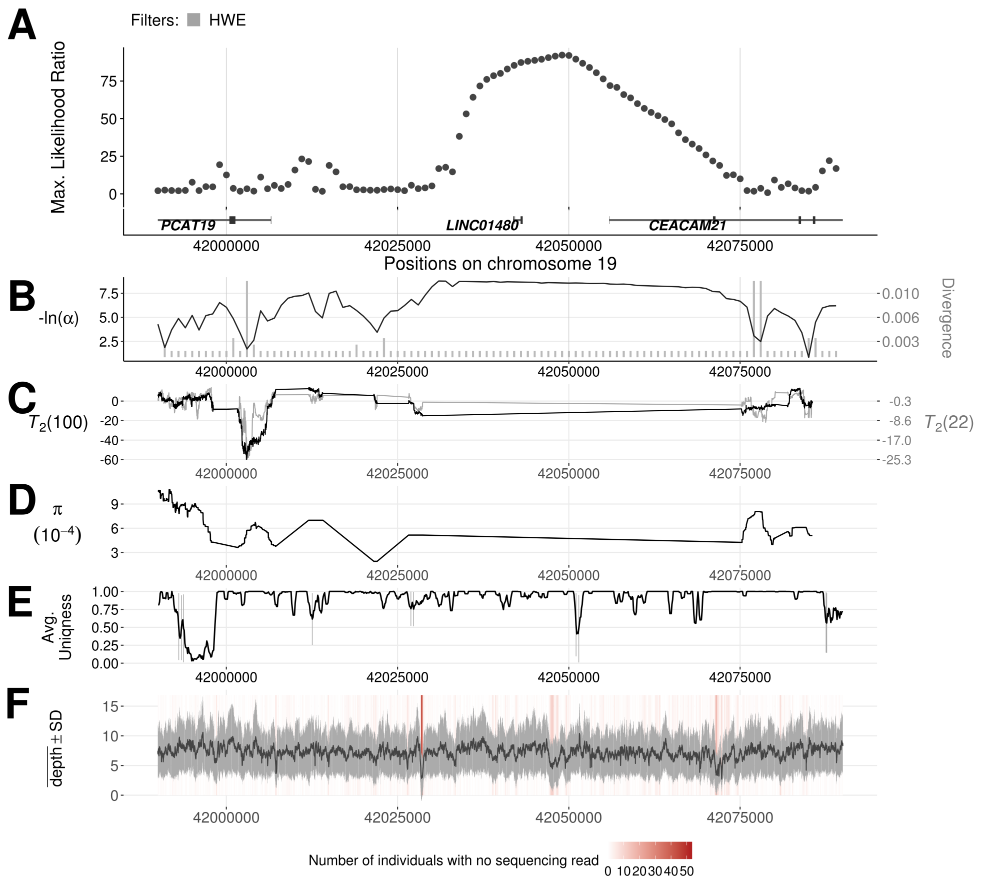

Fig. D7

Introgression sweep signals, parameter estimates, and sequencing properties across the one Mb region on chromosome 17 covering *IGFBP1* and *B4GALNT2* in YRI.

A. Likelihood ratio test statistic computed from Model 1 of `VolcanoFinder` on data on within-YRI polymorphism and substitutions with respect to chimpanzee. Horizontal dark gray and light gray bars correspond to regions that were filtered based on Hardy-Weinberg equilibrium (HWE) test. Gene tracts and labels for key genes are depicted below the plot, with wider bars representing exons. **B.** Values for α and divergence D corresponding to the maximum likelihood estimate of the data. Black line corresponds to $-\ln(\alpha)$ and vertical gray bars correspond to estimated D. **C.** Likelihood ratio test statistic computed from T_2 of `BALLET` on data on within-YRI polymorphism and substitutions with respect to chimpanzee using windows of 100 (black) or 22 (gray) informative sites on either side of the test site. **D.** Mean pairwise sequence difference ($\hat{\theta}_\pi$) computed in five kb windows centered on each polymorphic site. **E.** Mappability uniqueness scores for 35 nucleotide sequences across the region. **F.** Mean sequencing depth across the 108 YRI individuals as a function of genomic position, with the gray ribbon indicating standard deviation. The background heatmap displays the number of individuals devoid of sequencing reads as a function of genomic position, with darker shades of red indicating a greater number of individuals with no sequencing reads.

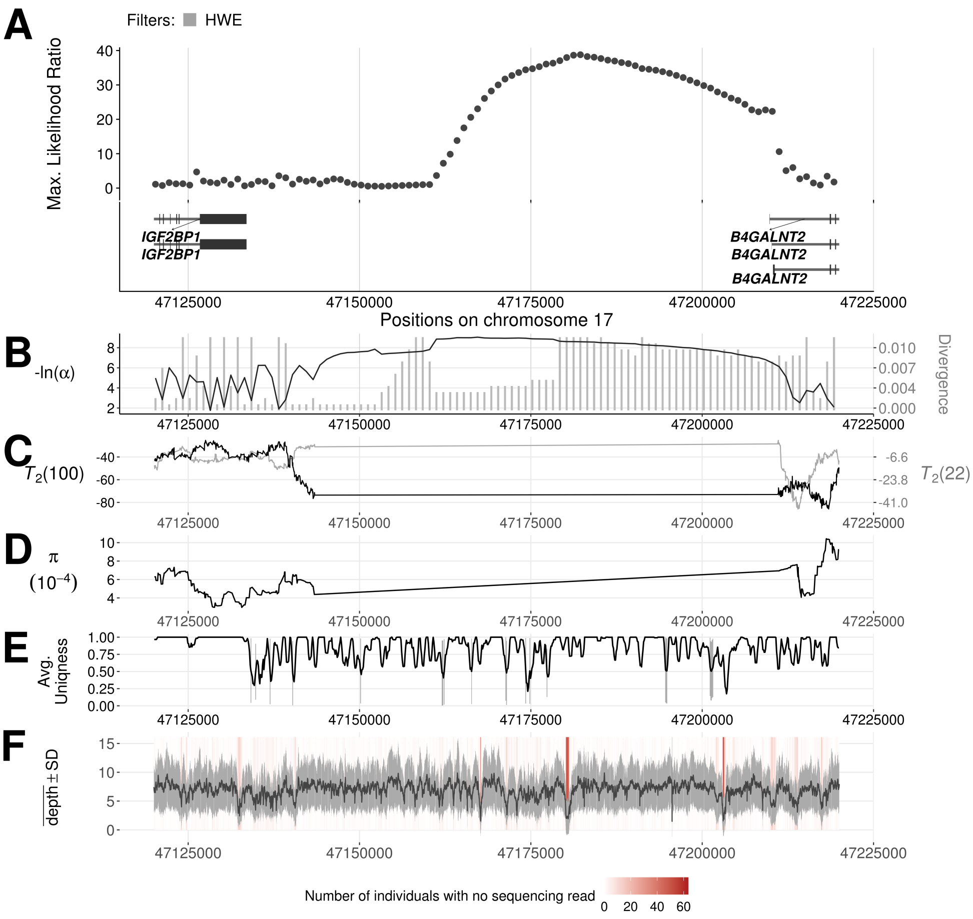

Fig. D8

Introgression sweep signals, parameter estimates, and sequencing properties across the 100 kb region on chromosome 19 covering the gene *MUC4* in CEU.

A. Likelihood ratio statistic computed from Model 1 of `VolcanoFinder` on the data of within-CEU polymorphism and substitutions with respect to the chimpanzee. Gray bars immediately below indicate the type of filters, and the longest gene transcripts are depicted with thick bars standing for exons. **B.** Values for α and divergence D corresponding to the maximum likelihood estimate of the data. Black line corresponds to $-\ln(\alpha)$ and vertical gray bars correspond to estimated D. **C.** Likelihood ratio test statistic computed from T_2 of `BALLET` on data on within-CEU polymorphism and substitutions with respect to chimpanzee using windows of 100 (black) or 22 (gray) informative sites on either side of the test site. **D.** Mean pairwise sequence difference ($\hat{\theta}_\pi$) computed in five kb windows centered on each polymorphic site. **E.** Mappability uniqueness scores for 35 nucleotide sequences across the region. **F.** Mean sequencing depth across the 99 CEU individuals as a function of genomic position, with the gray ribbon indicating standard deviation. The background heatmap displays the number of individuals devoid of sequencing reads as a function of genomic position, with darker shades of red indicating a greater number of individuals with no sequencing reads.

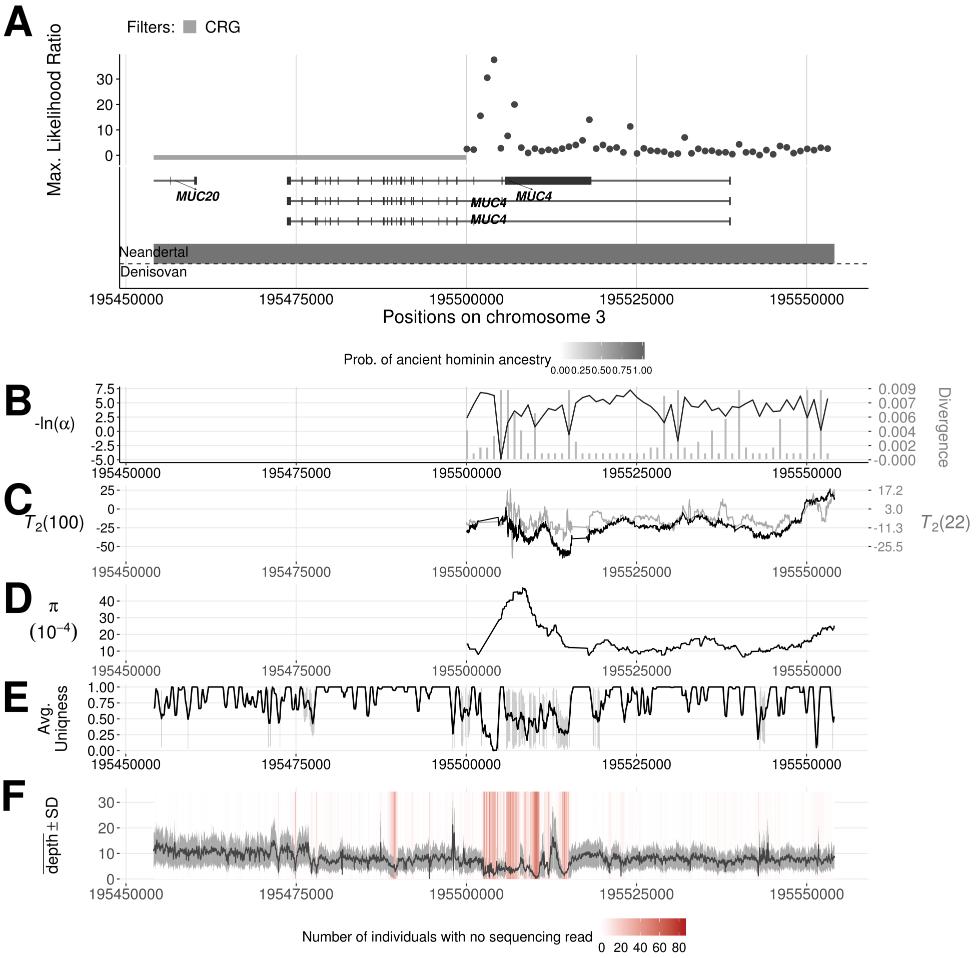

Fig. D9

Introgression sweep signals, parameter estimates, and sequencing properties across the 100 kb region on chromosome 19 covering the gene *CYP2B6* and *CYP2B7* in YRI. **A.** Likelihood ratio statistic computed from Model 1 of `VolcanoFinder` on the data of within-YRI polymorphism and substitutions with respect to the chimpanzee. Gray bars immediately below indicate the type of filters, and the longest gene transcripts are depicted with the wider bars standing for exons. **B.** Values for α and divergence D corresponding to the maximum likelihood estimate of the data. Black line corresponds to $-\ln(\alpha)$ and vertical gray bars correspond to estimated D. **C.** Likelihood ratio test statistic computed from T_2 of `BALLET` on data on within-YRI polymorphism and substitutions with respect to chimpanzee using windows of 100 (black) or 22 (gray) informative sites on either side of the test site. **D.** Mean pairwise sequence difference ($\hat{\theta}_\pi$) computed in five kb windows centered on each polymorphic site. **E.** Mappability uniqueness scores for 35 nucleotide sequences across the region. **F.** Mean sequencing depth across the 108 YRI individuals as a function of genomic position, with the gray ribbon indicating standard deviation. The background heatmap displays the number of individuals devoid of sequencing reads as a function of genomic position, with darker shades of red indicating a greater number of individuals with no sequencing reads.

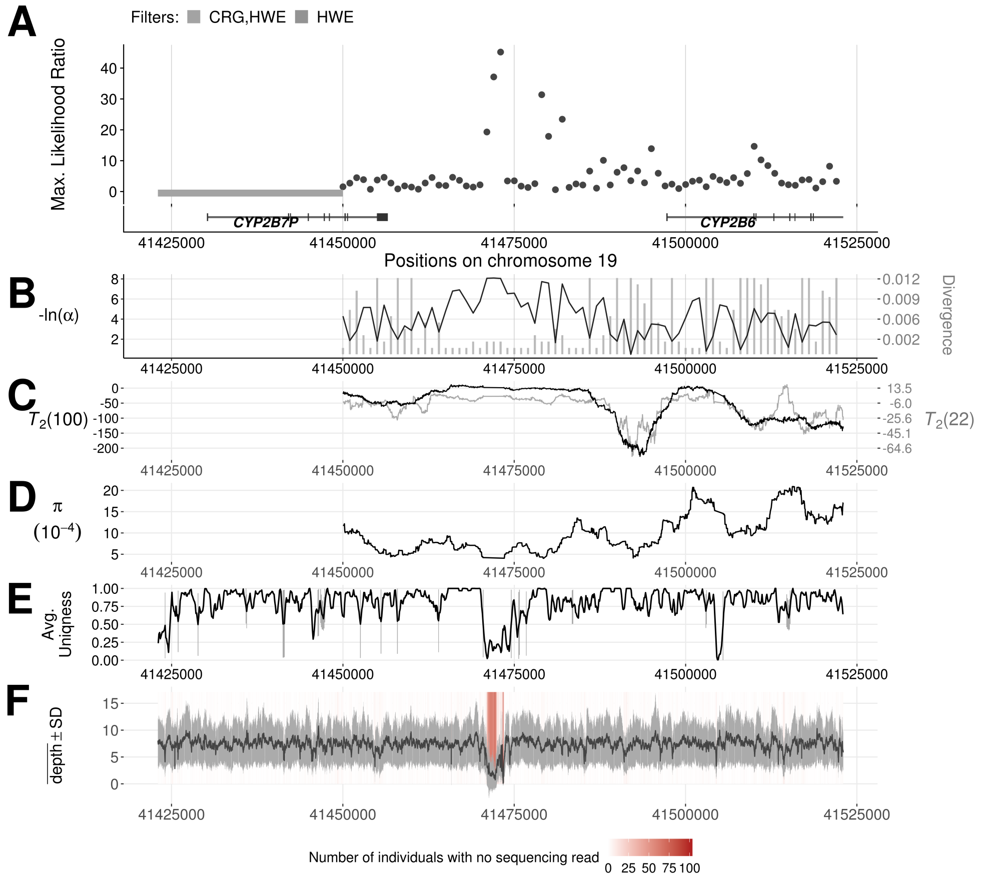

Fig. D10

Evidence for adaptive introgression on the one Mb genomic region covering gene *BNC2* in CEU.

A. Likelihood ratio test statistic computed from Model 1 of `VolcanoFinder` on data on within-CEU polymorphism and substitutions with respect to chimpanzee. Horizontal light gray bars correspond to regions that were filtered based on mean CRG and Hardy-Weinberg equilibrium (HWE) test. Gene tracts and labels for key genes are depicted below the plot, with the wider bars representing exons. Tracks of putative regions with Neanderthal (above the horizontal line) or Denisovan (below the horizontal line) ancestry are located below gene diagrams. Higher probabilities of Neanderthal or Denisovan ancestry are depicted with darker colored bands (data from [1]). Non-synonymous mutations with Neanderthal are indicated in red. **B.** Values for α and divergence D corresponding to the maximum likelihood estimate of the data. Black line corresponds to $-\ln(\alpha)$ and vertical gray bars correspond to estimated D. **C.** Likelihood ratio test statistic computed from T_2 of `BALLET` on data on within-CEU polymorphism and substitutions with respect to chimpanzee using windows of 100 (black) or 22 (gray) informative sites on either side of the test site. **D.** Mean pairwise sequence difference ($\hat{\theta}_\pi$) computed in five kb windows centered on each polymorphic site. **E.** Mappability uniqueness scores for 35 nucleotide sequences across the region. **F.** Mean sequencing depth across the 99 CEU individuals as a function of genomic position, with the gray ribbon indicating standard deviation. The background heatmap displays the number of individuals devoid of sequencing reads as a function of genomic position, with darker shades of red indicating a greater number of individuals with no sequencing reads.

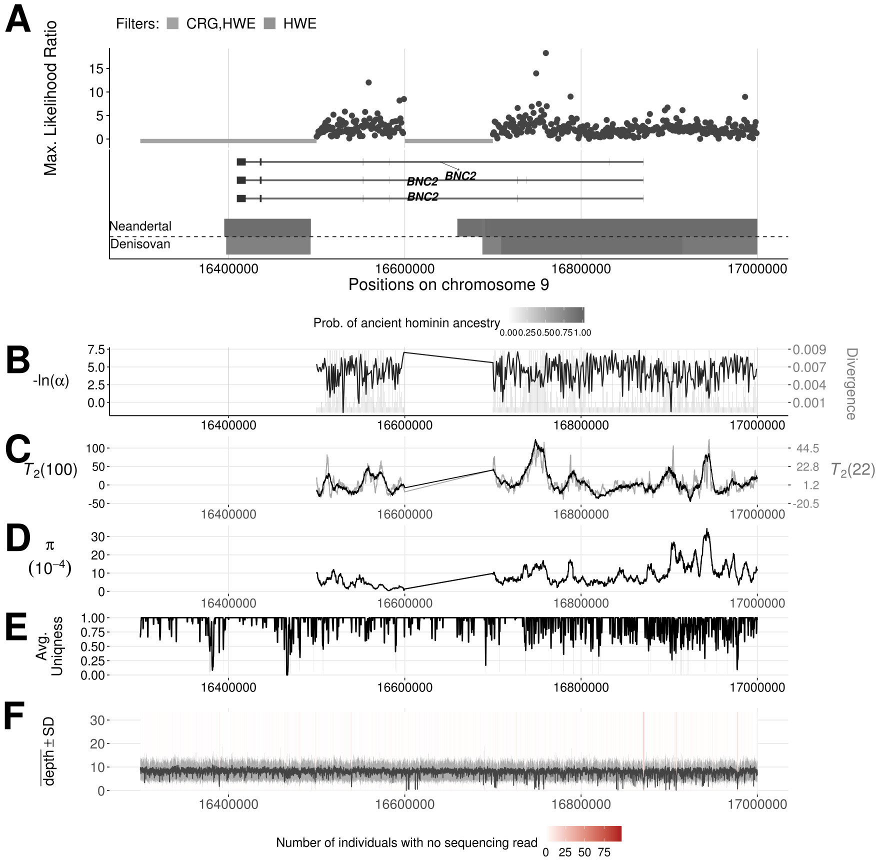

References

1. Sankararaman S, Mallick S, Patterson N, Reich D. The Combined Landscape of Denisovan and Neanderthal Ancestry in Present-Day Humans. Current biology : CB. 2016;26:1241–1247. doi:10.1016/j.cub.2016.03.037.
