# Supplementary material for: VolcanoFinder: Genomic scans for adaptive introgression
Source: PLoS Genet. 2020 Jun 18;16(6):e1008867. doi: 10.1371/journal.pgen.1008867 (PMC7326285; doi:10.1371/journal.pgen.1008867)
Supplement: Attachment — Submitted filename: vf_response_to_reviewers.pdf [file pgen.1008867.s005.pdf]

VolcanoFinder: genomic scans for adaptive introgression

Response to Reviewers

We would first like to thank the reviewers for their thoughtful and helpful comments. We feel that addressing the reviewers' concerns has substantially strengthened the manuscript and helped to make the presentation of the model and results much more clear. Below, we first summarize our most important changes and additions for the revised version and then provide answers to the detailed comments by the referees. We also provide a version of the revised manuscript with all major changes and additions highlighted in blue.

Most major comments by the referees pertain to the power analysis and ask for extended study of various biologically relevant scenarios. Indeed, we believe that all these suggestions merit further investigation and we have added considerable further work in this revision (see below). However, analyses across large parameter spaces with many replicates are computationally very demanding and limitations on our computational resources make it impossible to address all concerns in a realistic time frame. The analyses in the revisions alone required approximately 100 years of computing time. This is in addition to an even larger amount that went into the first submission of this manuscript. Below we summarize the additions that we made to the revised manuscripts and comment on any further constraints on the analyses.

A major criticism of our power analysis was that we used many independent neutral simulations as an approximation for the genomic background of the adaptive introgression allele. Rather, with recent advances in simulation methods, we can and should assess the power to detect the adaptive introgression allele in the context of a large chromosome. To address this point, we have set up a new (third) simulation procedure based on SLiM and msprime that allows for the simulation of larger genomic regions. There are, however, still some limits for detailed investigations that require many replicates. For simulation parameters representative of those in humans, we found that 10Mb chromosomes (corresponding to 20 centiMorgans) were the largest feasible. For a comparison, these simulations require approximately 6 hours per iteration, while a 20Mb chromosome required several days: longer than the standard run-time allowance on computing clusters. We therefore proceed with 10Mb chromosomes for most of our new analyses. In particular, we used these simulations to investigate

- the power of VolcanoFinder and SweepFinder for a simple introgression sweep scenario after hybridization with a single individual from a diverged donor population and the change in test power for larger amounts of genome-wide introgression (see Fig. 4),
- the dependence of test power on selection strength, divergence to the donor, density of test sites along the chromosome, and alternative choices for the detection of outlier peaks in the scan.
- In response to questions by the referees, we assessed the robustness of the method and the potential of classical selective sweep and background selection to produce false-positive signals for the detection of introgression sweeps (Fig. S4.6).
- Finally, we used the 10Mb simulations to validate our previous, purely coalescent-based approach that relies on comparison of a selected region with a concatenated genomic background that had been constructed from many smaller regions with independent

coalescent histories. We find that both approaches produce fully consistent results (Fig. S4.9).

For our extensive power analysis in the main text and new simulations for a human-inspired demographic history, even 10Mb simulations proved to be too time consuming. We therefore resorted to the purely coalescent-based approach using msms.

As support of our results from the human data scans, we performed an additional power analysis using the out-of-Africa human demography inferred in Gutenkunst et al. 2009 to estimate the power of VolcanoFinder to detect introgression from a donor species with Neanderthal-like divergence into present-day European populations (Fig. 6). Finally, we extended our discussion of the scope and limits of our method, also relative to previous approaches (lines 802-821).

Reviewer's Responses to Questions

Comments to the Authors:

Please note here if the review is uploaded as an attachment.

Reviewer #1: The authors present an excellent manuscript describing, testing, and applying an approach to detect adaptive archaic introgression. I only have a few comments about this strong manuscript:

1. Software: Copying and pasting the software example gives me the following error on a Mac OS X 10.12.6 (High Sierra) (this was observed in all examples in the manual):

```
../VolcanoFinder -i 800 psvf_2293_0242.txt spectvf_2300.txt -1 0 1 vf_2293_0242_2300.out
You have chosen to get introgression sweeps using pre-computed frequency spectra
done readsnps datasize=3059 nmax=40 nmin=40 xmax=41 invar=1
Initializing binomial coefficients
findsweeps smin=5.100000e+01 smax=9.999000e+04 gridsize=800 minlike=-inf
calcprobs nmax=40 nmin=40 xmax=41 invar=1
done calcprob
Assertion failed: (pr >=0.0 && pr<1.00000001), function ln_likelihood_introgression, file VolcanoFinder.c, line 980.
Abort trap: 6
```

We thank the reviewer for discovering this error. The error arises only when compiling the software on a Mac operating system. Unfortunately, we have, so far, not been able to identify

the cause of this problem. Therefore, at this time, we can only support running VolcanoFinder on Linux-based systems, and we have adjusted the software manual to reflect this.

2. Simulations: I would be interested in seeing results from a simulation with a demography inferred from human data (e.g. Gutenkunst et al 2009 or Gravel et al 2011) to see the effect of recent growth and low levels of migration between populations on the power of VolcanoFinder. In addition, there are several data sets that have small sample sizes (e.g. SGDP) where it would be interesting to see the results of VolcanoFinder. I would like to see a simulation examining the effects of low sample size on the power of VolcanoFinder (how low can one go?).

We have included a power analysis using the out-of-Africa demography inferred in Gutenkunst et al. 2009. Here, we consider an introgression event which occurs after the expansion into Eurasia, with fixation of the adaptive allele occurring just before the split into separate European and Asian populations. We consider a Neanderthal-like donor that diverged 615 kya, as well as two donor species with older divergence values (1.230 mya and 1.845 mya), and we consider both a genomic background with and without admixture (Fig. 6).

We did not include the effect of sample size on the power of VolcanoFinder in order to address other more pressing issues with the manuscript. However, we note that all power analyses in the main text were performed using a sample size of n=40 chromosomes, i.e. 20 diploid individuals. This is small enough to at least account for the sample sizes obtained across the major geographic regions in the SGDP datasets.

3. Discussion: Several papers have reported on signals of adaptive introgression (e.g. BNC2 (Vernot and Akey 2014, Sankararaman et al 2014), OAS (Mendez et al 2013; Sams et al 2016), and several signals from Gittelman et al 2016, Browning et al 2018 & Durvasula and Sankararaman 2019) and as far as I can tell there aren't any overlaps with these studies. Many of those studies used an allele/haplotype frequency cutoff to infer adaptive introgression rather than the more sophisticated approach used here so I suspect there are many false positives in those lists. In addition, the strict filtering used by the authors here could have masked out some of the previously found signals (thereby removing some of the false positives). A discussion of the lack of overlap would be interesting to readers.

The reviewer is correct to point out that our stringent filtering may have prevented the recovery of previously-reported candidates such as *BNC2*, *OAS1/2/3* cluster, and *TLR1/6/10* cluster (Dannemann *et al.* 2016). When inspecting our scan results on these genomic regions, we found that the majority of these regions are removed by the mappability filter. Further, as our models are more sensitive to strong and preferably complete sweeps on segments introgressed from a highly diverged donor, it is likely that previous candidates identified via haplotype-based approaches do not have their "volcano" features prominent enough to stand out among our candidates. For example, the well-supported introgressed region in *BNC2* scored ~18.3 in our CEU scan (Fig. S4.5.1), lower than our CLR cutoff value for identifying candidates. We have now added an extended discussion on this topic in the *Discussion and conclusions* section (lines 897-941).

Minor comments:

Labels on fig 5,6 should be bigger. Explain the X axis

The x-axis in these figures represents the number of false-positive peaks (k) from the neutral data that score higher than the true-positive signal of the adaptive introgression event. That is, these figures show the probability to detect the adaptive introgression allele among the (k+1) highest peaks that we choose to include into a list of genome-wide outliers. We have added a sentence to the figure legend to make this more clear, and we provide an expanded description of our approach in Text S4.1.

We have also edited the figures to improve readability.

Table S3.1 appears to be cut off

We have corrected this in the revised manuscript.

Typos:

Page 18, line 317 "he mutation rate" should be "the mutation rate" corrected

Page 25, line 451 "looses" should be "loses" corrected

Page 32, line 574 "apolipoportein" should be "apolipoprotein" corrected

Reviewer #2: Review of Setter, Mousset, et al.

In this manuscript the authors develop simple theoretical approximations to obtain the site frequency spectrum (SFS) from a model of adaptive introgression between species. With these approximations in hand, the authors then extend the sweepfinder machinery to look for adaptive introgression events using a composite likelihood estimator, named volcanofinder. The authors then benchmark the performance of volcanofinder and finally apply it to human data.

Generally I find this paper to be clearly written, quite timely, and the models are presented to be exceptionally clear. Most of my comments are aimed at improving the presentation even further, but I do have some additions that the authors should perform to strengthen the paper.

Major issues:

1) The authors are comparing the performance of volcanofinder to methods which are aimed at finding sweeps in single populations or balancing selection. While this is fine, the authors also need to compare volcanofinder to methods that are aimed at finding introgression writ large (with or without an adaptive sweep). I would suggest as a baseline S* from Jeff Wall or one of the newer supervised machine learning methods.

The software and models we chose for comparison in the analysis are not meant to show the power of VolcanoFinder in relation to other methods, but rather to demonstrate that VolcanoFinder is able to distinguish the local signal of an adaptive introgression event from both that of a non-introgressive sweep and that of long-term balancing selection. We use SweepFinder2 and BALLET primarily to cross-validate the presence or absence of a detectable signal when evaluating the robustness of VolcanoFinder. In contrast to the selection tests, tests for non-adaptive introgression, such as S*, should detect (when powerful) true positives along the whole genome in our simulations. They are thus answering a different question.

There has been a recent surge in methods aimed at identifying adaptive regions of genomic introgression, and we agree with the reviewer that a comparative analysis of these methods is sorely needed. This way, we also hope that our current method can still be improved, e.g. by including measures of LD. However, we believe that this is beyond the scope of this manuscript. In particular, our simulations provided only the polymorphism data needed for the methods we use, while many other methods require haplotype information or comparative genomic data. Given that over 100 years of computing time was needed for these revisions alone, generating this additional data is not feasible.

We have, however, expanded our discussion on the scope and limits of our method. In particular, we point out that we expect our method to be complementary to existing approaches and to have high power in different parameter regions (please see response to Reviewer 1, item 3 above.)

2) The authors also need to look at the robustness of volcanofinder to a few misspecified models that haven't been looked at, namely a single sweep in the focal population without introgression, and background selection. Both of these additions should follow the section that is titled "Robustness to long term balancing selection".

We have now included this in our analysis, as suggested.

To assess robustness wrt a classical sweep in a panmictic population, we used simulations of 10Mb chromosomes and compared the performance of VolcanoFinder to that of SweepFinder2. While VolcanoFinder has intermediate power to detect a strong sweep, it does not detect the signal of a weak selective sweep. This contrasts with SweepFinder2, which has very high power to detect the strong sweep and intermediate power to detect the weak selective sweep (see Text S4.3 and Fig. S4.6). We found that each method is 'specialized' to its primary scenario and has only residual power to detect the other type of sweep.

For background selection, we used SLiM3 to run forward-time simulations of a coding sequence, specifying for each element the rate at which deleterious variation occurs and a corresponding distribution of effect sizes. Each simulated region is approximately 1Mb in length with a genomic architecture defined by a randomly sampled stretch of annotated genome from the RefSeq database. A replicate neutral simulation was obtained for each genomic region by setting the strength of selection on all mutations to 0. We applied VolcanoFinder to the data and compared the distribution of test scores with and without background selection to determine the influence of background selection on the false positive rate of our test (for details see Text S4.4). We found that the action of background selection has little effect on the distribution of test

scores (Fig. S4.8). Most importantly, we did not observe outlier test scores from simulations with background selection relative to the distribution of scores taken from the neutral simulations.

3) The “outlier” study design as presented is unconvincing. The authors are treating the 10^4 200kb chucks they have simulated as a single genome on which to perform outlier studies. This is inappropriate as in truth as outlier study is performed on a genome where all chromosomes share a pedigree and each chromosome itself is a single tree sequence. I would encourage the authors to either abandon this section, or more preferably, to do proper chromosome-scale simulations on which to base an outlier test. Related: having the “number of peaks” as the x-axis on Figs 5&6 is difficult to interpret, although I understand (I think) what the authors are trying to show. This should be unpacked a bit more in the text if kept.

We agree that, ideally, we should evaluate the power of VolcanoFinder in this context, however, we are severely limited in this regard due to computational costs of these simulations (see our response to the editor above). Although they are not truly ‘long chromosomes’, we now include a limited power analysis using 10Mb genomic regions simulated using SLiM3 and msprime (Text S4.2). The basic results are the same: VolcanoFinder has high power when selection is strong but only low to moderate power when selection is weak (Fig. 4).

In addition to confirming our results in the main text, we use these simulations of 10Mb segments to demonstrate that data taken from independent simulation runs closely approximates that of a contiguous chromosome (Text S4.5 and Fig. S4.9). We do this by shuffling and resampling the genomic background variation among replicate simulation runs to create ‘chimeric’ chromosomes. The power to detect the adaptive introgression allele is effectively the same for the ‘chimeric’ chromosomes as for the original contiguous chromosomes from which they are built.

4) Lines 682-686—the authors are making too light of the fact that “neutral admixture” severely limits the power of volcanofinder. Indeed *any* adaptive introgression will be accompanied by an even larger amount of non-beneficial introgression, so this is a more appropriate null background to be working in. The authors should recast the paper in this light as this is the biologically meaningful scenario, not finding an introgressed region in a genome that has not encountered introgression.

It is true that any adaptive introgression event will be accompanied by a vastly larger amount of non-beneficial introgression. However, this does not imply that the remaining genome experiences purely neutral introgession. For highly divergent donor populations, as we consider in our model, much of the genome may be impermeable to gene flow between the two species. To some extent, this is even the case for a closely-related donor species, as has been shown for introgression from Neanderthals to modern humans (see Discussion section of the main text relating to background selection).

Both the case of no introgression and the case of genome-wide purely neutral introgression in the genomic background are unrealistic. They represent two extremes as a ‘best case’ and ‘worst case’ scenario with respect to the power of VolcanoFinder. In real data, we expect to see variability in the genome, with some regions permeable to introgression and others resistant. In that way, we provide a lower and upper bound with respect to the power of VolcanoFinder to detect the adaptive introgression sweep in a biologically realistic setting.

However, we do agree that in this figure could be quite misleading for the reader when included as part of the narrative of the main text, and for this reason, we have moved this material to the supporting information (Text S2.4 and Fig. S2.3).

Minor issues:

1) Line 45—the last sentence of this paragraph is quite cryptic. How could recurrent hybridization lead to a soft sweep exactly?

To better connect this to the effect of recurrent migration leading to soft sweeps from de novo mutation, we have modified this sentence as follows:

“In the same way that recurrent migration leads to soft sweeps from de novo beneficial mutations, recurrent hybridization during admixture events may result in soft sweeps of adaptive introgression alleles.“

2) Line 100—at this point the authors should explain what they mean by “complete lineage sorting in the ancestor” being assumed.

We have clarified this in the text as:

“We assume an infinite sites model and complete lineage sorting in the ancestor, i.e. coalescence in the ancestral population occurs only between one lineage from the outgroup and one lineage from the recipient (or donor) species.”

3) Line 141- “rd” should be defined here and its units made clear

This has been added to the text as follows:

“At a neutral locus linked to the the selected site, any pair of lineages currently associated with the *B* allele may coalesce at rate $1/(2NX[t])$, while any single such lineage may recombine to the *b* background at rate $R(1-X[t])$ per generation [51]. Here, R is the rate of recombination between the selected and neutral site, i.e. $R=r*d$, where r is the per-site recombination rate and d is the distance in base pairs.”

4) Lines 208-214. This section is unclear to this reader. Which this scaling issue is important the authors are not helping the reader to follow what is happening.

We have expanded upon this and referred the reader to Fig. S1.5 which shows the effect of the selective sweep with respect to this scaled distance measure. The text now reads:

“We can analyze the shape of the footprint in more detail using the star-like approximation. In this case, the width of the signal can be measured in terms of a single compound parameter $\alpha d = R \log(2N)/s$. This compound parameter is a generalized description of the effect of a sweep along the genome, as distance from the sweep center is measured relative to the strength of selection. The top panel of Fig. S1.5 shows the effect of the adaptive introgression sweep on genetic diversity as a function of αd. When αd is near 0, diversity is reduced relative to the background, while at distance $\alpha d \approx 1$, we see the peak of

the volcano pattern. At distances $\alpha d \approx 6$, the sweep has a much smaller effect and diversity is only slightly higher than the genomic background."

5) Line 344—this section on the SFS should be moved either to the supplement, or directly after the model is introduced. This is a strange bit to have here.

We have moved this to the supporting information (Text S1.3 and Fig.S1.3).

6) Line 346—should say the sample size clearly here This has been clarified.

7) Line 382—unclear at this point in the text what is meant by the "95% probability" of a sweep. This should be made clearer. Also can't the authors just condition on the sweep having occurred?

For the power analysis, these simulations were performed using the coalescent simulator msms, and unfortunately, it is not possible to condition on fixation of the beneficial mutation for non-panmictic demographic models. We have therefore chosen parameter values for the selection strength and the migration rate at admixture that lead to establishment and fixation of the introgressed beneficial allele in 95% of the cases. We have included additional power analyses in the revisions (see above) that use msprime and SLiM3, allowing us to condition on fixation of the beneficial mutation.

8) Lines 432-438. A simpler way to present this information would be to just give the AUCs for the ROC curves.

We presented ROC curves as this is the standard way to display test power in the population genomic literature. Also, ROC provides the more complete information (AUC is implicit to ROC, but not vice-versa). We therefore decided to keep it this way.

9) Lines 461-468—again wondering why the authors just don't throw out the simulations without a sweep. It would make this section of the paper much easier to the naïve reader.

As mentioned above, conditioning on fixation is not possible for these simulations, which is why we resorted to this solution. Note that our more limited simulations (because of computation time) using SLiM and msprime in the revised version do allow for conditioning and show fully consistent results.

10) Lines 545-547—It would be nice if the overlap or lack thereof in the manhattan plots were quantified. What percentage are the same? What is the expectation under independence?

Unfortunately, there is no quantitative way to compare the results from these two data sets. Even a broad qualitative comparison is difficult because the distribution of test scores is specific to each data set: the overall scores from YRI are generally much lower than those of CEU. Rather, we comment on the overlap of the candidate regions that we identified in the downstream analysis of the VolcanoFinder scans (e.g. both show a strong signal at the APOL gene region).

11) Line 556 and following—this is not and McDonald-Kreitman test per se. It is a 2x2 contingency test of polymorphism and divergence however.

Yes, this is rather an HKA test, and we have corrected this in the text.

12) Line 588—it is unclear what the authors mean when they say that the a region does not "exhibit high CLR scores despite the region devoid of data". If all missing data causes high CLR scores shouldn't the authors simply adjust the output with a heuristic that says there is too little data here to calculate a CLR?

We often see highly-elevated test scores for sites that fall within a region of missing data, e.g. a centromere, because to VolcanoFinder, this looks like a large region with no polymorphism. Using a very strong sweep strength and low divergence, VolcanoFinder can model this trend in the data as a sweep and explains the data vastly better than the neutral model, generating the inflated composite likelihood ratio value. Though the effect is much smaller, we also see a trend toward higher test scores for sites adjacent to such regions. While we can easily exclude test sites within or near large regions of missing data, it is less clear when to exclude sites near smaller regions of missing data.

In order to assess whether there is sufficient data to support VolcanoFinder's claim, we must examine the region of the data that is included in the likelihood calculation. For the reported sweep strength α, this includes data up to distance $d = 12/\alpha$. The inferred sweep at *APOL4* in the YRI population does extend over a region of missing data, however, there is also high-quality data informing the test statistic at these sites.

That this region of missing data does not cause inflated likelihood ratio values in the CEU population supports the validity of the signal in YRI, however we chose to be very stringent when assessing and identifying the 'top candidate' regions from the data. We present the *APOL4* region of both YRI and CEU as this region provides an illustrative example of candidate assessment for readers who choose to use our method.

We have adjusted the text to clarify this as follows:

"Note that this candidate was not included in our final list of candidates for the YRI population due to the lack of data close to *APOL4*. The concern is that test scores can be inflated near regions devoid of data. Although the breadth of the sweep as predicted by VolcanoFinder includes one such region, there is also high-quality data informing the test statistic at these sites. Furthermore, the lack of data in this region does not result in high CLR scores in CEU, lending support to the validity of the signals we observe in the scan on YRI."

13) Line 733—this is a strange collection of papers to be citing for using ML for finding introgression

Here we failed to clearly distinguish the machine-learning methods from the maximum-likelihood methods. The citations are unchanged, and we have clarified this in the text as:

“In addition, machine-learning algorithms provide a likelihood-free approach to detecting footprints of introgression when trained using data simulated under a particular demographic model [20,22,25]”

14) Line 950—all code should be deposited on a public repository. Software being “available upon request” is not acceptable in 2019.

We have now made the code available on Dryad and included this in the submission.

Reviewer #3: This study was motivated by an increasing number of adaptive evolution discovered to be driven by positive selection on an introgressed variant. They found analytic approximation for the volcano pattern of polymorphism and turned it into a statistical method for genome scan. I found this study very timely and rigorous. I do not have any major point for criticism. Although this manuscript might be unnecessarily long, I think it is ready to be published after revisions to address the following minor issues.

My comments:

1. Lines 239-247. It will be nice if it is mentioned which specific panels in Figure 3 this section is talking about. In addition, “all B lineages” (line 241) might be better changed to “all B-linked lineages”, because whether a given lineage is B or b seemed to be defined in term of the final state shown in Table 1.

We have adjusted the text to ‘B-linked’ as suggested. We also now refer to examples of these effects among the single iterations of the adaptive introgression process shown in Figure 3.

2. It was initially confusing to follow the derivation of SFS on page 16 because, I think, the assumption of infinite site model (on the entire genealogy linking recipient and outgroup sequences only one mutation event can be mapped) and exclusion of sites that are invariant over both recipient and outgroup was not emphasized enough. Only under that assumption, S_0(n) is understood as the probability of derived allele on the outgroup only.

We have tried to make this more clear in the revised text as follows:

Consider an alignment of n sequences from the recipient species and one sequence from an outgroup species to polarize the data. In the recipient population, we observe a mutation with frequency $i=1,2,...,n$ with probability $S_i(n)$, where $S_n(n)$ is the probability of observing a fixed difference relative to the outgroup. The $S_i(n)$ represent the non-normalized SFS, i.e. the probability of a monomorphic site is $1-\sum_{i=1}^{n} S_i(n)$. If we sample a second more distant outgroup, under the assumptions of complete lineage sorting and the infinite sites mutation model, we can further distinguish the lineage on which the fixed differences occur. In this case, $S_n(n)$ is the probability that the mutation occurred specifically on the lineage ancestral to the recipient population, and we denote by $S_0(n)$ the per-site probability of observing a mutation private to the first outgroup lineage. That is, the probability of observing a fixed difference is $S_0(n)+S_n(n)$. If a second outgroup is unavailable, then only polymorphic

mutations in the recipient species can be polarized, but not the fixed differences. In this case, we arbitrarily label the state in the first outgroup as ``ancestral'' such that $S_0(n)=0$.

3. line 317, he -> the. Corrected

4. line 374. I believe it is Text S2.4, not S1.3.. Corrected.

5. line 430. How "peaks" are defined is an important issue and should be briefly mentioned in Result. In Methods, the definition is not consistent: it is either a separation by less than 10 LR values (line 1053) or a fixed value of 15kb as minimum distance between peaks (line 1080). I wonder whether a better (logical) way of merging sites of significant LRs into a peak can be devised. For example, the minimum distance between peaks might be given proportional to the estimated strength of selection (s^).

We agree that defining peaks is an important issue, and the idea of using the sweep breadth is a good one. Indeed, we initially considered using the sweep distance as a means of identifying peaks. We decided on this alternative peak-finding method in order to make clear comparisons with other outlier-based methods in which sweep-width cannot be used to identify peaks.

We have included additional power analyses in the revisions in which we simulate 10Mb genomic regions (see above). Here we investigate the power to detect the adaptive introgression allele in the context of the surrounding genome. In this context, we agree that the sweep-width is the best way to identify independent peaks and to identify the true positive signal. We provide a more complete description about these approaches in Text S4.1, and we also show that the two approaches yield consistent results (compare Fig. S4.9 to Fig. S4.1)

6. lines 490-502. The effect of varying window size for a given Ns was not shown, not in Fig. S2.7.

This text has been moved to the supplement (Text S2.5) and we have clarified this in the text and the figure legends. The first two ROC curve figures (Fig. S2.6 and Fig. S2.7) use the highest score in the 200 kb window for both weak and strong selection. Fig S2.8 alone uses a 20 kb window, and in this case, only for weak selection. The reader should compare the case of weak selection in Fig S2.7 to Fig S2.8.

7. line 542. Why non-synonymous differences only? Isn't it more informative to use both synonymous and non-synonymous differences in finding introgression candidates?

We agree with the reviewer that both synonymous and non-synonymous differences would be informative. However, as the dataset we obtained (from UCSC Table Browser) was generated in the study by Burbano *et al.* (2010), in which the authors determined coding changes by comparing protein sequences, only non-synonymous substitutions are included. Further, this study also only included genes with one-to-one homology mapping between humans and chimpanzees, which further reduced the pool of available sites. We have now added the details in the *Materials and Methods* section (lines 1221 to 1229).

Burbano HA, Hodges E, Green RE, Briggs AW, Krause J, Meyer M, Good JM, Maricic T, Johnson PL, Xuan Z et al. (2010) Targeted investigation of the Neandertal genome by array-based sequence capture. *Science*. (5979):723-5.

8. line 556 and others. I think it is more appropriate to call it HKA (Hudson-Kreitman-Aguade) test rather than MK test.

Yes, it should be the HKA, and we have corrected this.

9. line 566. The top rows of Table S3.1 are invisible in the manuscript file.

This has been corrected in the revised version of the manuscript.

10. line 581. Apolipoprotein Corrected

11. It is difficult to follow lines 586-591. Fig S3.3A -> Fig 3.3F (?). What does it mean by “despite the region devoid of data” in CEU?

We have clarified this in the text. Please see the response to Reviewer 2, item 12 above.

12. I think Discussion and conclusion can be shortened. Throughout the manuscript, similar information is given repetitively. For example, lines 1043-1049.

We realize that this manuscript is quite long, and we have tried to make adjustments without increasing the length too much with the revisions material.

13. Maybe a direction of further development, such as detection of incomplete sweep of introgressed variant, can be mentioned?

We have remarked on extensions to the model at the end of the discussion section, namely, accounting for for sweeps which occurred farther pastward in the underlying model as well as the inclusion of linkage information in the composite likelihood framework could provide substantial improvements in the power of VolcanoFinder (see lines 802-821).
